# Hydroxytyrosol Mitigates Anxiety‐Like Behaviors After a Traumatic Experience in Aged Mice in Parallel With Increased Neurogenesis in the Ventral and Dorsal Dentate Gyrus, and Preservation of Gut Microbiota Composition

**DOI:** 10.1111/jnc.70448

**Published:** 2026-04-30

**Authors:** Giorgio D'Andrea, Laura Bertini, Marco Costanzi, Fabiana Canini, Roberta Bernini, Andrea Fochetti, Mariangela Clemente, Silvia Proietti, Manuela Ceccarelli, Carla Caruso, Maurizia Caruso, Ferdinando Scavizzi, Marcello Raspa, Felice Tirone, Laura Micheli

**Affiliations:** ^1^ Institute of Biochemistry and Cell Biology (IBBC), National Research Council of Italy (CNR) Rome Italy; ^2^ Department of Ecological and Biological Sciences University of Tuscia Viterbo Italy; ^3^ Department of Human Sciences LUMSA University Rome Italy; ^4^ Department of Agriculture and Forest Sciences (DAFNE) University of Tuscia Viterbo Italy; ^5^ Onco‐Hematology, Cell Therapy, Gene Therapies and Hemopoietic Transplant, Bambino Gesù Children's Hospital IRCCS Rome Italy; ^6^ Institute for Sustainable Plant Protection National Research Council of Italy Torino Italy; ^7^ Institute of Biochemistry and Cell Biology National Research Council of Italy (IBBC‐CNR/EMMA/INFRAFRONTIER/IMPC) Rome Italy

**Keywords:** aging, hydroxytyrosol, microbiota‐gut‐brain axis, neurogenesis, olive oil, post‐traumatic stress disorder

## Abstract

Hydroxytyrosol (HTyr), a phenolic compound present in olive oil, exhibits antioxidant, anti‐inflammatory, and neuroprotective properties, benefiting several age‐related diseases. Our previous research demonstrated that oral HTyr administration counteracts age‐associated neurogenesis decline in the dentate gyrus of the hippocampus by promoting the production of stem/progenitor cells and new neurons. Since new neurons generated in the dorsal dentate gyrus support contextual memory discrimination, while those generated in the ventral region modulate anxiety, we investigated whether pure HTyr, synthesized in our laboratories, selectively stimulates neurogenesis in these regions in aging mice and evaluated its effects on contextual memory and stress response. Furthermore, we examined its influence on gut microbiota composition, given the well‐established role of the microbiota‐gut‐brain axis in memory and stress regulation. We found that HTyr induced the production of new neurons and neuroblasts in both dentate gyrus regions, with a prevalent effect in the ventral region. Consistently, we observed that HTyr treatment did not improve the contextual memory discrimination but reduced fear sensitization and anxiety‐like behavior after a traumatic experience. Furthermore, we observed a reduction of neuroinflammation in HTyr‐treated dentate gyri. In parallel, treatment with HTyr preserved the stability of key microbial families linked to intestinal well‐being, counteracting the unhealthy effects of stress on gut microbial structure. Our results suggest that HTyr treatment in aging mice enhances resilience to posttraumatic stress by increasing neurogenesis and modulating the microbiota‐gut‐brain axis. Future studies should explore its potential as a therapeutic intervention for individuals experiencing posttraumatic stress disorder symptoms.

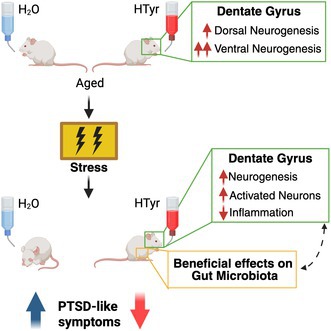

## Graphical abstract

Hydroxytyrosol (HTyr), a phenolic compound of olive oil, exerts beneficial effects on several age‐related diseases. We previously demonstrated that oral HTyr administration in aging mice enhances hippocampal neurogenesis, known to play a role in memory formation and emotional behaviors. In the present study, we found that HTyr preferentially stimulates the neurogenesis of the ventral hippocampus, reduces anxiety‐like behavior in a model of post‐traumatic stress disorder (PTSD), attenuates neuroinflammation and preserves the stability of key microbial families linked to intestinal well‐being. These findings suggest that HTyr promotes resilience to posttraumatic stress by modulating neurogenesis and the microbiota‐gut‐brain axis.

**Abbreviations**
ANOVA: analysis of variance
ASVs: amplicon sequence variants
BrdU: 5‐bromo‐2′‐deoxyuridine
CFC: contextual fear conditioning
CFD: contextual fear discrimination
Cy2: cyanine2
Cy3: cyanine3
DCX: doublecortin
DG: dentate gyrus
EVOO: extra virgin olive oil
F/B ratio: firmicutes/bacteroidetes ratio
FS: fear sensitization
HTyr: hydroxytyrosol
Iba 1: ionized calcium‐binding adapter molecule 1
l‐DOPA: l‐3, 4‐dihydroxyphenylalanine
LDA: linear discriminant analysis
LEfSe: LDA effect size
NeuN: neuronal nuclei
OF: Open Field
PBS: phosphate buffered saline
PFA: paraformaldehyde
PLSD: protected least significant difference
PM: Plus Maze
PTSD: post‐traumatic stress disorder
RRID: Research Resource Identifier
SCFA: short‐chain fatty acid
SEM: standard error of the mean
SGZ: subgranular zone
Sox2: sex determining region Y‐box
TRITC: tetramethylrhodamine

## 1 Introduction

Aging‐associated cognitive decline is becoming an important risk factor for health due to the increasing human lifespan. A balanced diet is one of the most critical elements in maintaining brain function and cognitive capacity during aging. Indeed, dietary components can modulate neuronal function, synaptic plasticity, and adult neurogenesis, that is, the process by which new neurons are generated during life within neurogenic niches: the subgranular zone (SGZ) of the dentate gyrus of the hippocampus and the subventricular zone (SVZ) (Cameron et al. 1993). Diet thus influences cognitive capacities, particularly those that are hippocampus‐dependent (Stangl and Thuret 2009). The hippocampus is important in emotional behavior, regulation of hypothalamic functions, and encoding and retrieval of different contextual, spatial, and temporal information, so that memories may be effectively recovered and associated (Rolls and Kesner 2006; Aimone et al. 2011). This function is enhanced by the generation of new neurons from radial glia‐like stem cells in the SGZ of the dentate gyrus throughout life (Seri et al. 2001; Filippov et al. 2003; Kronenberg et al. 2003; Komitova and Eriksson 2004). Stem cells develop into progenitor cells, and then into neuroblasts (Filippov et al. 2003; Kronenberg et al. 2003; Fukuda et al. 2003; Steiner et al. 2006), from which early postmitotic and mature neurons originate (Brandt et al. 2003; Steiner et al. 2006). Newly born neurons, being more responsive until they are 4‐ to 6‐week‐old, enhance the resolution between different memories encoded by mature neurons (Aimone et al. 2011; Farioli‐Vecchioli et al. 2008; Tirone et al. 2013).

Notably, neurons produced in the dorsal part of the dentate gyrus are mainly responsible for contextual discrimination memory, in particular in the execution of those tasks, such as contextual fear conditioning, in which the ability to discriminate among potentially overlapping experiences is required (i.e., pattern separation) (Kjelstrup et al. 2002; França et al. 2017; Anacker and Hen 2017; Fölsz et al. 2023). Instead, neurons produced in the ventral part are more involved in the control of anxiety (Bannerman et al. 2004; Anacker et al. 2018) as they are related to the limbic system and amygdala, which play a central role in fear. In fact, the ablation of adult new neurons in the dorsal dentate gyrus impairs contextual discrimination (Wu and Hen 2014). On the other hand, optogenetic activation of neurons in the ventral dentate gyrus causes a reduction in anxiety‐like behavior (Kheirbek et al. 2013). The discovery of stress‐responsive cells in the ventral dentate gyrus, regulated by adult‐born neurons, directly correlates stress to neurogenesis and is in line with previous data showing that chronic stress inhibits neurogenesis in the dentate gyrus (Czéh and Lucassen 2007).

There is evidence showing that adult hippocampal neurogenesis promotes associative memory while acting as a buffer against anxiety caused by fear conditioning tasks (Seo et al. 2015). Indeed, it is known that contextual fear conditioning task leads to the formation of both an associative (i.e., contextual memory) and a non‐associative memory (i.e., fear sensitization). Fear sensitization is largely independent from the contextual memory, increases as a function of time after the traumatic experience (fear incubation) and is mainly responsible for the development of anxiety‐like behaviors that resemble post‐traumatic stress disorder (PTSD) symptoms in mice (Kamprath and Wotjak 2004; Siegmund and Wotjak 2007a, 2007b; Costanzi et al. 2011, 2014).

During aging, the generation of progenitor cells and new neurons in the dentate gyrus declines progressively, but there is evidence of their persistence, albeit reduced, also in aged humans (Boldrini et al. 2018; Dumitru et al. 2025). This decline is associated with reduced associative and spatial memory and sensory functions (Vivar 2015; Bizon and Gallagher 2003; Bettio et al. 2017; Babcock et al. 2021; Micheli et al. 2018). Certain stimuli can counteract the reduction in neurogenesis during aging, such as voluntary exercise (van Praag et al. 1999; Wu et al. 2015), while others, such as learning (Gould et al. 1999; Kempermann et al. 1998) and antidepressants modulating serotonin (5‐HT) or norepinephrine (Santarelli et al. 2003; Malberg et al. 2000; Couillard‐Després et al. 2009), are able to elicit neurogenesis in adults but not in aged individuals.

The existence of a bidirectional microbiota‐gut‐brain axis, which exerts considerable influence on host behavior and cognition, is now widely recognized (Cryan and Dinan 2012). In fact, the disruption of the intestinal microbiota balance (dysbiosis) occurring in neurological diseases and during physiological aging can lead to neuroinflammation and a reduction in hippocampal neurogenesis accompanied by anxiety‐like behaviors and impaired learning (Liu et al. 2022). Microbial‐derived metabolites, such as short‐chain fatty acids (SCFAs), and gaseous signaling molecules have been shown to reduce inflammatory signaling and modulate the levels of neurotrophic factors, thus increasing neurogenesis and improving neuronal homeostasis and function (Abdel Haq et al. 2019; Kundu et al. 2019). Furthermore, metabolites produced by the gut microbiota also include some neurotransmitters or their precursors, which can potentially influence microglial activation and several cerebral functions (Strandwitz 2018).

With respect to dietary factors, the Mediterranean diet is known to exert neuroprotective and antiaging effects, also influencing the gut microbiota (Barber et al. 2023). In particular, extra virgin olive oil (EVOO) contains natural antioxidant compounds, such as unsaturated fatty acids and phenolic compounds, which beneficially impact human health (Martinez‐Lapiscina et al. 2013; Stangl and Thuret 2009; Ceccarelli et al. 2020; Romani et al. 2019).

There is evidence that the phenolic compound 3,4‐dihydroxyphenylethanol (hydroxytyrosol; HTyr) is the main component responsible for the neuroprotective, cardiovascular, anticancer, and metabolic effects of EVOO (Bernini et al. 2013, 2015; Konstantinidou et al. 2010; de Pablos et al. 2019). The biological activity of HTyr and other phenolic compounds has been attributed mainly to their antioxidant potential (Raederstorff 2009; De La Cruz et al. 2015). In fact, HTyr has been reported to protect the brain from oxidative stress and inflammatory mediators (De La Cruz et al. 2015), from spinal cord‐induced oxidative stress (Zhang et al. 2019), or in vitro from 6‐hydroxydopamine‐induced damage, l‐DOPA or hydrogen peroxide toxicity (Funakohi‐Tago et al. 2018; Omar et al. 2018; Peng et al. 2015), and to promote autophagy against oxidation by regulating SIRT1 (Sun et al. 2019; Velotti and Bernini 2023). Moreover, HTyr treatment in a mouse model of Alzheimer's disease has been shown to cause a significant reduction in the amyloid‐β plaque number in hippocampal areas (Nardiello et al. 2018). Interestingly, HTyr administration was also found to be able to modulate the colonic microbiota in mice, often enhancing the synthesis of beneficial microbial metabolites. For example, HTyr significantly enhanced the colonic butyrate concentration in diquat‐challenged mice (Han et al. 2023) and increased the levels of SCFAs in mice subjected to dextran sodium sulfate‐induced colitis (Miao 2022).

We have recently demonstrated by an in vivo study that HTyr is able to reactivate the production of stem/progenitor cells and new neurons in the dentate gyrus of aging mice (D'Andrea et al. 2020). This finding is very peculiar since only the strongest neurogenic stimuli are able to reactivate aged stem cells (Ceccarelli et al. 2020). A possible mechanism for the effect of HTyr on neurogenesis may lie in its ability to modulate the oxidative state of stem cells, as it has been shown that the shift from a high to a low oxidative state represents a proliferative signal for stem cells (Adusumilli et al. 2021).

In this work, we further investigated the effects of HTyr on the neurogenesis occurring in the dorsal and ventral region of the dentate gyrus in aging mice. Furthermore, given the dual implications of adult dentate gyrus neurogenesis in both memory and stress, we asked whether the neurogenic action of HTyr can protect against the aging‐related loss of contextual memory discrimination and from the development of anxiety‐like behaviors induced by a stressful experience (using a model of post‐traumatic stress disorder, PTSD). Finally, we asked whether, in our model system, HTyr affects the gut microbiota composition.

## 2 Materials and Methods

### 2.1 Mouse Line, Genotyping, Husbandry, Hydroxytyrosol (HTyr) and Bromodeoxyuridine Treatments

C57BL/6J mice (obtained from the European Mouse Mutant Archive‐EMMA; Monterotondo Scalo, Rome, Italy) were maintained under standard specific‐pathogen‐free conditions and had ad libitum access to food and water. Animals were housed in pairs (two animals per standard cage) until 3 or 15 months of age and at the beginning of the experimental procedures they were singly housed. Then, to reduce baseline variability, 15‐month‐old mice, identified by distal phalanx amputation, were weighed and manually assigned to the untreated/control group (H_2_O, maintained with drinking water) or treated group (HTyr, administered with hydroxytyrosol in drinking water), using stratified allocation based on body weight. No formal randomization was applied. No exclusion criteria were predetermined.

**FIGURE 1:**
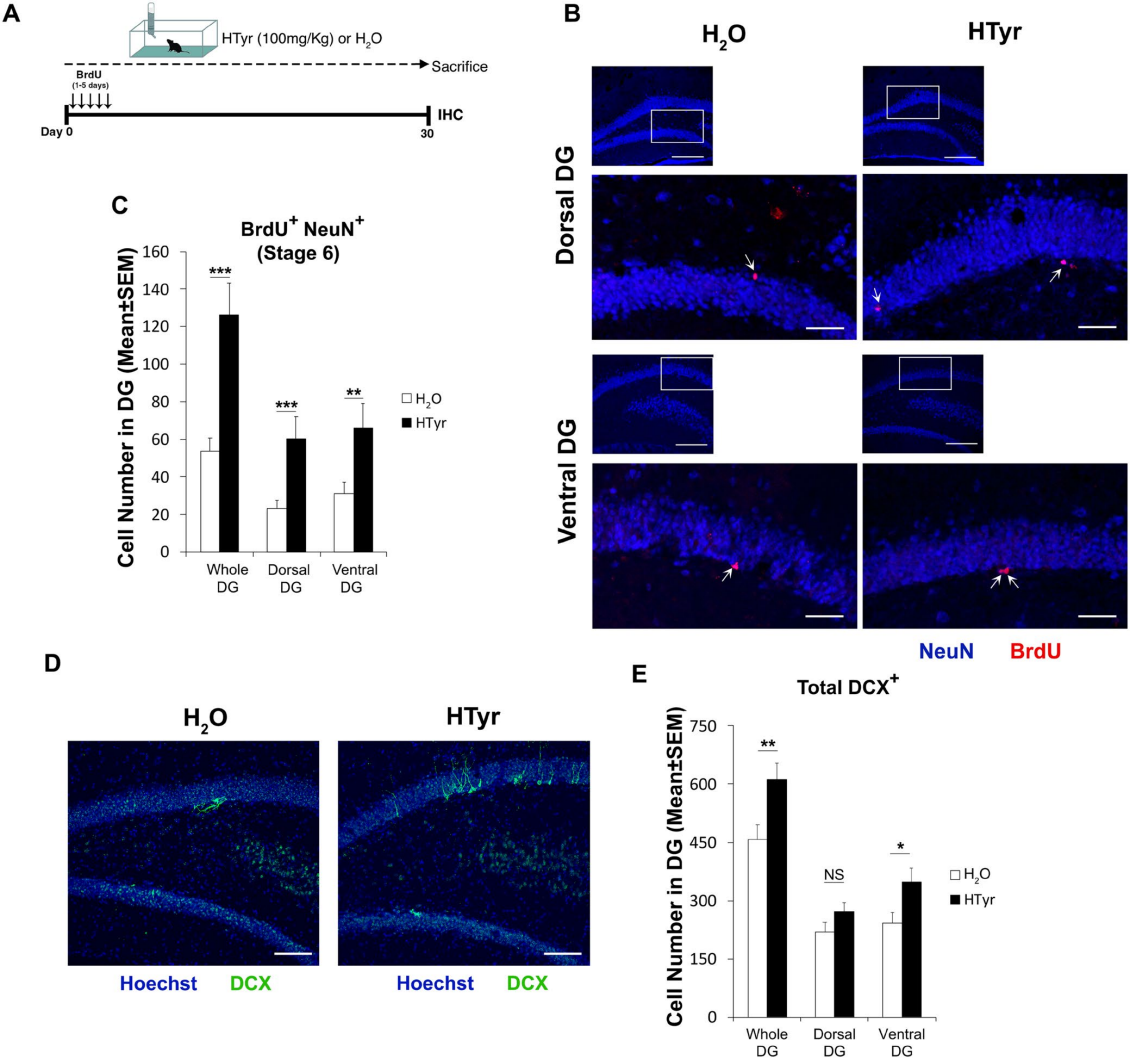
Thirty days of HTyr treatment promotes neurogenesis both in the dorsal and ventral DG. (A) Schematic representation of the experimental plan. Fifteen‐month‐old C57BL/6J mice were treated with standard water (H_2_O, control group) or with HTyr 100 mg/kg for 30 days; mice received a daily injection of BrdU (95 mg/kg) for the first 5 days of treatment. (B) Representative images by confocal microscopy showing 26‐ to 30‐day‐old new neurons (in red BrdU^+^ and in red/blue BrdU^+^/NeuN^+^ cells), for each condition. On the top, images (20×) of dentate gyri labeled with NeuN to visualize the position along the dorsal/ventral axis (scale bar, 200 μm). The white box area is shown below with 3× digital magnification. Scale bar, 50 μm. Arrows indicate double‐labeled cells. (C) In the whole DG, the total number of newly formed neurons (stage‐6 neurons, BrdU^+^/NeuN^+^) was augmented by treatment with HTyr in aged mice (fixed effect: Treatment HTyr vs. H_2_O, *n* = 141 DG sections; 8 mice, ***p < 0.001, GLMM‐NB, Wald *χ* ^2^ test). A comparable effect was observed along the dorsoventral axis (planned simple effects of Treatment: D‐DG ***p < 0.001; V‐DG **p < 0.01, Wald *χ* ^2^ test). The number of cells in the D‐DG or V‐DG was quantified in at least 31 sections of dentate gyri from four mice per experimental group. (D) Representative confocal microscopy images showing total DCX^+^ cells in ventral dentate gyrus (20×; scale bar, 100 μm). (E) The total number of DCX^+^ cells was increased by HTyr treatment in whole DG (fixed effect: Treatment HTyr vs. H_2_O, *n* = 130 DG sections; 8 mice, **p < 0.01; Wald *χ* ^2^ test) and in particular in the ventral region (planned simple effects of Treatment: D‐DG NS *p* > 0.05, V‐DG *p < 0.05). The number of cells in the D‐DG or V‐DG was quantified in at least 27 sections of dentate gyri from four mice per experimental group. The graphs in panel C and E represent the means ± SEM of individual cell counts. Statistical analysis was performed using a GLMM‐NB model with Mouse as random intercept. D‐DG, dorsal dentate gyrus; GLMM‐NB, generalized linear mixed model‐negative binomial; IHC, immunohistochemistry; V‐DG, ventral dentate gyrus.

**FIGURE 2:**
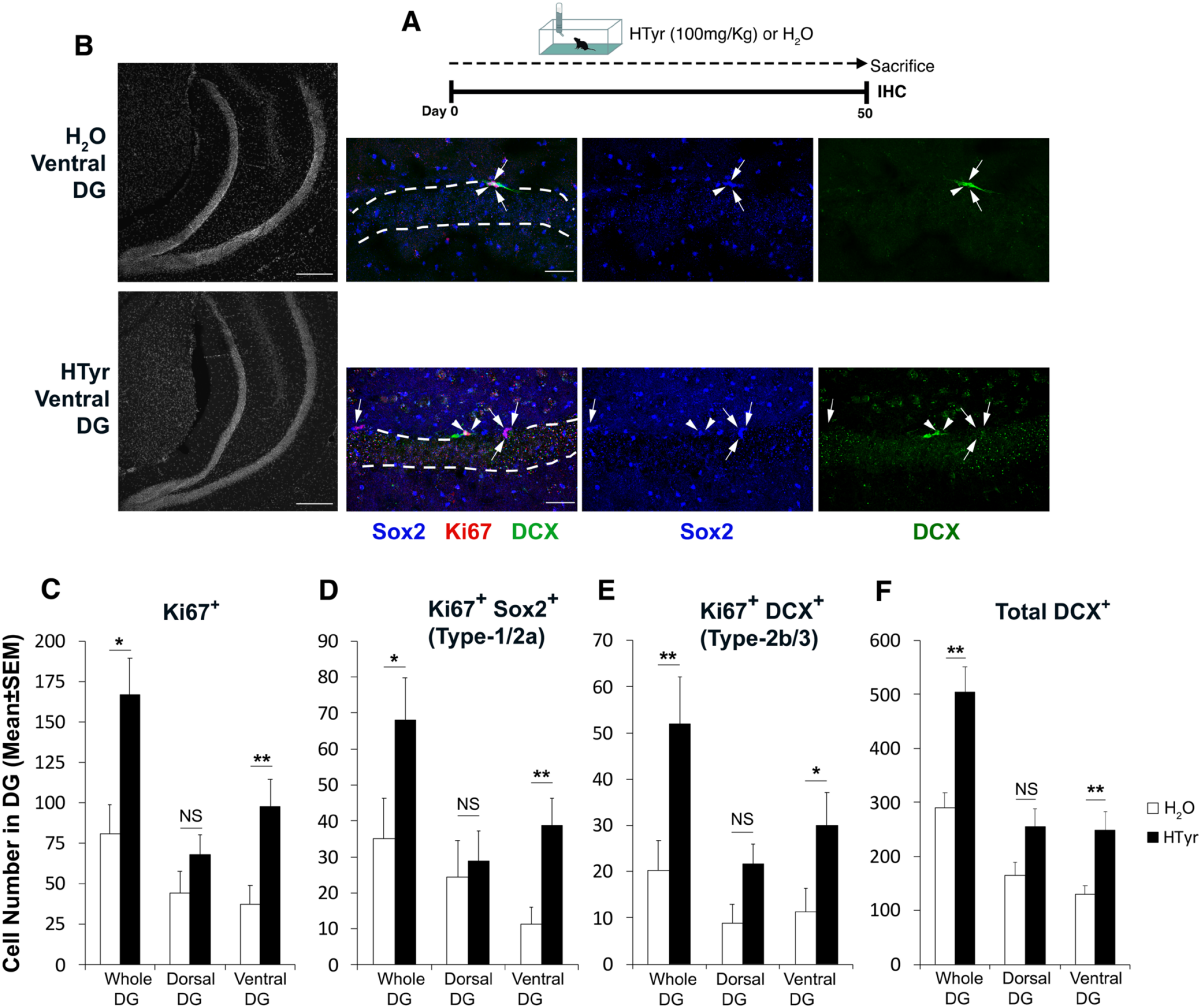
Prolonged HTyr treatment promotes proliferation of stem/progenitor cells in the ventral DG. (A) Experimental design: 15‐month‐old C57BL/6J mice were treated with water (H_2_O) or HTyr (100 mg/kg) for 50 days. (B) Fluorescence confocal images (40×) showing type‐1‐2a stem/progenitor cells (Ki67^+^/Sox2^+^; in red/blue, indicated by arrows) and type‐2b‐3 progenitors (Ki67^+^/DCX^+^; in red/green, indicated by arrowheads) in the ventral DG of HTyr‐ and H_2_O‐treated mice. The white dashed line labels the outer and inner boundaries of the DG. Scale bar, 50 μm. Left, micrographs (10×) of the corresponding DG section, with nuclei stained with Hoechst 33258, in gray. Scale bar, 250 μm. (C) The total number of Ki67‐positive cells increased with HTyr treatment compared to H_2_O treatment in the whole DG (Treatment fixed effect: HTyr vs. H_2_O, *p < 0.05; Wald *χ* ^2^ test) and in the ventral DG (planned simple effects of Treatment: V‐DG **p = 0.01; Wald *χ* ^2^ test). (D) Proliferating type‐1‐2a progenitor cells (Ki67^+^/Sox2^+^) increased in the whole DG (Treatment fixed effect: *p < 0.05) and in the ventral DG (planned simple effects of Treatment: *p* = 0.01; Wald *χ* ^2^ test). (E) HTyr significantly amplified the number of type 2b‐3 cells (Ki67^+^/DCX^+^) in the whole DG (Treatment fixed effect: **p < 0.01) and in the ventral DG (planned simple effects of Treatment: *p < 0.05; Wald *χ* ^2^ test). In (C–E) sample size (*n*) consisted of 124 individual observations (DG sections) from 8 mice. The number of cells in the D‐DG and V‐DG was quantified in at least 24 sections of dentate gyri from four mice per experimental group. (F) The total number of DCX‐positive cells (neuroblasts and immature neurons) significantly increased in the whole DG (Treatment fixed effect: **p < 0.01; Wald *χ* ^2^ test) and in the ventral DG of HTyr mice (planned simple effects of Treatment: V‐DG **p < 0.01; Wald *χ* ^2^ test). Sample size (*n*) consisted of 115 individual observations (DG sections) from 8 mice. The number of cells in the D‐DG or V‐DG was quantified in at least 26 sections of dentate gyri from four mice per experimental group. (C–F) The numbers of dentate gyrus cells in the histograms are presented as means ± SEM. Statistical analysis was performed using a GLMM‐NB model with Mouse as random intercept. D‐DG, dorsal dentate gyrus; GLMM‐NB, generalized linear mixed model‐negative binomial; IHC, immunohistochemistry; V‐DG, ventral dentate gyrus.

**FIGURE 3:**
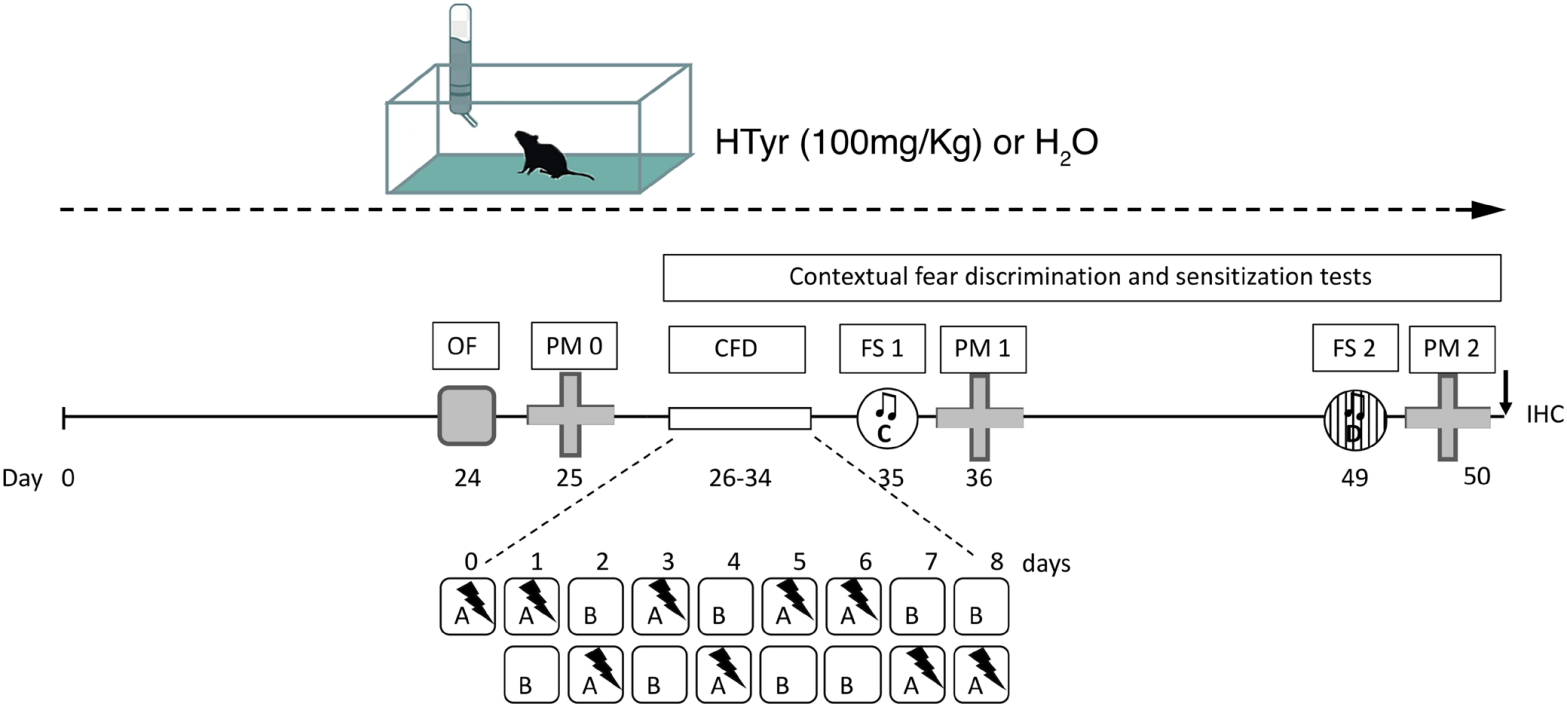
Experimental timeline of behavioral tests. Schematic representation of experimental plan. 15‐month‐old C57black6J mice were treated with standard water (H_2_O) or with HTyr 100 mg/kg for 50 days. At the indicated time points, mice were subjected to the behavioral tasks. CFD, Contextual Fear discrimination test; FS, Fear Sensitization test; OF, Open Field test; PM, Plus Maze test. Squares A and B: Context A and B; Circles C and D: Context C and D. See the text for details of the procedure.

Mice were administered with 100 mg/kg/day of HTyr (human equivalent dose 8.1 mg/kg/day; Nair and Jacob 2016) for 30 or 50 days as indicated in Figures 1A, 2A, 3, 7 and 8, according to our previous work (D'Andrea et al. 2020). The mouse weight was measured before and during the administration of HTyr. No differences in weight were observed between the two conditions. Mice were euthanized at the end of the treatment.

In mice treated with HTyr or water for 30 days, a daily intraperitoneal injection of 5‐Bromo‐2′‐deoxyuridine (BrdU, 95 mg/kg i.p.; Sigma Aldrich, St Louis, MO, USA) was administered for the first 5 days of treatment (see schematic representation in Figure 1A).

All procedures were performed on male mice to reduce variability associated with sex‐specific hormonal cycles and to ensure consistency with our previous studies (D'Andrea et al. 2020). All protocols were carried out in accordance with the current European (directive 2010/63/EU) Ethical Committee guidelines and the protocol of the Italian Ministry of Health (authorization 364‐2020‐PR).

### 2.2 Synthesis of HTyr

Pure HTyr was firstly synthetized by a three‐step patented procedure from 2‐hydroxyphenylethanol (tyrosol) (Bernini et al. 2008). More recently, the synthesis has been revised and simplified avoiding the protection and the following deprotection of the alcoholic group (Laghezza Masci et al. 2022). Tyrosol (Sigma Aldrich, St Louis, MO, USA; cat#188255) was directly oxidized with 2‐iodoxybenzoic acid [1‐hydroxy‐1‐oxo‐1H‐1λ5‐benz[d][1,2]iodoxol‐3‐one, IBX] (Sigma Aldrich, St Louis, MO, USA, cat#661384) previously prepared in methanol at *T* = −20°C for 2 h. The following reduction of the *ortho*‐quinone with sodium dithionite (Na_2_S_2_O_4_) in water at *T* = 25°C for 1 h afforded the catecholic compound (HTyr). The crude was purified by silica gel chromatographic column (eluent: dichloromethane/methanol: 9.8/0.2) affording HTyr (40% yield). The final product was characterized by nuclear magnetic resonance (^1^H and ^13^C NMR) to verify both the chemical structure and purity. Hydroxytyrosol was isolated as a 98% pure yellow oil.

### 2.3 Immunohistochemistry (BrdU Labeling, Immunofluorescence)

For immunohistochemistry, at the end of the experimental procedures, mice were deeply anesthetized by intraperitoneal injection of Tiletamine‐Zolazepam (1:1, 100 mg/kg) and Xylazine (20 mg/kg) followed by transcardiac perfusion with 4% paraformaldehyde (PFA) in PBS 1×. The collected brains were incubated overnight in 4% PFA, equilibrated in 30% sucrose, and cryopreserved at −80°C. The fixed brains embedded in Tissue‐Tek OCT (Sakura Finetek, Torrence, CA, USA) were sectioned into serial free‐floating coronal sections at 40 μm thickness at −25°C in a cryostat. Subsequently, the sections were processed for multiple labeling immunofluorescences. The sections were permeabilized with 0.3% Triton X‐100 in PBS and then incubated with primary antibodies with 3% normal donkey serum in 0.3% TritonX‐100 in PBS for 16–18 h at 4°C.

Proliferation was detected using rabbit monoclonal anti‐Ki67 (Biocare Medical, Pacheco, CA, USA; clone SP6, CRM325; RRID:AB_2721189; 1:100) in combination with either goat polyclonal antibody against Sox2 (Santa Cruz Biotechnology, Santa Cruz, CA, USA; RRID:AB_2286684; 1:300) or guinea pig anti‐doublecortin (DCX; Millipore; RRID:AB_1586992; 1:600) to label specific subpopulations of dentate gyrus stem and progenitor cells.

For BrdU staining, DNA was denatured with 2 N HCl for 45 min at 37°C, followed by 0.1 M sodium borate buffer pH 8.5 for 10 min and overnight incubation at 4°C with a rat monoclonal antibody against BrdU (Abcam, Cambridge, UK; cat#AB6326; RRID:AB_305426; 1:400) and a mouse monoclonal antibody anti‐NeuN (Millipore Burlington, MA, USA; cat#MAB377; RRID:AB_2298772; 1:400). The microglial population was labeled with the goat polyclonal antibody anti‐Iba1 (Abcam; cat#AB5076; RRID:AB_2224402; 1:600). The activated neurons were visualized with a rabbit polyclonal antibody against c‐fos (Millipore; Ab‐5 PC38; RRID:AB_2737414; 1:500) co‐labeled with NeuN.

Secondary antibodies were all obtained from Jackson ImmunoResearch (West Grove, PA, USA) and used as follows: a donkey anti‐rabbit antiserum Cy3‐conjugated (RRID:AB_2313568) (Ki67) or Cy2‐conjugated (RRID:AB_2340612) (c‐fos); a donkey anti‐rat antiserum TRITC‐conjugated (RRID:AB_2340635) (BrdU); a donkey anti‐goat conjugated to Alexa‐488 (RRID:AB_2340428) (Iba1) or Alexa‐647 (RRID:AB_2340437) (Sox2); a donkey anti‐mouse conjugated to Alexa‐647 (RRID:AB_2340862) (NeuN); a donkey anti‐guinea pig conjugated to Cy2 (RRID:AB_2340443) (DCX). Nuclei were counterstained by Hoechst 33258 (Sigma‐Aldrich, St Louis, MO, USA; Cat#H3570; 1 mg/mL in PBS).

To minimize auto‐fluorescence due to lipofuscin deposits, sections were treated with 0.3% Sudan Black (Sigma‐Aldrich; cat#199664) in 70% ethanol for 30 s and rinsed thoroughly with PBS 1× at the end of the procedures.

Confocal Z‐stacks and single‐plane images of the immunostained sections were obtained using a Leica TCS SP5 Broadband Confocal Laser Scanning Microscope (RRID:SCR_020233). Analyses were performed in sequential scanning mode to rule out cross‐bleeding between channels.

### 2.4 Quantification of Cell Numbers and Morphological Analysis

A stereological study of cell numbers was performed by analyzing one‐in‐eight 40‐μm free‐floating coronal sections (320 μm apart), using confocal microscopy. The sections were examined to count cells expressing the indicated marker across the whole rostrocaudal extent of the DG (longitudinal length of 2.56 mm).

The total estimated cell count was determined by multiplying the average number of positive cells per section by the total number of 40‐μm sections covering the entire dentate gyrus (approximately 64 for the whole DG, and approximately 32 for both ventral and dorsal DG). This method has been previously described (Farioli‐Vecchioli et al. 2012; Gould et al. 1999; Jessberger et al. 2005). A minimum of four animals per group was analyzed, as indicated in the figure legends. For each animal, about 8 sections (corresponding to 16 dentate gyrus sections) were examined. Cell number analyses were performed manually by trained experimenters, in a blinded fashion, using the image processing software Fiji (RRID: SCR_002285) to register positive cells.

For the morphological analysis of DCX‐positive cells in the DG, we employed the *Simple Neurite Tracer* (SNT; Arshadi et al. 2021) implemented in Fiji software (RRID: SCR_002285). Briefly, Z‐stack images of DCX‐positive cells were acquired in both dorsal and ventral DG using a 20× objective at 1.5 μm intervals for a total of 20 optical sections. Neurites were manually traced with the SNT plugin, and the resulting reconstructions were subjected to skeleton analysis to quantify total neurite length, number of branches, and junctions. In addition, Sholl analysis was performed (Ferreira et al. 2014) to assess the branching index of these cells, setting the first shell at 5 μm and the subsequent shell at a step size of 5 μm. At least 9 DCX^+^ cells/mouse and four mice per group were analyzed.

### 2.5 Microglia Analysis

For microglia density, 6 random Z‐stack acquisitions per mouse (20× objective, with 2 μm interval for a total of 17 steps) were made applying the same intensity and exposure time. A region of interest (ROI) encompassing the granular zone of the dentate gyrus was drawn. To calculate cellular density, the number of Iba1^+^ cells was manually counted and divided by the area covered by the ROI. The data are expressed as the number of cells per mm^2^.

For morphological analysis, we employed Sholl Analysis using a previously described method (McGill et al. 2018). Briefly, Z‐stack images (40× objective, digital zoom 1.5× with 1.13 μm interval for a total of 30 steps) were captured in random areas of the DG. From each image, using Fiji software (RRID: SCR_002285), single Iba1‐positive cells were isolated, and intensity threshold images (binary images) were generated for subsequent analysis. Initially, we manually measured the area of soma and branch endpoints. Then, the Sholl Analysis plug‐in (Ferreira et al. 2014) was utilized, with the first shell set at 5 μm and the subsequent shell at a step size of 2.5 μm, to determine the enclosing radius, primary branches, and ramification index. At least 25 Iba1^+^ cells/mouse and five mice per group were analyzed.

### 2.6 Behavioral Tests

#### 2.6.1 Open Field Test

HTyr‐treated and H_2_O‐treated (*n* = 19 per group) mice were placed in the center of a square (60 × 60 cm) arena and allowed to explore the apparatus for 10 min. After the first 5 min, an object (cylinder high 15 cm and wide 5 cm) was placed in the center of the apparatus, and mice were left in the arena for an additional 5 min. Behavior was videotaped by a video camera placed above the apparatus, and the time spent near the wall (outer zone), in the center of the apparatus (inner zone; 20 × 20 cm), and the distance moved in the arena were analyzed by using the ezTrack software (Copyright 2007 Free Software Foundation; RRID:SCR_021496) (Pennington et al. 2019).

#### 2.6.2 Elevated Plus Maze Test

The plus maze was carried out in a gray Plexiglas elevated maze with four arms 30‐cm long and 5‐cm wide, extending from a central starting platform. While two facing arms were closed by gray walls (15‐cm high), the other two arms were left open. The experimental room was lighted with two white‐tensor lamps (40 W). Animals were placed in the center of the apparatus and allowed to explore it for 5 min. Behavior was videotaped and the time spent in the open and closed arms, as well as the distance traveled, were analyzed using the ezTrack software (Copyrig(C) 2007 Free Software Foundation; RRID:SCR_021496). The plus maze tests were carried out one day before (PM0) and again two (PM1) and 16 days after (PM2) the contextual fear‐discrimination learning task.

#### 2.6.3 Contextual Fear Memory and Context Generalization Tasks (See Scheme in Figure S2)

The day after the assessment of basal behaviors, a subgroup of HTyr‐treated (*n* = 9) and H_2_O‐treated (*n* = 9) mice underwent a contextual fear conditioning task. Conditioning was carried out in training chamber A (Context A; 26 × 22 × 18 cm) made of transparent Plexiglas, with a steel grid as a floor, located in a sound‐insulated box and lighted from above by two white tensor lamps (30 W each). 180 s after placement of a mouse into the chamber, a footshock was delivered (2 s; 0.7 mA). Mice were left in the conditioning chamber for a further period of 20 s and then returned to their home cage. The apparatus was cleaned with a 30% v/v ethanol solution after mouse removal. Twenty‐four hours after training, contextual fear memory was tested by subjecting mice to the same Context A for 180 s without foot‐shock delivery. The following day a fear generalization test was carried out by exposing the mice to the new Context C (a cylindrical transparent box of 20 cm in diameter with a plastic gray floor; lighted with a red‐tensor lamp; fan was turned on; the apparatus was cleaned with a 70% v/v ethanol solution after mouse removal) for 180 s. Two weeks later, mice were exposed again to Context A for 180 s to test remote contextual fear memory retention. Twenty‐four hours later fear generalization was tested again in the new Context D (a cylindrical box of 20 cm in diameter with black and white strips; wood shavings on the floor; lighted with a green‐tensor lamp; fan was turned on; the apparatus was cleaned with a 70% v/v ethanol solution after mouse removal).

#### 2.6.4 Contextual Fear‐Discrimination Learning Task (CFD)

The day after the assessment of basal behaviors, a subgroup of HTyr‐treated (*n* = 10) and H_2_O‐treated (*n* = 10) mice underwent the contextual‐fear discrimination learning task (CFD) by using a procedure previously used in our laboratory (Farioli‐Vecchioli et al. 2014) to induce pattern separation in young‐adult mice. A further group of young (3‐month‐old) untreated mice was included as an additional control group (young‐untreat; *n* = 5).

In brief, conditioning was carried out in training chamber A (Context A) following the same procedure as for contextual fear memory task (180 s of context exposure; 2 s of footshock administration at 0.7 mA; 20 s of post‐shock exposure). Starting from the next day, the mice were exposed, on a daily basis, to both the training context (A), in which they continued to receive a single foot‐shock (2 s; 0.7 mA) 180 s after placement, and a similar context (B), in which they were placed about 1 h later and were left for 180 s with no foot‐shock administration (see scheme in Figure 3). The similar context differed from the shock‐associated context in the following features: two counter walls, made of black Plexiglas, were used to cover two of the four chamber walls; fan and noise were turned on; the sound‐isolated box which contained the training chamber was kept partially open; a lime scent was used as an olfactory cue; a 1% v/v acetic acid solution was used to clean the apparatus after mouse removal. Importantly, the two contexts shared the exposed steel grid as a floor (apart from many other features), which has been repeatedly shown to constitute a salient characteristic of the context, that is important for the early generalization of the fear memory. Behavior in the training chamber A and in the similar context B was videotaped. Freezing levels recorded during the first 3‐min were analyzed by using the ezTrack software (Copyright 2007 Free Software Foundation RRID:SCR_021496). The discrimination index obtained by calculating the ratio of the percentage of freezing displayed in training context A to the total amount of freezing displayed in contexts A and B was considered as a measure of pattern separation. Therefore, a discrimination index greater than 0.5 means that mice were able to discriminate between the similar contexts.

#### 2.6.5 Fear Sensitization Test (FS)

Previous studies have demonstrated that the contextual fear conditioning paradigm leads to the development of a non‐associative memory component—referred to as fear sensitization (Kamprath and Wotjak 2004; Siegmund and Wotjak 2007a, 2007b; Costanzi et al. 2011, 2014). To assess fear sensitization, mice were tested one day (FS1) and 15 days (FS2) after the contextual fear‐discrimination learning task. Animals (HTyr‐treated *n* = 10; H_2_O‐treated *n* = 10) were placed in new contexts in which the reaction to a neutral tone (Tone test; 80 dB, 9 kHz) was evaluated. The first fear sensitization test was carried out in the context C. The second fear sensitization test was carried out in the context D (see scheme in Figure 3). In both contexts, a neutral tone was continuously delivered during the last 3 min of a 6 min period. In parallel, a further group of “unshocked” aged mice treated with water (*n* = 10) was subjected to the same procedures and considered as a control group. Behavior was videotaped and freezing levels recorded during the tests were analyzed by using the ezTrack software (Copyright 2007 Free Software Foundation RRID:SCR_021496). The percentage of freezing recorded during the administration of the tone (the last 3 min of the test) was considered as an index of fear sensitization. All behavioral parameters were manually scored by an experimenter blind to the treatment of the subjects.

### 2.7 Statistical Analysis

For cell number quantification (Figures 1C,E, 2C–F, and 5B,C), the data were analyzed using Generalized Linear Mixed Models (GLMMs) with a Negative Binomial (NB) distribution to account for overdispersion. Because we performed counting of immunopositive cells in sections across the rostrocaudal extent of the dentate gyrus, GLMM analyses were performed on the raw counts per DG section. Fixed factors included Treatment (H_2_O vs. HTyr), Region (Dorsal vs. Ventral), and their interaction, while Mouse was included as a random intercept to account for repeated measures and inter‐individual variability. Models were fitted with a log link using Jamovi (GAMLj) (RRID:SCR_016142). Fixed effects were analyzed using Type III Wald chi‐square tests. Model quality was assessed through AIC, BIC, log‐likelihood, deviance, *R* ^2^ (marginal and conditional), and dispersion indices.

Given our specific a priori hypotheses regarding treatment effects within each region, we conducted planned simple‐effects analyses. These targeted comparisons evaluated the effect of treatment on the whole dentate gyrus (H_2_O vs. HTyr), the dorsal region (D‐H_2_O vs. D‐HTyr), and the ventral region (V‐H_2_O vs. V‐HTyr).

For cell counts/area (Figure 6B) and morphological analysis of Iba1‐ and DCX‐positive cells (Figure 6D, Figure S1), because values were continuous, approximately Gaussian, and showed no evidence of overdispersion, we used a Linear mixed Model (LMM). Treatment (H_2_O vs. HTyr) was included as a fixed factor, and Mouse was modeled as a random intercept to account for repeated measures and inter‐individual variability. Fixed effects were evaluated using Type III Wald *F*‐tests with Satterthwaite approximation for denominator degrees of freedom. Where indicated, planned simple‐effects analyses were performed to evaluate the effect of treatment on each region. Assumptions of the LMM were assessed through inspection of residual diagnostics.

Differences were considered statistically significant at *p* < 0.05. The number of mice analyzed per group is indicated in the figure legends (at least 4/group) and was defined on the basis of our previous studies (D'Andrea et al. 2020).

**FIGURE 4:**
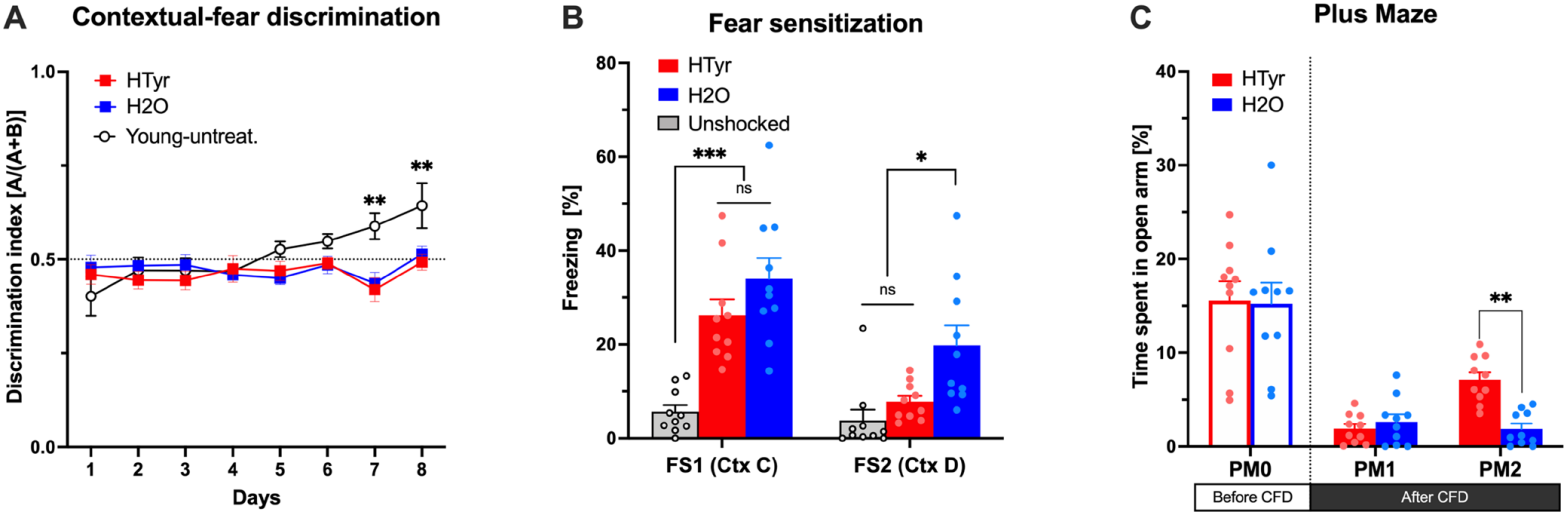
HTyr reduces anxiety‐like behaviors after a traumatic experience (see text for details of the procedure and the scheme in Figure 3). (A) Discrimination index, calculated as the ratio of freezing levels displayed in training context A (paired with shock) to the total amount of freezing displayed in contexts A and B (without shock administration) during each daily session of the fear discrimination learning task. Data are shown for HTyr (*n* = 10) and H_2_O (*n* = 10) 15‐month‐old mice, and young‐untreated control mice (*n* = 5). (B) Percentage of freezing recorded in response to a neutral tone administration (fear sensitization) by HTyr, H_2_O and unshocked naïve (*n* = 10, white bars) 15‐month‐old mice in the contexts C and D, immediately (FS1) and 15 days (FS2) after the contextual fear discrimination learning task. (C) Percentage of time spent in the open arm of the plus maze by HTyr and H_2_O mice before (PM0, open bars) and after (PM1, PM2) the contextual fear discrimination learning task. All the data are shown as mean ± SEM. Statistical significance was assessed by two‐way (A, C) or one‐way ANOVA (B), followed by Tukey's post hoc test for multiple comparisons between groups (*p < 0.05; **p < 0.01; NS *p* > 0.05). FS, Fear Sensitization test; PM, Plus Maze test.

Data obtained from the behavioral experiments were first assessed for normality using the Shapiro–Wilk test. Based on the results (*p* > 0.05), comparisons between HTyr‐treated and H_2_O‐treated mice were performed using the two‐tailed Student's *t*‐test. One‐way ANOVA was used when more than two groups were considered (as in the case of fear sensitization tests in which an additional group of unshocked mice was tested, see Figure 4). Two‐way ANOVA was used when the effect of either training or time was considered as repeated measures. Individual between‐group comparisons, where appropriate, were carried out by Tukey's post hoc test. Statistical significance was accepted if *p* < 0.05. The sample size was determined by a priori Power analysis using the G*Power software (Heinrich Heine, Düsseldorf University; RRID:SCR_013726) (Test family: *t*‐test; statistical test = means: difference between two independent means‐two groups; two‐tails, effect size = 1.8; *p* = 0.05, Power = 0.95). All the data are expressed as mean values ± SEM. For statistical analyses relating to microbiota studies, please refer to the relevant Methods section.

### 2.8 Fecal Sample Collection and DNA Extraction

Fecal samples were collected from H_2_O‐ and HTyr‐treated mice non subjected to behavioral tests at the initial (Ti) and final (Tf) time points of treatment (see timeline in Figure 7A; samples/H_2_O group: 9; samples/HTyr group: 10) and from H_2_O‐ and HTyr‐treated mice subjected to behavioral tests at Ti and Tf (see timeline in Figure 8A; samples/H_2_O group: 10; samples/HTyr group: 10). Fecal sample collection was carried out by placing mice separately in clean cages without bedding. Fresh fecal pellets were collected using a single mouse pick and promptly transferred into vials placed immediately in dry ice to preserve their integrity. After collection, the samples were stored at −80°C until further processing. DNA extraction from fecal pellets was performed using the QIAamp DNA Stool Mini kit (Qiagen, Hilden, Germany, Cat#51604), following the manufacturer's protocol. Before DNA extraction, 100 mg of stool per sample were homogenized using the Precellys Evolution Touch homogenizer (Bertin Technologies, Montigny‐le‐Bretonneux, France, RRID:SCR_022979). DNA concentration was determined using a Qubit 4 Fluorometer (RRID:SCR_026883) with the dsDNA HS Assay Kit (ThermoFisher Scientific, Waltham, MA, Cat# 32851). Absorption ratios 260/280 and 260/230 were used to estimate the quality and purity of the extracted DNA. The absence of PCR inhibitors was confirmed by amplifying the V3–V4 region of the 16S rRNA gene with the primer pairs 341F (5′‐CCTACGGGNGGCWGCAG‐3′) and 805R (5′‐GACTACHVGGGTATCTAATCC‐3′) (Herlemann et al. 2011).

### 2.9 16S rRNA Gene Sequence and Bioinformatic Analysis

Libraries were constructed following the Illumina 16S Metagenomic Sequencing Library Preparation protocol, involving two amplification steps: an initial PCR amplification using locus‐specific PCR primers and a subsequent amplification that incorporates relevant flow‐cell binding domains and unique indices (Nextera XT Index Kit, FC‐131‐1001/FC‐131‐1002) (Illumina, San Diego, CA, USA). The V3‐V4 regions of the 16S ribosomal RNA gene were targeted for amplification using Illumina‐tailed primers: Pro341F (5′‐CCTACGGGNBGCASCAG‐3′) and Pro805R (5′‐GACTACNVGGGTATCTAATCC‐3′); the sequences reported in the brackets refer only to PCR primer regions. Sequencing was performed on an Illumina MiSeq instrument (RRID:SCR_016379) (Illumina, San Diego, CA, USA) in paired‐end 300‐bp mode read length.

Demultiplexed sequences were processed with the software Amplicon ToolKit (AMPtk) for NGS data (formally UFITS) v.1.3.0 (Palmer et al. 2018). Starting reads were quality trimmed and PhiX screened using USEARCH v. 11.0.667 (Edgar 2010). Reads with less than 100 bp were removed, longer reads were trimmed to the length of 280 bp, and paired‐end reads were merged in one step. Obtained reads were clustered in Amplicon Sequence Variants (ASVs), using DADA2 v1.20.0 (RRID:SCR_023519) (Callahan et al. 2016), which includes phiX reads removal and chimera detection. To reduce noise, all singleton ASVs were removed from the dataset. Taxonomy was assigned to ASVs using the RDP11 database (Cole et al. 2014) through a hybrid approach that calculates the local common ancestor based on the results of a Global Alignment (USEARCH/VSEARCH), UTAX, and SINTAX (Edgar 2010). ASVs that had no match in the database, showed less than 70% to any bacterial taxa, or were identified as chloroplasts were excluded from the analysis.

The subsequent analyses were performed with the R software (v 4.4.2) (RRID:SCR_001905). The alpha‐diversity Richness, Shannon, Simpson, and Evenness indices were calculated for each sample. The data were not assessed for normality because metabarcoding datasets are compositional and typically non‐normally distributed.

Significant differences in alpha‐diversity indices, Firmicutes/Bacteroidetes ratio, and taxa abundances across treatments were assessed using the Kruskal–Wallis test. Planned pairwise comparisons between treatment pairs (as indicated in Figure 8 and Figures S5 and S10) of taxa were analyzed with the two‐tailed Mann–Whitney *U* test when appropriate. Beta diversity analyses were performed on Bray‐Curtis distance matrices derived from Hellinger‐transformed ASV abundances and represented through NMDS (non‐metric multidimensional scaling) ordination. Differences in community composition across treatment were evaluated using PERMANOVA.

To identify genera with the most significant effect sizes between groups, differential abundance analysis was performed using the linear discriminant analysis (LDA) effect size (LefSe) approach (Segata et al. 2011), as implemented in the R package *microbiomeMarker*. The analysis was conducted on normalized genera relative abundance data. For each taxon, a non‐parametric Kruskal–Wallis test was first applied to detect features showing significant differences across groups. Features passing this statistical test were subsequently subjected to linear discriminant analysis (LDA) to estimate the effect size and identify taxa most strongly associated with each group. An LDA score threshold of 2.0 and a significance level of *p* < 0.05 were applied.

## 3 Results

We previously demonstrated that treatment with HTyr can counteract the neurogenic deficit that occurs in the dentate gyrus of aged mice. Here, we intended to investigate the behavioral consequences of the pro‐neurogenic action of HTyr and the potential contribution of the gut microbiota. To this aim, 15‐month‐old mice were administered HTyr in drinking water (100 mg/kg/day based on prior studies; D'Andrea et al. 2020) for 30 or 50 days and compared with age‐matched mice given standard water (control H_2_O mice).

All experiments utilized HTyr synthesized in our laboratories according to a patented procedure (Bernini et al. 2008) recently modified (Laghezza Masci et al. 2022).

### 3.1 HTyr Promotes the Survival of Newly Formed Neurons in the Dorsal and Ventral Dentate Gyrus

The granule cells of the hippocampal dentate gyrus (DG) present functions topographically distributed along the dorsoventral axis (Kjelstrup et al. 2002; Bannerman et al. 2004). To assess the functional effects of HTyr treatment, we first analyzed the dorsoventral distribution of newly generated neurons in the dentate gyrus of 15‐month‐old mice treated for 30 days with HTyr (HTyr mice) or with standard water (H_2_O mice). Moreover, mice received BrdU injections (i.p., 95 mg/kg) during the first 5 days of treatment to allow the neuron birth‐dating, following the experimental paradigm outlined in Figure 1A. This protocol enabled us to evaluate the pro‐survival and pro‐differentiative effects of HTyr on 26‐ to 30‐day‐old new neurons, previously observed in the whole DG (D'Andrea et al. 2020), in the two portions of the DG. Coronal sections of the whole DG (W‐DG) were divided into dorsal (D) and ventral (V) regions along the longitudinal (dorsoventral) axis. The number of BrdU‐positive cells co‐labeled with the neural marker NeuN (stage 6 neurons; Steiner et al. 2006) was quantified. A generalized linear mixed model with negative binomial distribution (GLMM, NB) revealed a significant main effect of treatment, as expected (BrdU^+^/NeuN^+^ cells: W‐DG HTyr vs. H_2_O, 2.1‐fold increase, *χ* ^2^(1) = 18.38, *p* < 0.001), while Region and the Region × treatment interaction were not significant. Planned simple effects analysis showed that HTyr increased the number of BrdU^+^/NeuN^+^ newly formed neurons in both regions (D‐DG: 2‐fold increase, *χ* ^2^(1) = 11.47, *p* < 0.001; V‐DG: 2 fold increase, *χ* ^2^(1) = 7.10, *p* = 0.008), as shown in Figure 1B,C.

We next examined the population of DCX‐expressing cells (type‐2b/3 neuroblasts and stage 5 immature granule cells; Kronenberg et al. 2003; Brandt et al. 2003). GLMM‐NB model revealed a significant main effect of Treatment (*χ* ^2^(1) = 6.97, *p* = 0.008), whereas Region and the Region × Treatment interaction were not significant. Planned simple‐effects analyses showed that HTyr increased the number of DCX^+^ cells in the whole DG (33% increase) and in the ventral DG (43% increase, *χ* ^2^(1) = 4.93, *p* = 0.026). Conversely, in the dorsal DG, we observed a non‐significant increase of DCX‐positive cells (Figure 1D,E).

### 3.2 Pro‐Neurogenic Effect of an Extended Treatment With HTyr in the Dorsoventral Dentate Gyrus

The observed increase of newly formed neurons in both the dorsal and ventral DG of HTyr‐treated aged mice prompted an investigation into its impact on behavioral abilities specifically related to these two regions. To design an ad hoc experimental paradigm, we first examined whether an extension of treatment with HTyr to 50 days maintained its effectiveness in promoting neurogenesis, as seen with the 30‐day treatment (Figure 2A). To assess this, we quantified stem and progenitor cells in the DG of mice treated with HTyr or H_2_O for 50 days by counting the cells expressing the proliferation marker Ki67 and co‐expressing the markers Sox2 or DCX, in order to distinguish the proliferating stem/progenitor cells (type‐1/‐2a, Ki67^+^/Sox2^+^; Komitova and Eriksson 2004; Steiner et al. 2006) and progenitor/neuroblast cells (type‐2b/‐3, Ki67^+^/DCX^+^; Kronenberg et al. 2003) (Figure 2B–E).

We observed that the extended treatment with HTyr had a pro‐proliferative effect on neural stem and progenitor cells in the whole DG (W‐DG), as revealed by GLMM‐NB. HTyr induced a significant main effect of Treatment for all three populations (HTyr vs. H_2_O, Ki67^+^: 2‐fold increase, *χ* ^2^(1) = 5.237, *p* = 0.022; Ki67^+^/Sox2^+^: 93% increase, *χ* ^2^(1) = 4.235, *p* = 0.04; Ki67^+^/DCX^+^: 2.5‐fold increase, *χ* ^2^(1) = 6.7705, *p* = 0.009), whereas Region and the Region × Treatment interaction were not significant. Planned simple‐effects analyses showed that this proliferative effect was region‐specific, occurring significantly in the ventral DG but not in the dorsal DG (Figure 2C–E) (V‐DG, HTyr vs. H_2_O, Ki67^+^cells: 2.7‐fold increase, *χ* ^2^(1) = 6.71, *p* = 0.01; Ki67^+^/Sox2^+^ cells: 3.5‐fold increase, *χ* ^2^(1) = 6.647, *p* = 0.01; Ki67^+^/DCX^+^ cells: 2.7‐fold increase, *χ* ^2^(1) = 4.11, *p* = 0.043). Moreover, a significant increase of total DCX‐positive neuroblast and immature granule cells was observed in HTyr‐administered mice compared to water‐drinking mice (Figure 2F). A GLMM‐NB revealed a significant main effect of Treatment in the whole DG (*χ* ^2^(1) = 8.595, *p* = 0.003), corresponding to a ~1.7‐fold increase. Planned simple effects showed a significant increase in the ventral DG (~1.9‐fold, *χ* ^2^(1) = 8.28, *p* = 0.004), whereas the increase in the dorsal DG (~1.5‐fold) did not reach significance (*χ* ^2^(1) = 3.3, *p* = 0.069; Figure 2F).

Finally, to assess whether HTyr supplementation might influence the morphology of newly generated DCX^+^ immature neurons, we performed analyses focusing on dendritic length, branching, and junction number, and branching index. In HTyr‐treated mice, we observed a significant increase in both the number of dendritic branches and junctions compared to H_2_O‐treated controls, specifically in the ventral region, indicating an increase of arborization (Figure S1). Notably, total dendritic length and branching index remained unchanged. These findings suggest that HTyr treatment may contribute to increased dendritic complexity during neuroblast maturation.

Overall, 50 days of HTyr treatment boosted neurogenesis in both the dorsal and ventral regions of the DG in aged mice, though stem/progenitor cell proliferation resulted significantly induced only in the ventral DG. Based on these findings, we then subjected the mice to the behavioral tests described below during a 50‐day treatment period with HTyr or H_2_O.

### 3.3 HTyr Treatment Does Not Affect Basal Behaviors and Anxiety‐Like Levels in Aged Mice

As an initial step toward a comprehensive behavioral evaluation, the effect of HTyr on basal behaviors was assessed in 15‐month‐old mice both by Open Field (OF) and Plus Maze (PM) tests, conducted after 24 and 25 days, respectively, of treatment with HTyr (*n* = 19) or with H_2_O (*n* = 19). These behaviors were assessed in order to rule out baseline anxiety‐like behavior and exploration differences between groups, which could confound results from following cognitive tasks.

In the OF test, all animals exhibited comparable levels of anxiety and exploration as measured by the percentage of time spent in the inner sector of the open field with (HTyr: 16.25 ± 2.29, H_2_O: 13.05 ± 1.89; *t*_36_ = 1.07; *p* = NS) or without (HTyr: 7.59 ± 3.04, H_2_O: 5.82 ± 1.43; *t*_36_ = 0.53; *P* = NS) the presence of an object in the center of the arena, and by the total distance (cm) traveled in the apparatus (HTyr: 6113.39 ± 233.31, H_2_O: 6254.96 ± 244.76; *t*_36_ = 0.42; *p* = NS). Basal anxiety was further tested in the PM test. No significant differences between groups were observed in the percentage of time spent in the open arms (HTyr: 11.88 ± 1.79, H_2_O: 15.48 ± 2.85; *t*_36_ = 1.069; *p* = NS) or in the distance (cm) traveled in the apparatus (HTyr: 1049.48 ± 55.79, H_2_O: 998.92 ± 38.28; *t*_36_ = 0.75; *p* = NS).

Together, these results indicated that HTyr treatment did not affect the baseline anxiety and exploration activity of aged mice.

### 3.4 HTyr Supplementation Does Not Influence Contextual Fear Memory and Context Generalization in Aged Mice

To examine the effect of HTyr on the cognitive abilities of aged mice, a subgroup of HTyr‐treated (*n* = 9) and H_2_O‐treated (*n* = 9) animals, previously tested in both the OF and PM, were tested in a one‐shock contextual fear conditioning task (experimental timeline shown in Figure S2A).

All animals acquired conditioning to a foot shock in Context A (Ctx A) delivered on day 33 after the initiation of treatment, as shown by freezing levels upon re‐exposure to the same context both 1 and 16 days later (training vs. tests: *F*_2,32_ = 44.69; *p* < 0.0001). No differences between groups were observed in the contextual memory tests (HTyr vs. H_2_O: *F*_1,16_ = 2.531; *p* = NS) (Figure S2B).

The day following the contextual memory tests, both the HTyr and H_2_O groups were exposed to novel no‐shock contexts (Context C and Context D, 2 and 17 days after training, respectively) to test their ability to distinguish between decidedly different contexts (context generalization). No differences between the HTyr and H_2_O mice were observed in both Context C (*t*_16_ = 0.4550; *p* = NS) and Context D (*t*_16_ = 1.748; *p* = NS) (Figure S2C).

These results clearly indicated that HTyr did not affect either recent (1 day) or remote (16 days) contextual fear memory formation, as well as the ability to distinguish between decidedly different contexts.

### 3.5 HTyr Treatment Reduces the Development of Fear Sensitization and Anxiety‐Related Behavior After a Frightening Experience but Does Not Ameliorate the Contextual Fear Discrimination Deficit of Aged Mice

Since adult neurogenesis occurring in the dorsal region of the DG is known to contribute to the “pattern separation,” which, at the behavioral level, corresponds to the ability to discriminate among potentially overlapping experiences (França et al. 2017; Anacker and Hen 2017; Fölsz et al. 2023), we hypothesized that the increase in adult neurogenesis observed in the dorsal DG (Figure 2) of aged mice treated with HTyr may lead to improved pattern separation. Therefore, 15‐month‐old HTyr‐treated (*n* = 10) and H_2_O‐treated (*n* = 10) mice were submitted to a contextual‐fear discrimination task (CFD) (McHugh et al. 2007), following a procedure previously used in our laboratory with minor modifications (Farioli‐Vecchioli et al. 2014) and described in experimental timeline (Figure 3). A further group of young (3‐month‐old) untreated mice was included as an additional control group (Young‐untreat; *n* = 5). Mice underwent the CFD task for 8 consecutive days, in which they learned to discriminate the context where the unconditioned stimulus (footshock) was administered (context A) from a very similar context where the unconditioned stimulus was never delivered (context B).

A two‐way ANOVA performed on the discrimination index revealed a significant effect of both training (*F*_7,154_ = 4.23, *p =* 0.0003) and training × group interaction (*F*_14,154_ = 2.36, *p =* 0.0053), but not a significant effect of group (*F*_2,22_ = 3.28, *p =* NS). Post hoc comparisons (Tukey's *post hoc* test) revealed a significant difference (*p* < 0.01) at both day 7 and day 8 between the untreated young mice and the HTyr‐ and H_2_O‐treated old mice, which did not differ (Figure 4A).

Apart from contextual discrimination, it is known that fear conditioning mimics post‐traumatic stress disorder (PTSD) in mice and induces the development of anxiety‐like behaviors (Siegmund and Wotjak 2006; Maeng and Milad 2017).

Given that the ventral hippocampus is widely recognized to exert a control role of anxiety (Bannerman et al. 2004; Kheirbek et al. 2013; Hong and Kaang 2022), the increase in adult neurogenesis observed in the ventral DG of HTyr‐treated mice (Figures 1 and 2) led us to hypothesize a possible beneficial role of HTyr on the development of posttraumatic anxiety. To assess this hypothesis, HTyr‐treated and H_2_O‐treated mice were subjected to fear sensitization tests either 1 (FS1) or 15 (FS2) days after the frightening experience caused by the CFD task. During these tests, mice were exposed to novel no‐shock contexts (Context C and Context D, respectively), and their reaction to a neutral tone was evaluated (Figures 3 and 4B). A further group of unshocked and untreated aged mice (*n* = 10) was included as a control. Statistical analyses (one‐way ANOVAs) on freezing to tone showed significant differences among groups at both FS1 (*F*_2,27_ = 19.71; *p* < 0.001) and FS2 (*F*_2,27_ = 8.37; *p* = 0.001) tests. Post hoc analyses (Tukey's post hoc test) revealed that at FS1 both H_2_O‐ and HTyr‐treated mice froze significantly (*p* < 0.001) more than unshocked mice. However, in the FS2 test, H_2_O‐treated mice froze significantly (*p* < 0.02) more than both HTyr‐treated and unshocked mice, which were indistinguishable from each other (Figure 4B). These results indicated a reduction of fear sensitization in HTyr‐treated but not in H_2_O‐treated mice, suggesting that hydroxytyrosol supplementation is able to prevent fear incubation.

To test the levels of posttraumatic anxiety‐like behavior after fear conditioning, HTyr‐treated and H_2_O‐treated mice were re‐submitted to the elevated plus maze task after the fear sensitization tests (see Figure 3). We compared the percentage of time spent in the open arms by HTyr‐treated and H_2_O‐treated mice before (PM0) and after the frightening experience in the CFD task (PM1 and PM2) (Figure 4C). Two‐way ANOVA analysis showed a significant effect of time (*F*_2,36_ = 63.44; *p* < 0.0001), a significant effect of the treatment × time interaction (*F*_2,36_ = 3.261; *p* = 0.05) and no significant effect of treatment (*F*_1,18_ = 1.568; *p* = NS). Post hoc comparisons (Tukey's post hoc test) revealed that the percentage of time that HTyr‐treated and H_2_O‐treated mice spent in the open arms before the frightening experience was significantly higher (*p* < 0.0001) than that spent by the mice at PM1 after fear conditioning (Figure 4C). This result suggests that both groups of mice suffered from posttraumatic anxiety in the aftermath of the traumatic experience. However, at PM2, approximately 2 weeks after the frightening experience, HTyr‐treated mice spent significantly (*p* < 0.01) more time in the open arms than H_2_O‐treated mice (Figure 4C), suggesting that the HTyr‐supplemented diet ameliorates the effect of fear incubation on posttraumatic anxiety‐like behavior of aged mice.

### 3.6 HTyr Stimulates the Activation of Granule Neurons in Both the Dorsal and Ventral DG After the Behavioral Tasks

**FIGURE 5:**
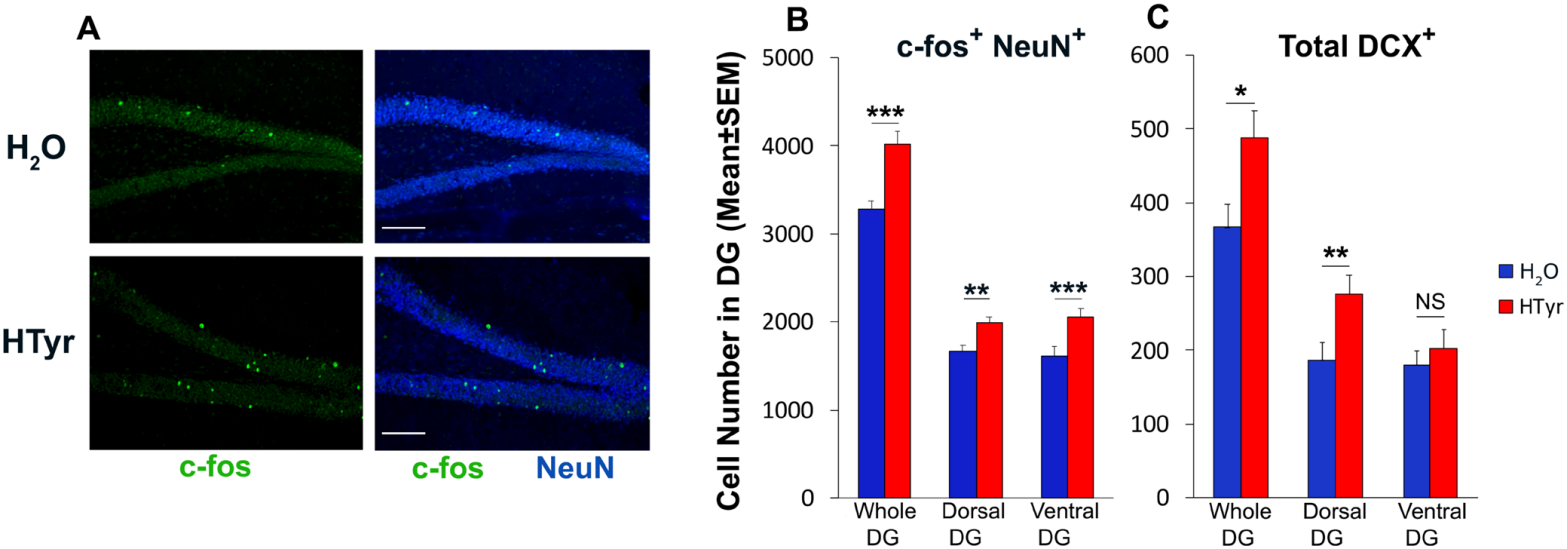
HTyr stimulates neuron activation in dorsal and ventral DG in a mouse model of PTSD. The dentate gyri of mice subjected to behavioral tests, as described in Figure 3, were analyzed for the expression of the c‐fos marker of active neurons. (A) Representative confocal images (20×) of neurons positive for c‐fos (green) and NeuN (blue). Scale bar, 100 μm. (B) The number of activated neurons (c‐fos^+^/NeuN^+^) was increased by treatment with HTyr in the whole DG (fixed effect: Treatment ***p < 0.001; Wald *χ* ^2^ tests) and in the ventral and dorsal DG (planned simple effects of Treatment: D‐DG **p = 0.01; V‐DG ***p < 0.001, Type III Wald *χ* ^2^ test). Sample size (*n*) consisted of 150 individual observations (DG sections) from 10 mice. The number of cells in the D‐DG or V‐DG was quantified in at least 36 sections of dentate gyri from five mice per experimental group. (C) The total number of DCX‐positive cells (neuroblasts and immature neurons) was significantly increased in the whole (fixed effect: Treatment *p < 0.05; Wald *χ* ^2^ test) and dorsal DG of HTyr mice (planned simple effects of Treatment: D‐DG **p = 0.01). Sample size consisted of 154 individual observations (DG sections) from 10 mice The number of cells in the D‐DG or V‐DG was quantified in at least 35 sections of dentate gyri from five mice per experimental group. The cell counts shown in the histograms are presented as means ± SEM. Statistical analysis was performed using a GLMM‐NB model with Mouse as random intercept. D‐DG, dorsal dentate gyrus; IHC, immunohistochemistry; GLMM‐NB, generalized linear mixed model‐negative binomial; V‐DG, ventral dentate gyrus.

At 1.5 h following the last elevated plus maze test (PM2, see Figure 3), HTyr‐ and H_2_O‐treated mice were sacrificed to analyze the activation status of granular neurons in the dentate gyrus. This was assessed by quantifying c‐fos‐positive cells. An increase in the expression of the immediate early gene c‐fos is thought to identify neurons that have recently undergone activity and is used as a molecular tool to define active cell populations induced after a stimulus. A GLMM‐NB revealed a significant main effect of Treatment (*χ* ^2^(1) = 18.42, *p* < 0.001), whereas Region and the Region × Treatment interaction were not significant. As shown in Figure 5A,B, a significant increase in the number of granule cells expressing the c‐fos protein was observed in the whole DG of HTyr‐treated compared with H_2_O‐treated mice (1.23‐fold increase), and such increase was irrespective of the position along the dorsoventral axis as confirmed by planned simple‐effects analyses (D‐DG, 1.19‐fold increase, *χ* ^2^(1) = 6.66, *p* = 0.01; V‐DG, 1.27‐fold increase, *χ* ^2^(1) = 13.66, *p* < 0.001). Interestingly, a positive effect of HTyr treatment on the population of neuroblasts and immature neurons persisted after the behavioral tests (Figure 5C). A GLMM‐NB revealed a significant main effect of Treatment (*χ* ^2^(1) = 5.15, *p* = 0.023), whereas Region and the Region × Treatment interaction were not significant. HTyr increased the number of DCX^+^ cells in the whole DG (1.30‐fold increase). Planned simple‐effects analyses showed that this effect was driven by a significant increase in the dorsal DG (1.47‐fold increase, *χ* ^2^(1) = 6.548, *p* = 0.01), whereas the modest increase observed in the ventral DG (1.12‐fold increase) did not reach significance (Figure 5C).

### 3.7 HTyr Treatment Reduces Microglia Activation in Stressed Mice

In a previous study, we observed a reduction in the microglial population in aged mice following a 30‐day treatment with HTyr (D'Andrea et al. 2020). Here, we sought to evaluate whether the resilience to stress, observed in behavioral tests, is correlated with an effect of HTyr on the inflammatory state in the hippocampus.

**FIGURE 6:**
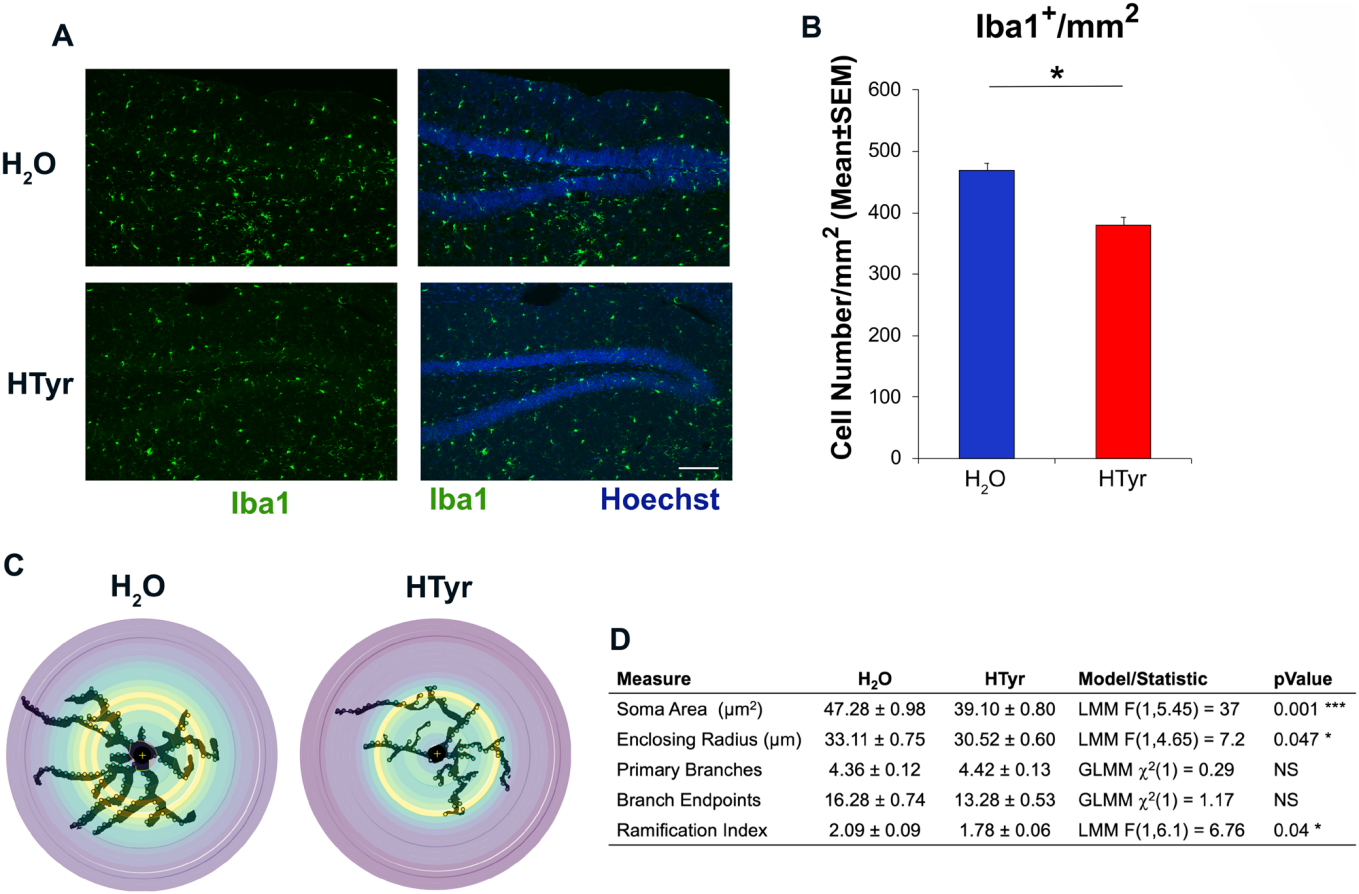
HTyr reduces the microglia activation in the DG of stressed mice. The dentate gyri of mice, treated as described in Figure 3, were analyzed for the expression of the Iba1 marker of activated microglia. (A) Confocal images (20×) representative of Iba1‐positive cells (in green) in dentate gyrus of treated (HTyr) and untreated (H_2_O) mice. In blue, nuclei counterstained with Hoechst 33258. Scale bar, 100 μm. (B) Density of microglia cells in dentate gyrus was reduced by treatment with HTyr. The number of cells/mm^2^ is presented as mean ± SEM. Each group included 5 mice with 30 sections of dentate gyrus quantified per group. Statistical test: LMM with random intercept for Mouse *p < 0.05, Wald *F*‐test. (C) Example of single microglial cells (Iba1‐positive) from the dentate gyrus of treated (HTyr) and untreated (H_2_O) mice, with concentric Sholl Radii (colorful circles) and points of intersection (colorful dots). The results of Sholl Analysis, summarized in table (D), showed a decrease in the soma area, enclosing radius, and ramification index. Sample size consisted of 312 individual observations (Iba‐positive cells) from 10 mice. Each group consisted of 5 mice. Data are means ± SEM. At least, 25 Iba1^+^ cells/mouse were analyzed by Sholl analysis. Statistical test: Continuous outcomes were analyzed using LMM with a random intercept for Mouse; fixed‐effect significance was assessed using Wald *F*‐tests; count outcomes were analyzed using GLMM‐NB with a random intercept for Mouse; significance was assessed using Wald *χ* ^2^ test. Simple effects of treatment were evaluated within each model. *p < 0.05, ***p < 0.001, NS *p* > 0.05. GLMM‐NB, generalized linear mixed model‐negative binomial; LMM, linear mixed model.

To this aim, we identified activated microglia by labeling cells with the specific marker Iba1, and we observed a significant reduction (18.5% decrease) of the number of Iba1‐positive cells/mm^2^ in the dentate gyrus of HTyr‐treated mice subjected to behavioral testing (Figure 6A,B). This effect was confirmed by a Linear Mixed Model (LMM; fixed effect of Treatment: *F*_1,8_ = 7.36, *p* = 0.027), suggesting an anti‐inflammatory action of HTyr. As expected, behavioral testing produced changes in the morphology of microglia in untreated mice (H_2_O), particularly an enlargement in the soma area (as observed comparing stressed versus unstressed H_2_O‐treated mice; data not shown), which is indicative of a transition to an activated phenotype (McGill et al. 2018). The activation of microglia is also known to be characterized by hyper‐ramification (He et al. 2024). Interestingly, in stressed mice treated with HTyr, we observed a reduction in the activated microglial cell soma area and ramification index, relative to those in H_2_O‐treated mice (Figure 6C,D), associated with a significant reduction in enclosing radius and a non‐significant reduction in branch endpoints, suggesting a possible attenuation of the inflammatory status in the dentate gyrus due to HTyr treatment.

### 3.8 HTyr Administration Favors the Stability of Beneficial Bacteria in the Gut of Mice Under Basal and Stressed Conditions

**FIGURE 7:**
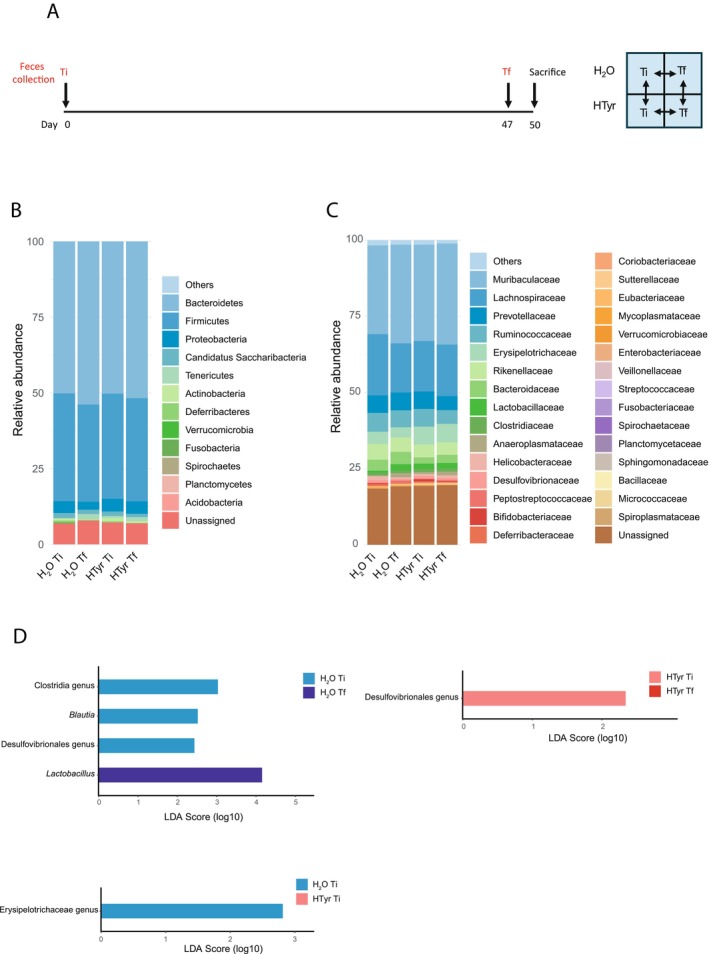
Effect of HTyr administration on the gut microbial composition of unstressed mice. (A) Timeline of fecal sampling in BT− mice (left) and outline of the comparisons carried out (right); Ti, initial time point; Tf, final time point; (B) Reads distribution at the phylum level (relative abundance); (C) Reads distribution at the family level (relative abundance). The top 30 most abundant families are shown; (D) LEfSe barplots showing differently enriched bacteria among groups (LDA score > 2.0, *p* < 0.05). Light blue bars: Genera enriched in H_2_O Ti vs. H_2_O Tf (up, left) and H_2_O Ti vs. HTyr Ti (down, left); blue bars: Genera enriched in H_2_O Tf vs. H_2_O Ti (up, left); light red bars: Genera enriched in HTyr Ti vs. HTyr Tf (up, right).

**FIGURE 8:**
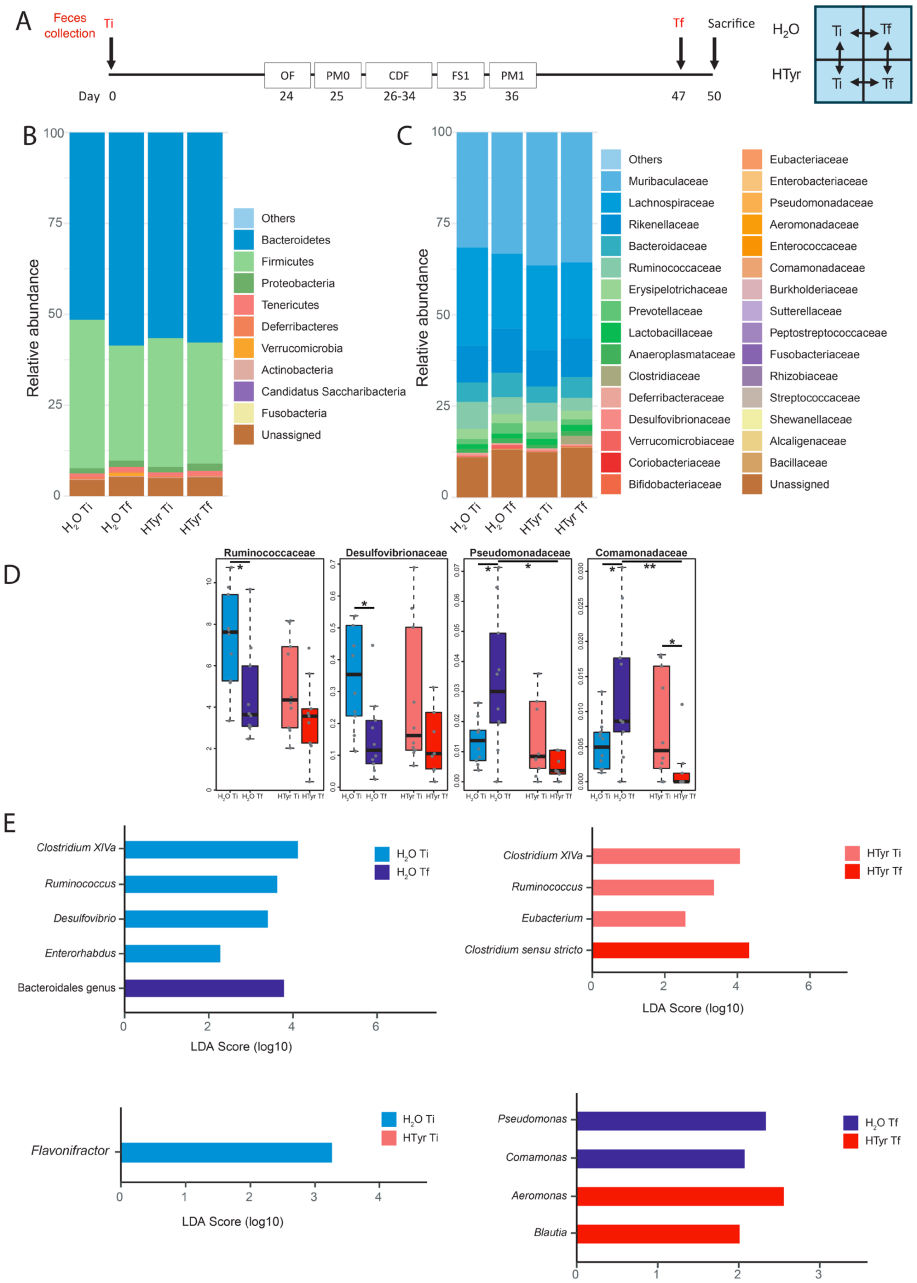
Effects of HTyr administration on the gut microbial composition in the presence of a traumatic experience. (A) Timeline of fecal sampling in BT+ mice (left) and outline of the comparisons carried out (right); Ti, initial time point; Tf, final time point. The abbreviations in the boxes are described in Figure 3; (B) Reads distribution at the phylum level (relative abundance); (C) Reads distribution at the family level (relative abundance). The top 30 most abundant families are shown; (D) Relative abundance of significantly different families in BT+ mice discussed in the text. Ruminococcaceae: Kruskal–Wallis [df = 3; *H* = 10.693] *p* = 0.01351; Mann–Whitney *U* test H_2_O Ti vs. H_2_O Tf: *U* = 27, *p* = 0.01726. Desulfovibrionaceae: Kruskal–Wallis [df = 3; *H* = 8.1937] *p* = 0.04217; Mann–Whitney *U* test H_2_O Ti vs. H_2_O Tf: *U* = 29, *p* = 0.01133. Pseudomonadaceae: Kruskal–Wallis [df = 3; *H* = 8.1517] *p* = 0.04298; Mann–Whitney *U* test H_2_O Ti vs. H_2_O Tf: *U* = −34, *p* = 0.03121; H_2_O Tf vs. HTyr Tf: *U* = 17, *p* = 0.03019. Comamonadaceae: Kruskal–Wallis [df = 3; *H* = 12.894] *p* = 0.00487; Mann–Whitney *U* test H_2_O Ti vs. H_2_O Tf: *U* = −34, *p* = 0.03121; HTyr Ti vs. HTyr Tf: *U* = 18, *p* = 0.01965; H_2_O Tf vs. HTyr Tf: *U* = 24, *p* = 0.00503. *p < 0.05, **p < 0.01 Mann–Whitney *U* test. (E) LEfSe barplots showing differently enriched bacteria among groups (LDA score > 2.0, *p* < 0.05). Light blue bars: Genera enriched in H_2_O Ti vs. H_2_O Tf (up, left) and H_2_O Ti vs. HTyr Ti (down, left); blue bars: Genera enriched in H_2_O Tf vs. H_2_O Ti (up, left) and H_2_O Tf vs. HTyr Tf (down, right); light red bars: Genera enriched in HTyr Ti vs. HTyr Tf (up, right); red bars: Genera enriched in HTyr Tf vs. HTyr Ti (up, right) and HTyr Tf vs. H_2_O Tf (down, right). In the box plots, the line shows the median; the box, the interquartile range; the whiskers, the highest and lowest values; spare dots represent outliers.

In addition to the behavioral and immunohistochemical studies described above, we sought to investigate how the HTyr‐supplemented diet affected microbiota composition in the same 15‐month‐old mice previously examined, in the presence or absence of a traumatic experience, by using 16S rRNA gene sequencing. Specifically, we analyzed fecal samples from mice not subjected to behavioral testing (henceforth referred to as BT−) and mice that underwent contextual fear discrimination (CFD) and sensitization (FS) tests (henceforth referred to as BT+) treated with HTyr or H_2_O. Sample collection was carried out at the initial (Ti) and final (Tf) time points according to the experimental timeline represented in Figure 7A for BT− mice and Figure 8A for BT+ mice. For both BT− and BT+ mice, microbiota composition was compared between the following conditions: (i) H_2_O Ti vs. H_2_O Tf; (ii) HTyr Ti vs. HTyr Tf; (iii) H_2_O Ti vs. HTyr Ti; (iv) H_2_O Tf vs. HTyr Tf (Figures 7A and 8A). Comparison (iii) was included to assess baseline differences in microbiota composition between the groups of mice randomly assigned to the HTyr or H_2_O treatment.

#### 3.8.1 Dataset

Two samples were discarded for low‐quality sequencing outputs. A total of 76 samples were retained for subsequent analyses, corresponding to 7 613 089 row reads. After quality filtering, 5 593 509 valid reads were retained. Following ASVs clustering, chimera and singleton removal, 4 291 729 reads were clustered into 10 743 valid ASVs. After taxonomic assignment, ASVs with less than 70% identity to any reference sequence and those assigned as Chloroplasts were discarded. The final dataset obtained consisted of 10 713 ASVs, corresponding to 4 290 103 reads.

Raw reads are available in NCBI Sequence Read Archive (SRA), Bioproject PRJNA1290279.

#### 3.8.2 Community Diversity and Composition in Unstressed Mice

HTyr treatment in the absence of a traumatic experience (BT− mice) did not lead to significant changes in the diversity or overall composition of bacterial communities. Alpha diversity indexes (Shannon, Simpson, Richness, Evenness) showed no statistically significant differences (Figure S3A), and no distinct clustering of community composition (beta diversity) was found across comparisons (Figure S3B). Firmicutes and Bacteroidetes were the most abundant phyla across all sample groups, accounting on average for 85.5% ± 2.4% of total reads (Figure 7B). Their ratio (F/B), often considered a marker of dysbiosis when altered, showed no significant changes across comparisons (Figure S4). The relative abundance of the 12 most abundant phyla (Figure 7B) did not show significant difference between the two time points within each experimental group. When comparing H_2_O‐ and HTyr‐treated mice, statistically significant differences were observed only for the Acidobacteria phylum at Ti, indicating the presence of baseline differences between the two groups of mice for this phylum (Figure S5). At the family level, Muribaculaceae (phylum Bacteroidetes) and Lachnospiraceae (phylum Firmicutes) were the most abundant taxa across all groups, followed by other families with lower relative abundances (Figure 7C). No significant differences in the relative abundance of these families were observed across comparisons (Figure S6). LEfSe analysis was used to identify genera that were statistically different between groups. Comparing H_2_O Ti and H_2_O Tf samples, we observed a significant enrichment of *Blautia* and unidentified genera belonging to the class Clostridia and the order Desulfovibrionales in Ti compared to Tf samples. Conversely, *Lactobacillus* was significantly enriched in H_2_O Tf samples compared to H_2_O Ti samples (Figure 7D). An unclassified genus belonging to Desulfovibrionales order was also significantly enriched in HTyr Ti vs. HTyr Tf samples. Comparing H_2_O‐ and HTyr‐treated mice, we found a significant enrichment of a genus belonging to the *Erysipelotrichaceae* family at the beginning of the treatment (i.e., H_2_O Ti vs. HTyr Ti samples), whereas no differentially enriched genera were found at the end of the treatment (i.e., H_2_O Tf vs. HTyr Tf mice).

#### 3.8.3 Community Diversity and Composition in Stressed Mice

The potential of HTyr in modulating gut microbiota responses to a traumatic experience was evaluated by comparing bacterial community diversity and composition at the beginning of the experiment (Ti) and 12 days (Tf) after exposure to the contextual fear discrimination (CFD) and sensitization (FS) tests (see Figure 8A for timeline). In both H_2_O‐ and HTyr‐treated mice, alpha diversity indexes (Shannon, Simpson, Richness, Evenness) (Figure S7A) and overall bacterial community composition (Figure S7B) remained stable across all comparisons, with no statistically significant differences observed. As for microbial composition, we identified the 9 most abundant phyla, with Firmicutes and Bacteroidetes dominating across all sample groups, accounting on average for 91.2% ± 3.1% of total reads (Figure 8B). The F/B ratio remained stable both within each experimental group (i.e., HTyr or H_2_O Ti vs. Tf samples) and between H_2_O‐ and HTyr‐treated mice (Figure S8). Similarly, the relative abundance of the identified phyla did not differ significantly across all comparisons (Figure S9). At the family level, the dominant taxa across sample groups were Muribaculaceae (34.6% ± 8.4%) and Lachnospiraceae (22.7% ± 8.5%), followed by Rickenellaceae (10.6% ± 3.73%), Bacteroidaceae (5.6% ± 3.1%), Ruminococcaceae (4.9% ± 2.4%), and several others present at lower levels (Figure 8C). Between the most represented families, a statistically significant difference was detected for Ruminococcaceae, which decreased at Tf in H_2_O‐drinking mice. Among the less prevalent families, Desulfovibrionaceae, Pseudomonadaceae, Comamonadaceae, Aeromonadaceae, and Clostridiaceae exhibited significant changes across comparisons, albeit with distinct trends (Figure 8D and Figure S10).

The LEfSe analysis showed *Desulfovibrio* to be enriched in H_2_O Ti compared to H_2_O Tf samples, along with the genera *Clostridium* cluster XIVa, *Ruminococcus*, and *Enterorhabdus* (Figure 8E). *Clostridium* cluster XIVa and *Ruminococcus* were also enriched in HTyr Ti compared to HTyr Tf samples, along with *Eubacterium*. The comparison between H_2_O‐ and HTyr‐treated mice revealed an enrichment of *Pseudomonas* and *Comamonas* in H_2_O Tf samples, whereas *Aeromonas* and *Blautia* were enriched in HTyr Tf samples (Figure 8E).

## 4 Discussion

### 4.1 Effect of HTyr Treatment on Neurogenesis in the Dorsal and Ventral Dentate Gyrus

We have previously demonstrated that 30‐day treatment with HTyr increases the production of new neurons in the dentate gyrus of aged mice by stimulating the proliferation of both stem/progenitor (type‐1‐2a, Ki67^+^/Sox2^+^) and progenitor/neuroblast cells (type‐2b‐3, Ki67^+^/DCX^+^), as well as increasing neuron survival (D'Andrea et al. 2020). This dual effect on stem/progenitor cell proliferation and neuron survival is in line with HTyr's broader neuroprotective properties observed in animal models of age‐related neurodegenerative diseases, such as Parkinson's and Alzheimer's disease (see for review Micheli et al. 2023). Furthermore, we observed that the new neurons generated during HTyr treatment are recruited to existing neural circuits in the dentate gyrus, as evidenced by c‐fos activation (D'Andrea et al. 2020).

In this study, we asked whether the neurogenic action of HTyr has a functional effect on dentate gyrus‐dependent cognitive tasks. To this aim, we first examined whether 30 days of treatment with HTyr in aged mice selectively affects adult neurogenesis occurring in the dorsal or ventral regions of the dentate gyrus. Our findings revealed a significant increase in new neurons (BrdU^+^/NeuN^+^ cells) in both regions. Similarly, prolonged HTyr treatment for 50 days led to a rise in the total number of neuroblasts and early postmitotic neurons (type‐3 and type‐5, respectively; DCX^+^ cells) in both areas of the dentate gyrus. However, an analysis of proliferating stem and progenitor cells (i.e., Ki67^+^/Sox2^+^ and Ki67^+^/DCX^+^ cells, respectively) after 50 days of HTyr treatment revealed a significant increase in proliferation only in the ventral dentate gyrus, while the dorsal region exhibited a positive trend, which could explain the accumulation of neuroblasts and early postmitotic neurons (DCX^+^) observed in both regions. These findings suggest that the entire dentate gyrus is activated, although with region‐dependent temporal differences. Overall, our data show that HTyr has a pro‐neurogenic effect in both the dorsal and ventral dentate gyrus after either 30 or 50 days of treatment.

Interestingly, a morphological analysis of DCX^+^ immature neurons revealed a significant increase in dendritic complexity specifically in the ventral region of the dentate gyrus in HTyr‐treated mice, suggesting that HTyr can also enhance maturation and plasticity of newly generated neurons in this region, which may contribute to functional outcomes.

Since neurogenesis in the dorsal and ventral dentate gyrus has been related to associative memory performance and stress/anxiety regulation, respectively (Kjelstrup et al. 2002; Bannerman et al. 2004; Wu and Hen 2014), our results encouraged us to investigate the effect of HTyr treatment on both the memory and stress/anxiety response in aged mice.

### 4.2 Memory and Posttraumatic Anxiety‐Like Behaviors Associated With the Effects of HTyr on Neurogenesis in the Dorsal/Ventral Dentate Gyrus

Concerning memory, we tested the ability of aged mice treated with HTyr to discriminate between two very similar contexts, a cognitive process referred to as “pattern separation,” which is known to be related to adult neurogenesis occurring in the dorsal dentate gyrus (Kheirbek et al. 2013; França et al. 2017). It is worth noting that the age‐dependent decline in adult hippocampal neurogenesis impairs pattern separation in mice and that a pro‐neurogenic stimulus such as exercise can restore this ability (McAvoy and Sahay 2017; Wu et al. 2015). Contrary to our prediction, the pro‐neurogenic action of HTyr observed in the dorsal dentate gyrus of aged mice failed to counteract the age‐related deficit in pattern separation. This may be attributed to the fact that, although the neurogenic effect of HTyr is significant, it is quantitatively insufficient to improve discrimination between overlapping experiences, which is indeed a demanding cognitive task.

Adult ventral hippocampal neurogenesis is a key factor in determining the levels of vulnerability to stress and related psychiatric disorders (O'Leary and Cryan 2014; Anacker et al. 2018; Gomes‐Leal 2021; Liu et al. 2023). Chemogenic‐induced inhibition of hippocampal neurogenesis has been shown to increase anxiety‐like behaviors in the open field and elevated plus maze tests after fear conditioning due to a non‐associative fear sensitization (Seo et al. 2015). Furthermore, it has been shown that stressful experiences negatively affect neurogenesis preferentially in the ventral part of the dentate gyrus (Hawley et al. 2012; Elizalde et al. 2010) and that adult‐born neurons in this region—rather than the dorsal dentate gyrus—are required for the anxiolytic effect of the antidepressant fluoxetine in mice submitted to a traumatic experience in a contextual fear discrimination task (Wu and Hen 2014).

Our results show for the first time that HTyr treatment can protect against the time‐dependent development of non‐associative fear sensitization after a traumatic experience (fear incubation) and subsequent posttraumatic anxiety‐like behavior (Figure 4). It can therefore be inferred that this behavioral response is related to the neurogenic effect of HTyr, particularly in the ventral part of the dentate gyrus. Furthermore, we can assume that HTyr has a potent effect since newborn neurons, mainly in the ventral hippocampus, are known to be negatively affected by stressful experiences (Hawley et al. 2012; Elizalde et al. 2010). Remarkably, however, 50 days of HTyr treatment in mice undergoing behavioral testing resulted in a significant increase in the number of activated neurons in both the ventral and dorsal dentate gyrus (about 25% in each), as judged by the abundance of c‐fos^+^‐labeled cells (Figure 5). This finding suggests that not only the ventral but also the dorsal dentate gyrus is involved, to some extent, in the stress response in HTyr‐treated mice. This possibility is in line with the findings of Kheirbek et al. (2013), who demonstrated that, while a negative regulation of anxiety is exerted mainly by the activation of the ventral dentate gyrus, the activation of the dorsal dentate gyrus may also play a role in this process.

Notably, given that c‐fos activation is transient, lasting only few hours, it mainly reflects the most recent stimulus experienced by the mouse (i.e., Plus Maze Test 2). Nevertheless, newborn neurons are thought to be recruited to neural circuits 3‐to 6 weeks after their birth and thus to become functionally activable, that is, able to express c‐fos^+^ (Farioli‐Vecchioli et al. 2008; Kee et al. 2007). This period is considered a critical window where these new neurons become functionally integrated and contribute to learning and memory. Thus, the observed increase in activated c‐fos^+ ^neurons across both dentate gyrus regions could depend on the cumulative recruitment of new neurons during the behavioral tests conducted after day 20 of treatment, including contextual fear discrimination and fear sensitization procedures, which are expected to impact the neural circuit activity of both the dorsal and ventral dentate gyrus.

Overall, our data suggest that new neurons generated in the dorsal and ventral dentate gyrus, following treatment with HTyr, are both functionally involved in hippocampus‐dependent cognitive tasks, prevalently in anxiety/stress‐dependent tasks. Notably, the increased dendritic complexity of DCX^+^ immature neurons, observed in the ventral region of the dentate gyrus in HTyr‐treated mice, suggests a predominant contribution of this area.

Interestingly, we observed that treatment with HTyr can also modulate the activity of the microglial population in stressed mice by reducing the number of Iba1‐positive cells, as well as their soma area and ramification index. This may be correlated with a reduction in neuroinflammation, since the enlargement of soma and increased ramification are considered characteristic features of hyper‐activated microglia (McGill et al. 2018; He et al. 2024). It is known that many physiological and pathological conditions, including aging and anxiety, are characterized by a neuroinflammatory status, with the expansion of the microglial population (reviewed in Norden and Godbout 2013; Enomoto and Kato 2021). This expansion is among the factors responsible for the reduced proliferation of stem and progenitor cells in neurogenic niches (Cornejo and von Bernhardi 2016). In particular, the increase in inflammation with aging is due to the accumulation of free radical damage over time and is associated with microglial priming. This leads to age‐related cognitive decline and neural plasticity reduction (Chen et al. 2008). In addition, traumatic experiences can trigger neuroinflammation, and in patients with PTSD, there is an imbalance in the release of inflammatory mediators by microglia as well as in the levels of neurotrophic factors (Hori and Kim 2019; Green et al. 2013). In general, microglia are involved in the regulation of neurogenesis, immunological surveillance, redox imbalance, and cognitive and behavioral changes under normal and pathological conditions (Norden and Godbout 2013). Therefore, our results suggest that HTyr may regulate neurogenesis also by reducing the inflammatory status of microglia due to its anti‐inflammatory and antioxidant properties.

It is worth noting that an altered inflammatory response characterizes not only aging but also chronic immune‐mediated inflammatory diseases. In such pathologies, immune molecules can enter the brain, activating resident immune cells like microglia and astrocytes, triggering neuroinflammation. These conditions can impair hippocampal neurogenesis and disrupt several neurological functions, contributing to psychiatric symptoms in different pathologies. In particular, in the Experimental Autoimmune Encephalomyelitis (EAE) rodent model of multiple sclerosis (MS), it was observed that pro‐inflammatory cytokines released by activated microglia and immune cells alter synaptic plasticity, impair long‐term potentiation (LTP), and reduce neurogenesis (Huehnchen et al. 2011; Giannakopoulou et al. 2017). These alterations may underlie cognitive deficits and mood disturbances, including depressive symptoms observed in EAE and in MS patients even at the early stages of the disease (Bruno et al. 2021).

Interestingly, polyphenols of olive oil are currently being investigated as potential adjuvant therapies for MS (Conde et al. 2018) and other autoimmune diseases.

Regarding the possible molecular targets of HTyr, it is known that it exerts its beneficial effects by targeting multiple hallmarks of aging, including chronic inflammation, elevated oxidative stress, mitochondrial dysfunction, neural stem cell depletion, impaired protein homeostasis, and reduced autophagy. These pleiotropic actions of HTyr are largely attributed to its ability to activate key regulators of molecular pathways involved in autophagy, energy metabolism, mitochondrial biogenesis, and antioxidant defense (as reviewed in Micheli et al. 2023). Among these regulators are SIRT1, AMPK, FOXO, PGC1α, and Nrf2, which collectively orchestrate cellular resilience and longevity. Nevertheless, further studies are needed to fully elucidate the molecular mechanisms underlying the effects observed in our study.

### 4.3 HTyr Treatment Helps Preserve the Composition of Gut Beneficial Bacteria After Behavioral Induction of PTSD Symptoms

The microbiota‐gut‐brain axis is now recognized as a neuroendocrine system that acts bidirectionally and plays an important role in neurologic disorders and stress responses (Filosa et al. 2018). In particular, SCFAs produced by microbial fermentation of dietary indigestible fibers enhance microglia maturation and regulate neurotrophic factor levels, thereby enhancing neurogenesis and biosynthesis of serotonin, and improving neuronal function (Silva et al. 2020).

In this study, we analyzed the gut microbiota composition of aged mice fed a diet with or without HTyr supplementation, both in the presence and absence of a traumatic experience, to evaluate whether behavioral testing and/or HTyr administration could modulate the abundance of bacterial taxa known to be involved in gut‐brain signaling. In our analysis, we compared fecal samples between Ti and Tf time points within each treatment group (HTyr and H_2_O), as well as between the treatments at the same time point. In both unstressed and stressed mice, alpha and beta diversity indices remained largely stable across different treatments and time points, suggesting that neither HTyr administration nor the traumatic experience markedly affected gut microbial diversity and composition.

However, stress conditions significantly altered the relative abundance of four families, namely, Ruminococcaceae, Desulfovibrionaceae, Pseudomonadaceae, and Comamonadaceae (Figure 8D and Figure S10). Ruminococcaceae and Desulfovibrionaceae families significantly decreased in H_2_O‐drinking mice at Tf compared to Ti, while remaining stable over time upon HTyr administration (Figure 8D). Consistently, LEfSe analysis revealed that *Ruminococcus* and *Desulfovibrio* genera were enriched at Ti compared to Tf in stressed mice receiving H_2_O.

The Ruminococcaceae family possesses a wide array of carbohydrate‐active enzymes that enable the degradation of host‐indigestible substrates thus leading to the production of SCFAs, which provide energy to the host (Biddle et al. 2013) and modulate microbiota‐gut‐brain communication (Silva et al. 2020). Notably, Ruminococcaceae are reported to be depleted in individuals with major depressive and anxiety generalized disorders (Radjabzadeh et al. 2022; Knuesel and Mohajeri 2021; Liu et al. 2020), underscoring the relevance of SFCAs‐producing bacteria in mental health control. In healthy subjects, Ruminococcaceae abundance was found to be closely related to glutamate levels, which can signal to the brain via the vagus nerve (Altaib et al. 2021; Kaelberer et al. 2018) to support neuronal survival, synaptic plasticity, and adult hippocampal neurogenesis through activation of NMDA receptors on neural stem cells (Platel et al. 2010; Shohayeb et al. 2018). In addition, the Ruminococcaceae family was described as positively correlated with the levels of hippocampal BDNF (Ma et al. 2023), a key inducer of adult neurogenesis (Quesseveur et al. 2013) whose reduction is associated with depression and anxiety (Porter and O'Connor 2022), and negatively correlated with the levels of pro‐inflammatory cytokines such as IL‐1 (Ma et al. 2023).

The Desulfovibrionaceae family includes sulfate‐reducing bacteria, that are major gut producers of hydrogen sulfide (H_2_S), a gaseous signaling molecule that modulates inflammation, oxidative stress, vascular tone, and immune responses, consequently influencing brain functions (Andrés et al. 2023). The gut microbiota accounts for over 65% of circulating H_2_S and sulfane sulfur (Shen et al. 2013) whose effects are dose‐dependent: physiological levels are cytoprotective, whereas dysregulation is associated with disease (Cirino et al. 2023). In the mammalian brain, H_2_S enhances NMDA receptor activity and supports hippocampal plasticity and neurogenesis in mouse models of Parkinson disease (Kimura 2010; Wang et al. 2021), whereas reduced levels have been observed in neurodegenerative conditions (Nagpure and Bian 2015; Disbrow et al. 2021; Dwyer et al. 2004). Notably, impaired H_2_S production in mice is associated with anxiety‐prone phenotypes (Nagahara et al. 2013), while exogenous H_2_S shows antidepressant and anxiolytic effects in various animal models (Chen et al. 2013; Donatti et al. 2017), alleviates PTSD‐like behaviors, and improves hippocampal plasticity via the PI3K/AKT pathway in stressed rats (Yu et al. 2024). Based on our findings, the stress‐induced reduction of the Desulfovibrionaceae family in H_2_O‐drinking mice may lower endogenous H_2_S levels, potentially affecting gut‐brain axis signaling. LEfSe analysis also indicated that, in unstressed mice, a genus within the order Desulfovibrionales was enriched at Ti compared to Tf in both H_2_O‐ and HTyr‐drinking mice (Figure 7D). These findings suggest that, under physiological conditions, the decrease in sulfate‐reducing bacteria may be age‐dependent and not influenced by HTyr administration. On the other hand, under stress conditions, HTyr appears to prevent this decline, potentially contributing to the alleviation of PTSD‐like behavior, as discussed above.

The Pseudomonadaceae family relative abundance significantly increased over time in stressed H_2_O‐drinking mice, while remaining stable upon HTyr administration (Figure 8D and Figure S10). Consistently, LEfSe analysis revealed an enrichment of the *Pseudomonas* genus at Tf in stressed mice receiving H_2_O compared to those treated with HTyr (Figure 8D). As Pseudomonadaceae family includes commensal or pathogenic species and members of this family have been associated with bipolar disorder in humans (Zheng et al. 2020), our findings support the potential role of HTyr in maintaining a healthy gut‐microbiota homeostasis under stress.

Lastly, the Comamonadaceae family significantly increased over time in stressed H_2_O‐drinking mice, while decreasing upon HTyr administration (Figure 8D). Accordingly, LEfSe analysis showed enrichment of the *Comamonas* genus at Tf in stressed mice receiving H_2_O compared to HTyr‐treated ones (Figure 8D). Comamonadaceae are typically low abundance, context‐dependent gut microbes that have occasionally been associated with gastrointestinal and abdominal infections (Zhang et al. 2023), However, fluctuations in their relative abundance have more frequently been associated with dietary influences (Guo et al. 2021) or age‐related microbial shifts rather than with disease specific processes (Siddiqui et al. 2022).

Interestingly, we also found the genus *Blautia* enriched in unstressed H_2_O‐treated mice at Ti compared to Tf (Figure 7D), as well as in stressed mice receiving HTyr compared to H_2_O‐treated mice at Tf (Figure 8E). *Blautia* belongs to the Lachnospiraceae family, one of the most abundant families of Firmicutes in the gut, accounting for approximately 50% of phylotypes (Biddle et al. 2013). The Lachnospiraceae family possesses a strong saccharolytic capacity that leads to the production of butyrate and other SCFAs (Biddle et al. 2013). Many of the beneficial properties of this genus are attributed to its potential probiotic function, particularly the ability to convert plant bioactive substances, such as polyphenolics, into secondary metabolites with anticancer, anti‐inflammatory, antiviral, anticoagulant activities, and other biological functions (Burapan et al. 2017; Liu et al. 2021). *Blautia* abundance has been negatively associated with anxiety disorders (Li et al. 2024) and Parkinson's disease (Bonnechère et al. 2022), and in vivo administration of *Blautia stercoris* was shown to attenuate social deficits, repetitive behaviors, and anxiety‐like behavior in a mouse model of autism (Sen et al. 2022), highlighting its relevance to host neurobehavioral health. As in our study the abundance of *Blautia* was enriched at Ti compared to Tf in unstressed H_2_O‐drinking mice, while no significant change was observed in mice receiving HTyr, we can infer that HTyr may help maintain *Blautia* stability under physiological conditions. On the other hand, under stress condition, *Blautia* was enriched in HTyr‐treated compared to H_2_O‐drinking mice at Tf, suggesting that HTyr‐induced enrichment of *Blautia* may contribute to microbiota‐mediated stress resilience in mice.

Finally, LEfSe analysis showed that the probiotic *Clostridium* cluster XIVa genera were enriched in stressed mice at Ti compared to Tf, regardless of H_2_O‐ and HTyr supplementation. In contrast, the butyrate‐producing *Clostridium sensu strictu* cluster displayed a distinct pattern, being specifically enriched in HTyr‐treated mice at Tf compared to Ti, underscoring the potential of HTyr to selectively promote the growth of beneficial taxa involved in gut‐brain axis regulation.

Overall, our metabarcoding analyses suggest that HTyr may help stabilize health‐associated microbial communities, mitigating the negative impact of stress on gut microbial composition in aged mice.

## 5 Conclusions

Here, we observe that treatment with HTyr in aging mice enhances the production of new neurons and neuroblasts in both the dorsal and ventral dentate gyrus. However, a significant increase in the number of proliferating stem cells and neuroblasts is observed only in the ventral dentate gyrus. We conclude that HTyr treatment increases neurogenesis in both regions, with a predominant effect in the ventral region. It is known that associative memory and stress response are integrated by the dorsal and ventral dentate gyrus, respectively. However, the pro‐neurogenic effect of HTyr in aged mice is not associated with an improved contextual memory discrimination. Instead, a significant reduction in PTSD‐like anxiety symptoms is observed, which we suggest may be linked to increased neurogenesis in the ventral region. Furthermore, in the dentate gyrus of mice exposed to stress conditions, HTyr demonstrates the ability to reduce neuroinflammation. In parallel, although HTyr treatment does not alter overall microbiota diversity, it appears to help preserve the stability of some bacterial families and genera under stress conditions, such as the Ruminococcaceae, Desulfovibrionaceae, and Pseudomonadaceae families and the *Blautia* genus, potentially mitigating stress‐related disruptions in gut‐brain signaling.

**FIGURE 9:**
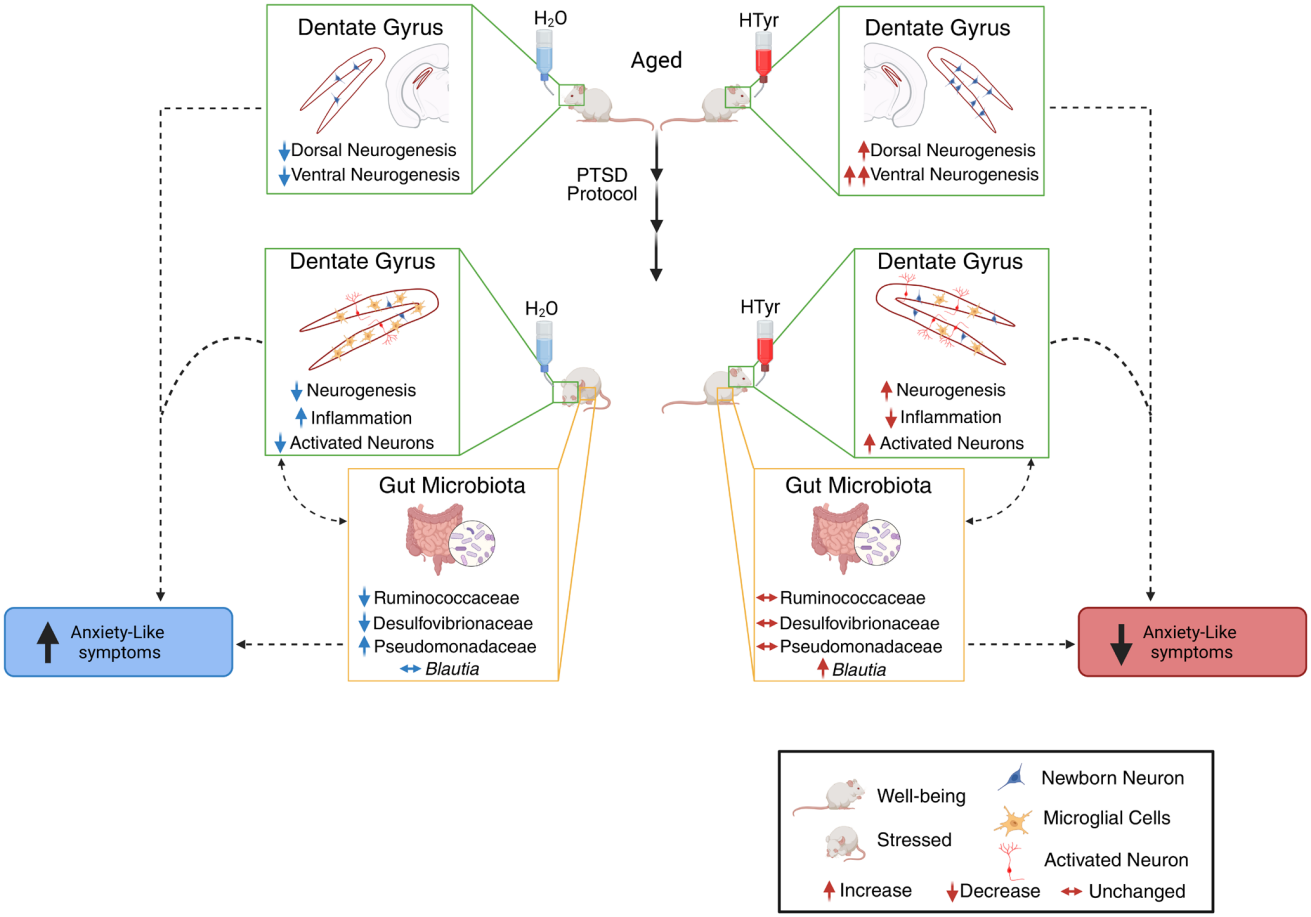
Schematic overview of HTyr effects on anxiety‐like behavior and its potential correlation with hippocampal neurogenesis, neuroinflammation, and gut microbiota composition in aged mice. PTSD: Post traumatic stress disorder model. Figure created with BioRender (https://BioRender.com/l1vskoi).

Overall, the data presented here demonstrate that HTyr treatment alleviates anxiety‐like behavior following a traumatic event. This effect may be mediated by enhanced neurogenesis in the ventral dentate gyrus, with HTyr acting directly on the brain and/or indirectly through the microbiota‐gut‐brain axis (see a schematic representation of results and conclusions in Figure 9). These findings suggest the potential of HTyr as a candidate for the development of therapeutic strategies targeting PTSD. Additional investigations will be required to delineate the mechanisms and pathways involved.

## Author Contributions

**Marco Costanzi:** conceptualization, investigation, validation, writing – review and editing, formal analysis, writing – original draft. **Giorgio D'Andrea:** conceptualization, investigation, validation, writing – review and editing, formal analysis. **Laura Bertini:** conceptualization, investigation, writing – original draft, writing – review and editing, formal analysis, data curation, validation. **Andrea Fochetti:** investigation, methodology. **Roberta Bernini:** methodology, investigation, writing – review and editing. **Mariangela Clemente:** investigation, methodology. **Silvia Proietti:** investigation, validation, data curation. **Manuela Ceccarelli:** investigation. **Maurizia Caruso:** writing – review and editing, formal analysis, conceptualization. **Carla Caruso:** writing – review and editing, formal analysis, supervision. **Ferdinando Scavizzi:** resources, investigation. **Marcello Raspa:** resources. **Felice Tirone:** supervision, formal analysis, project administration, writing – review and editing, writing – original draft, funding acquisition, conceptualization. **Laura Micheli:** supervision, conceptualization, writing – review and editing, writing – original draft, funding acquisition, project administration, formal analysis, visualization, validation.

## Funding

This work was supported by the POR FESR Lazio 2014–2020 “Progetti Gruppi di Ricerca 2020” Regione Lazio n. A0375‐2020‐36407 (CNR project DSB.AD004.367), by the Fondazione Adriano Buzzati‐Traverso grant 2020–2024 (CNR project DSB.AD004.317) and by the project of Associazione per le Neuroscienze Giuseppe Moruzzi (CNR project DSB.AD004.514) to F.T. This work was also supported by Consiglio Nazionale delle Ricerche FOE 2021 NUTRAGE project (CNR project DBA.AD005.225) to L.M.

## Ethics Statement

All animal procedures were performed in accordance with the current guidelines of the European Ethical Committee (directive 2010/63/EU) and with the experimental protocol approved by the Italian Ministry of Health (Authorization N. 364‐2020‐PR).

## Conflicts of Interest

The authors declare no conflicts of interest.

## Supporting information


**Figure S1:** Morphological analysis of DCX‐positive cells of mice treated as described in Figure 2A. Skeleton and Sholl analyses revealed a significant increase in the number (#) of branches and junctions in mice treated with HTyr, particularly in the ventral region of the dentate gyrus. Continuous outcomes (dendritic length) were analyzed using a LMM statistical analysis with a random intercept for Mouse; fixed‐effect significance was assessed using Wald *F*‐tests. Count outcomes were analyzed using GLMM‐NB statistical analysis and a random intercept for Mouse; significance was assessed using Wald *χ* ^2^ test. Planned simple‐effects analyses were performed to evaluate the effect of treatment within each region. Data are means ± SEM. *n* = 82 DCX^+^ cells. At least, 9 DCX^+^ cells/mouse were analyzed. Each group consisted of 4 mice. *p < 0.05, **p < 0.01, NS *p* > 0.05. LMM, linear mixed models; GLMM‐NB, generalized linear mixed models with a negative binomial distribution.
**Figure S2:** (A) Schematic representation of the experimental plan. Percentage of freezing showed by HTyr (*n* = 9, red) and H_2_O (*n* = 9, blue) treated mice in (B) the contextual fear conditioning tests (1 and 16 days after training) and in (C) the context generalization tests in the two decidedly different contexts C and D (see text for details in the procedure). All the data are shown as mean ± SEM. Statistical analyses were carried out by two‐way ANOVA for contextual memory tests, and Two‐tailed Student's *t*‐test for context generalization tests. NS, *p* > 0.05. OF, Open Field; PM, plus maze; squares A and B: context A and B; circles A and B: context C and D.
**Figure S3:** (A) Boxplots of Richness, Simpson, Shannon, and Evenness indexes in BT− mice for H_2_O and HTyr treatment at the initial (Ti) and final (Tf) time points. No statistically significant differences between experimental groups were detected based on the Kruskal–Wallis test. Richness: *H* = 6.5146, df = 3, *p* = 0.08909; Simpson index: *H* = 7.7068, df = 3, *p* = 0.05248; Shannon index: *H* = 6.505, df = 3, *p* = 0.08947; Evenness: *H* = 0.7315, df = 3, *p* = 0.8658. (B) NMDS ordinations of communities' composition in BT− mice using the Bray‐Curtis distance metric of Hellinger transformed OUT abundances. Clustering significance was assessed by PERMANOVA. For all comparisons *p* > 0.05. In the box plots, the line shows the median; the box, the interquartile range; the whiskers, the highest and lowest values; spare dots represent outlier.
**Figure S4:** Boxplots of Firmicutes/Bacteroidetes ratio in BT− mice for H_2_O and HTyr treatment at the initial (Ti) and final (Tf) time points. No statistically significant differences between samples were detected based on the Kruskal–Wallis test (*H* = 1.1163, df = 3, *p* = 0.7731). In the box plots, the line shows the median; the box, the interquartile range; the whiskers, the highest and lowest values; spare dots represent outliers.
**Figure S5:** Boxplots of relative abundance of bacterial phyla in BT− mice for H_2_O and HTyr treatment at the initial (Ti) and final (Tf) time points. Kruskal–Wallis test was performed to assess significant differences across treatments. The comparisons between groups were based on two‐tailed Mann–Whitney *U* test. Acidobacteriaceae: *H* = 11.058, df = 3, *p* = 0.01142; *U* = 23, *p* = 0.02597. No significant phyla: df = 3, *H* ≤ 6.062, *p* > 0.05. Statistically significant differences are marked with asterisks (*p < 0.05, Mann–Whitney *U* test). In the box plots, the line shows the median; the box, the interquartile range; the whiskers, the highest and lowest values; spare dots represent outliers.
**Figure S6:** Boxplots of relative abundance of bacterial families in BT− mice for H_2_O and HTyr treatment at the initial (Ti) and final (Tf) time points. No statistically significant differences between groups were detected based on Kruskal–Wallis test (df = 3, *H* ≤ 5.534, *p* > 0.05). In the box plots, the line shows the median; the box, the interquartile range; the whiskers, the highest and lowest values; spare dots represent outliers.
**Figure S7:** (A) Boxplots of Richness, Simpson, Shannon and Evenness indexes in BT+ mice for H_2_O and HTyr treatment at the initial (Ti) and final (Tf) time points. No statistically significant differences between samples were detected based on Kruskal–Wallis test (Richness: *H* = 5.8505, df = 3, *p* = 0.119; Simpson index: *H* = 6.075, df = 3, *p* = 0.108; Shannon index: *H* = 4.650, df = 3, *p* = 0.199; Evenness: *H* = 2.739, df = 3, *p* = 0.433). (B) NMDS ordinations of communities' composition in BT+ mice using the Bray‐Curtis distance metric of Hellinger transformed OUT abundances. Clustering significance was assessed by PERMANOVA. For all comparisons *p* > 0.05. In the box plots, the line shows the median; the box, the interquartile range; the whiskers, the highest and lowest values; spare dots represent outliers.
**Figure S8:** Boxplots of Firmicutes/Bacteroidetes ratio in BT+ mice for H_2_O and HTyr treatment at the initial (Ti) and final (Tf) time points. No statistically significant differences between samples were detected based on Kruskal–Wallis test (*H* = 5.8416, df = 3, *p* = 0.1196). In the box plots, the line shows the median; the box, the interquartile range; the whiskers, the highest and lowest values; spare dots represent outliers.
**Figure S9:** Boxplots of relative abundance of bacterial phyla in BT+ mice for H_2_O and HTyr treatment at the initial (Ti) and final (Tf) time points. No statistically significant differences between samples were detected based on Kruskal–Wallis test (df = 3, *H* ≤ 6.466, *p* > 0.05). In the box plots, the line shows the median; the box, the interquartile range; the whiskers, the highest and lowest values; spare dots represent outliers.
**Figure S10:** Boxplots of relative abundance of bacterial Families in BT+ mice for H_2_O and HTyr treatment at the initial (Ti) and final (Tf) time points. Kruskal–Wallis test was used to evaluate overall differences across treatments. For significant families, pairwise comparisons were performed using two‐tailed Mann–Whitney *U* test. Ruminococcaceae: Kruskal–Wallis [df = 3; *H* = 10.693] *p* = 0.01351; Mann–Whitney *U* test H_2_O Ti vs. H_2_O Tf: *U* = 27, *p* = 0.01726. Desulfovibrionaceae: Kruskal–Wallis [df = 3; *H* = 8.1937] *p* = 0.04217; Mann–Whitney *U* test H_2_O Ti vs. H_2_O Tf: *U* = 29, *p* = 0.01133. Pseudomonadaceae: Kruskal–Wallis [df = 3; *H* = 8.1517] *p* = 0.04298; Mann–Whitney *U* test H_2_O Ti vs. H_2_O Tf: *U* = −34, *p* = 0.03121; H_2_O Tf vs. HTyr Tf: *U* = 17, *p* = 0.03019. Comamonadaceae: Kruskal–Wallis [df = 3; *H* = 12.894] *p* = 0.00487; Mann–Whitney *U* test H_2_O Ti vs. H_2_O Tf: *U* = −34, *p* = 0.03121; HTyr Ti vs. HTyr Tf: *U* = 18, *p* = 0.01965; H_2_O Tf vs. HTyr Tf: *U* = 24, *p* = 0.00503. Aeromonadaceae: Kruskal–Wallis [df = 3; *H* = 13.234] *p* = 0.0042; Mann–Whitney *U* test HTyr Ti vs. H_2_O Tf: *U* = −40, *p* = 0.00350. For families showing no significant differences: df = 3, *H* ≤ 6.044, *p* > 0.05. Statistically significant differences are marked with asterisks (*p < 0.05; **p < 0.01, Mann–Whitney *U* test). In the box plots, the line shows the median; the box, the interquartile range; the whiskers, the highest and lowest values; spare dots represent outliers.

## Data Availability

The data of immunofluorescence and of behavioral tests generated and analyzed during this study are included in this article and its Supporting Information files. The raw data are available from the corresponding author on reasonable request. Moreover, all relevant NMR spectral data can be provided upon request. The data that support the findings of the microbiota analysis are openly available in NCBI Sequence Read Archive (SRA), at https://www.ncbi.nlm.nih.gov/sra/PRJNA1290279, Bioproject PRJNA1290279.

## References

[jnc70448-bib-0001] Abdel Haq, R. , J. C. M. Schlachetzki , C. K. Glass , and S. K. Mazmanian . 2019. “Microbiome‐Microglia Connections via the Gut‐Brain Axis.” Journal of Experimental Medicine 216, no. 1: 41–59. 30385457 10.1084/jem.20180794 PMC6314531

[jnc70448-bib-0002] Adusumilli, V. S. , T. L. Walker , R. W. Overall , et al. 2021. “ROS Dynamics Delineate Functional States of Hippocampal Neural Stem Cells and Link to Their Activity‐Dependent Exit From Quiescence.” Cell Stem Cell 28, no. 2: 300–314.e6. 33275875 10.1016/j.stem.2020.10.019 PMC7875116

[jnc70448-bib-0003] Aimone, J. B. , W. Deng , and F. H. Gage . 2011. “Resolving New Memories: A Critical Look at the Dentate Gyrus, Adult Neurogenesis, and Pattern Separation.” Neuron 70, no. 4: 589–596. 21609818 10.1016/j.neuron.2011.05.010 PMC3240575

[jnc70448-bib-0004] Altaib, H. , K. Nakamura , M. Abe , et al. 2021. “Differences in the Concentration of the Fecal Neurotransmitters GABA and Glutamate Are Associated With Microbial Composition Among Healthy Human Subjects.” Microorganisms 9: 1–15. 10.3390/microorganisms9020378 PMC7918917 33668550

[jnc70448-bib-0005] Anacker, C. , and R. Hen . 2017. “Adult Hippocampal Neurogenesis and Cognitive Flexibility—Linking Memory and Mood.” Nature Reviews Neuroscience 18, no. 6: 335–346. 28469276 10.1038/nrn.2017.45 PMC6261347

[jnc70448-bib-0006] Anacker, C. , V. M. Luna , G. S. Stevens , et al. 2018. “Hippocampal Neurogenesis Confers Stress Resilience by Inhibiting the Ventral Dentate Gyrus.” Nature 559, no. 7712: 98–102. 29950730 10.1038/s41586-018-0262-4 PMC6118212

[jnc70448-bib-0007] Andrés, C. M. C. , J. M. Pérez de la Lastra , C. Andrés Juan , F. J. Plou , and E. Pérez‐Lebeña . 2023. “Chemistry of Hydrogen Sulfide—Pathological and Physiological Functions in Mammalian Cells.” Cells 12, no. 1: 1–27. 10.3390/cells12232684 PMC10705518 38067112

[jnc70448-bib-0008] Arshadi, C. , U. Günther , M. Eddison , K. I. S. Harrington , and T. A. Ferreira . 2021. “SNT: A Unifying Toolbox for Quantification of Neuronal Anatomy.” Nature Methods 18, no. 4: 374–377. 33795878 10.1038/s41592-021-01105-7

[jnc70448-bib-0009] Babcock, K. R. , J. S. Page , J. R. Fallon , and A. E. Webb . 2021. “Adult Hippocampal Neurogenesis in Aging and Alzheimer's Disease.” Stem Cell Reports 16, no. 3: 681–693. 33636114 10.1016/j.stemcr.2021.01.019 PMC8072031

[jnc70448-bib-0010] Bannerman, D. M. , J. N. P. Rawlins , S. B. McHugh , et al. 2004. “Regional Dissociations Within the Hippocampus—Memory and Anxiety.” Neuroscience & Biobehavioral Reviews 28, no. 3: 273–283. 15225971 10.1016/j.neubiorev.2004.03.004

[jnc70448-bib-0011] Barber, T. M. , S. Kabisch , A. F. H. Pfeiffer , and M. O. Weickert . 2023. “The Effects of the Mediterranean Diet on Health and Gut Microbiota.” Nutrients 15, no. 2150: 1–19. 10.3390/nu15092150 PMC10180651 37432307

[jnc70448-bib-0012] Bernini, R. , M. S. Gilardini Montani , N. Merendino , A. Romani , and F. Velotti . 2015. “Hydroxytyrosol‐Derived Compounds: A Basis for the Creation of New Pharmacological Agents for Cancer Prevention and Therapy.” Journal of Medicinal Chemistry 58, no. 22: 9089–9107. 26225717 10.1021/acs.jmedchem.5b00669

[jnc70448-bib-0013] Bernini, R. , N. Merendino , A. Romani , and F. Velotti . 2013. “Naturally Occurring Hydroxytyrosol: Synthesis and Anticancer Potential.” Current Medicinal Chemistry 20, no. 5: 655–670. 23244583 10.2174/092986713804999367

[jnc70448-bib-0014] Bernini, R. , E. Mincione , M. Barontini , and F. Crisante . 2008. “Convenient Synthesis of Hydroxytyrosol and Its Lipophilic Derivatives From Tyrosol or Homovanillyl Alcohol.” Journal of Agricultural and Food Chemistry 56, no. 20: 8897–8904. 18771272 10.1021/jf801558z

[jnc70448-bib-0015] Bettio, L. E. B. , L. Rajendran , and J. Gil‐Mohapel . 2017. “The Effects of Aging in the Hippocampus and Cognitive Decline.” Neuroscience & Biobehavioral Reviews 79: 66–86. 28476525 10.1016/j.neubiorev.2017.04.030

[jnc70448-bib-0016] Biddle, A. , L. Stewart , J. Blanchard , and S. Leschine . 2013. “Untangling the Genetic Basis of Fibrolytic Specialization by Lachnospiraceae and Ruminococcaceae in Diverse Gut Communities.” Diversity 5, no. 3: 627–640.

[jnc70448-bib-0017] Bizon, J. L. , and M. Gallagher . 2003. “Production of New Cells in the Rat Dentate Gyrus Over the Lifespan: Relation to Cognitive Decline.” European Journal of Neuroscience 18, no. 2: 215–219. 12859354 10.1046/j.1460-9568.2003.02733.x

[jnc70448-bib-0018] Boldrini, M. , C. A. Fulmore , A. N. Tartt , et al. 2018. “Human Hippocampal Neurogenesis Persists Throughout Aging.” Cell Stem Cell 22, no. 4: 589–599.e5. 29625071 10.1016/j.stem.2018.03.015 PMC5957089

[jnc70448-bib-0019] Bonnechère, B. , N. Amin , and C. van Duijn . 2022. “What Are the Key Gut Microbiota Involved in Neurological Diseases? A Systematic Review.” International Journal of Molecular Sciences 23, no. 22: 13665. 36430144 10.3390/ijms232213665 PMC9696257

[jnc70448-bib-0020] Brandt, M. D. , S. Jessberger , B. Steiner , et al. 2003. “Transient Calretinin Expression Defines Early Postmitotic Step of Neuronal Differentiation in Adult Hippocampal Neurogenesis of Mice.” Molecular and Cellular Neuroscience 24, no. 3: 603–613. 14664811 10.1016/s1044-7431(03)00207-0

[jnc70448-bib-0021] Bruno, A. , E. Dolcetti , F. R. Rizzo , et al. 2021. “Corrigendum: Inflammation‐Associated Synaptic Alterations as Shared Threads in Depression and Multiple Sclerosis.” Frontiers in Cellular Neuroscience 14: 647259. 33574741 10.3389/fncel.2020.647259 PMC7871045

[jnc70448-bib-0022] Burapan, S. , M. Kim , and J. Han . 2017. “Demethylation of Polymethoxyflavones by Human Gut Bacterium, *Blautia* sp. *MRG‐PMF1* .” Journal of Agricultural and Food Chemistry 65, no. 8: 1620–1629. 28211698 10.1021/acs.jafc.7b00408

[jnc70448-bib-0023] Callahan, B. J. , P. J. McMurdie , M. J. Rosen , A. W. Han , A. J. A. Johnson , and S. P. Holmes . 2016. “DADA2: High‐Resolution Sample Inference From Illumina Amplicon Data.” Nature Methods 13, no. 7: 581–583. 27214047 10.1038/nmeth.3869 PMC4927377

[jnc70448-bib-0024] Cameron, H. A. , C. S. Woolley , B. S. McEwen , and E. Gould . 1993. “Differentiation of Newly Born Neurons and Glia in the Dentate Gyrus of the Adult Rat.” Neuroscience 56, no. 2: 337–344. 8247264 10.1016/0306-4522(93)90335-d

[jnc70448-bib-0025] Ceccarelli, M. , G. D'Andrea , L. Micheli , and F. Tirone . 2020. “Interaction Between Neurogenic Stimuli and the Gene Network Controlling the Activation of Stem Cells of the Adult Neurogenic Niches, in Physiological and Pathological Conditions.” Frontiers in Neuroscience 8: 211. 10.3389/fcell.2020.00211 PMC7154047 32318568

[jnc70448-bib-0026] Chen, J. , J. B. Buchanan , N. L. Sparkman , J. P. Godbout , G. G. Freund , and R. W. Johnson . 2008. “Neuroinflammation and Disruption in Working Memory in Aged Mice After Acute Stimulation of the Peripheral Innate Immune System.” Brain, Behavior, and Immunity 22, no. 3: 301–311. 17951027 10.1016/j.bbi.2007.08.014 PMC2374919

[jnc70448-bib-0027] Chen, W. L. , B. Xie , C. Zhang , et al. 2013. “Antidepressant‐Like and Anxiolytic‐Like Effects of Hydrogen Sulfide in Behavioral Models of Depression and Anxiety.” Behavioural Pharmacology 24, no. 7: 590–597. 23974423 10.1097/FBP.0b013e3283654258

[jnc70448-bib-0028] Cirino, G. , C. Szabo , and A. Papapetropoulos . 2023. “Physiological Roles of Hydrogen Sulfide in Mammalian Cells, Tissues, and Organs.” Physiological Reviews 103, no. 1: 31–276. 35435014 10.1152/physrev.00028.2021

[jnc70448-bib-0029] Cole, J. R. , Q. Wang , J. A. Fish , et al. 2014. “Ribosomal Database Project: Data and Tools for High Throughput rRNA Analysis.” Nucleic Acids Research 42, no. D1: D633–D642. 24288368 10.1093/nar/gkt1244 PMC3965039

[jnc70448-bib-0030] Conde, C. , B. M. Escribano , E. Luque , et al. 2018. “The Protective Effect of Extra‐Virgin Olive Oil in the Experimental Model of Multiple Sclerosis in the Rat.” Nutritional Neuroscience 23, no. 1: 37–45. 29730972 10.1080/1028415X.2018.1469281

[jnc70448-bib-0031] Cornejo, F. , and R. von Bernhardi . 2016. “Age‐Dependent Changes in the Activation and Regulation of Microglia.” Advances in Experimental Medicine and Biology 949: 205–226. 27714691 10.1007/978-3-319-40764-7_10

[jnc70448-bib-0032] Costanzi, M. , S. Cannas , D. Saraulli , C. Rossi‐Arnaud , and V. Cestari . 2011. “Extinction After Retrieval: Effects on the Associative and Nonassociative Components of Remote Contextual Fear Memory.” Learning & Memory 18, no. 9: 508–518. 21764847 10.1101/lm.2175811

[jnc70448-bib-0033] Costanzi, M. , D. Saraulli , S. Cannas , et al. 2014. “Fear but Not Fright: Re‐Evaluating Traumatic Experience Attenuates Anxiety‐Like Behaviors After Fear Conditioning.” Frontiers in Behavioral Neuroscience 8: 279. 25202244 10.3389/fnbeh.2014.00279 PMC4142342

[jnc70448-bib-0034] Couillard‐Després, S. , C. Wuertinger , M. Kandasamy , et al. 2009. “Ageing Abolishes the Effects of Fluoxetine on Neurogenesis.” Molecular Psychiatry 14, no. 9: 856–864. 19139747 10.1038/mp.2008.147

[jnc70448-bib-0035] Cryan, J. F. , and T. G. Dinan . 2012. “Mind‐Altering Microorganisms: The Impact of the Gut Microbiota on Brain and Behaviour.” Nature Reviews Neuroscience 13, no. 10: 701–712. 22968153 10.1038/nrn3346

[jnc70448-bib-0036] Czéh, B. , and P. J. Lucassen . 2007. “What Causes the Hippocampal Volume Decrease in Depression? Are Neurogenesis, Glial Changes and Apoptosis Implicated?” European Archives of Psychiatry and Clinical Neuroscience 257, no. 5: 250–260. 17401728 10.1007/s00406-007-0728-0

[jnc70448-bib-0037] D'Andrea, G. , M. Ceccarelli , R. Bernini , et al. 2020. “Hydroxytyrosol Stimulates Neurogenesis in Aged Dentate Gyrus by Enhancing Stem and Progenitor Cell Proliferation and Neuron Survival.” FASEB Journal 34, no. 1: 4512–4526. 32027412 10.1096/fj.201902643R

[jnc70448-bib-0038] De La Cruz, J. P. , M. I. Ruiz‐Moreno , A. Guerrero , et al. 2015. “Differences in the Neuroprotective Effect of Orally Administered Virgin Olive Oil ( *Olea europaea* ) Polyphenols Tyrosol and Hydroxytyrosol in Rats.” Journal of Agricultural and Food Chemistry 63, no. 26: 5957–5963. 26066316 10.1021/acs.jafc.5b00627

[jnc70448-bib-0039] de Pablos, R. M. , A. M. Espinosa‐Oliva , R. Hornedo‐Ortega , M. Cano , and S. Arguelles . 2019. “Hydroxytyrosol Protects From Aging Process via AMPK and Autophagy: A Review of Its Effects on Cancer, Metabolic Syndrome, Osteoporosis, Immune‐Mediated and Neurodegenerative Diseases.” Pharmacological Research 143: 58–72. 30853597 10.1016/j.phrs.2019.03.005

[jnc70448-bib-0040] Disbrow, E. , K. Y. Stokes , C. Ledbetter , et al. 2021. “Plasma Hydrogen Sulfide: A Biomarker of Alzheimer's Disease and Related Dementias.” Alzheimer's & Dementia 17, no. 8: 1391–1402. 10.1002/alz.12305 PMC8451930 33710769

[jnc70448-bib-0041] Donatti, A. F. , R. N. Soriano , C. R. A. Leite‐Panissi , L. G. S. Branco , and A. S. de Souza . 2017. “Anxiolytic‐Like Effect of Hydrogen Sulfide (H_2_S) in Rats Exposed and Re‐Exposed to the Elevated Plus‐Maze and Open Field Tests.” Neuroscience Letters 642: 77–85. 28131657 10.1016/j.neulet.2017.01.059

[jnc70448-bib-0042] Dumitru, I. , M. Paterlini , M. Zamboni , et al. 2025. “Identification of Proliferating Neural Progenitors in the Adult Human Hippocampus.” Science 389, no. 6650: 58–63. 40608919 10.1126/science.adu9575

[jnc70448-bib-0043] Dwyer, B. E. , A. K. Raina , G. Perry , and M. A. Smith . 2004. “Homocysteine and Alzheimer's Disease: A Modifiable Risk?” Free Radical Biology and Medicine 36, no. 12: 1471–1475. 15135184 10.1016/j.freeradbiomed.2004.03.009

[jnc70448-bib-0044] Edgar, R. C. 2010. “Search and Clustering Orders of Magnitude Faster Than BLAST.” Bioinformatics 26, no. 19: 2460–2461. 20709691 10.1093/bioinformatics/btq461

[jnc70448-bib-0045] Elizalde, N. , A. L. García‐García , S. Totterdell , et al. 2010. “Sustained Stress‐Induced Changes in Mice as a Model for Chronic Depression.” Psychopharmacology 210, no. 3: 393–406. 20401750 10.1007/s00213-010-1835-6

[jnc70448-bib-0046] Enomoto, S. , and T. A. Kato . 2021. “Involvement of Microglia in Disturbed Fear Memory Regulation: Possible Microglial Contribution to the Pathophysiology of Posttraumatic Stress Disorder.” Neurochemistry International 142: 104926. 33232758 10.1016/j.neuint.2020.104921

[jnc70448-bib-0047] Farioli‐Vecchioli, S. , A. Mattera , L. Micheli , et al. 2014. “Running Rescues Defective Adult Neurogenesis by Shortening the Length of the Cell Cycle of Neural Stem and Progenitor Cells.” Stem Cells 32, no. 7: 1968–1982. 24604711 10.1002/stem.1679

[jnc70448-bib-0048] Farioli‐Vecchioli, S. , L. Micheli , D. Saraulli , et al. 2012. “Btg1 Is Required to Maintain the Pool of Stem and Progenitor Cells of the Dentate Gyrus and Subventricular Zone.” Frontiers in Neuroscience 6: 124. 22969701 10.3389/fnins.2012.00124 PMC3431174

[jnc70448-bib-0049] Farioli‐Vecchioli, S. , D. Saraulli , M. Costanzi , et al. 2008. “The Timing of Differentiation of Adult Hippocampal Neurons Is Crucial for Spatial Memory.” PLoS Biology 6, no. 10: e246. 18842068 10.1371/journal.pbio.0060246 PMC2561078

[jnc70448-bib-0050] Ferreira, T. A. , A. V. Blackman , J. Oyrer , et al. 2014. “Neuronal Morphometry Directly From Bitmap Images.” Nature Methods 11, no. 10: 982–984. 25264773 10.1038/nmeth.3125 PMC5271921

[jnc70448-bib-0051] Filippov, V. , G. Kronenberg , T. Pivneva , et al. 2003. “Subpopulation of Nestin‐Expressing Progenitor Cells in the Adult Murine Hippocampus Shows Electrophysiological and Morphological Characteristics of Astrocytes.” Molecular and Cellular Neuroscience 23, no. 5: 373–382. 12837622 10.1016/s1044-7431(03)00060-5

[jnc70448-bib-0052] Filosa, S. , F. Di Meo , and S. Crispi . 2018. “Polyphenols–Gut Microbiota Interplay and Brain Neuromodulation.” Neural Regeneration Research 13, no. 12: 2055–2059. 30323120 10.4103/1673-5374.241429 PMC6199944

[jnc70448-bib-0053] Fölsz, O. , S. Trouche , and V. Croset . 2023. “Adult‐Born Neurons Add Flexibility to Hippocampal Memories.” Frontiers in Neuroscience 17: 1128623. 36875670 10.3389/fnins.2023.1128623 PMC9975346

[jnc70448-bib-0054] França, T. F. A. , A. M. Bitencourt , N. R. Maximilla , D. M. Barros , and J. M. Monserrat . 2017. “Hippocampal Neurogenesis and Pattern Separation: A Meta‐Analysis of Behavioral Data.” Hippocampus 27, no. 9: 937–950. 28597491 10.1002/hipo.22746

[jnc70448-bib-0055] Fukuda, S. , F. Kato , Y. Tozuka , M. Yamaguchi , Y. Miyamoto , and T. Hisatsune . 2003. “Two Distinct Subpopulations of Nestin‐Positive Cells in Adult Mouse Dentate Gyrus.” Journal of Neuroscience 23, no. 27: 9357–9366. 14561863 10.1523/JNEUROSCI.23-28-09357.2003 PMC6740569

[jnc70448-bib-0056] Funakohi‐Tago, M. , T. Sakata , S. Fujiwara , et al. 2018. “Hydroxytyrosol Butyrate Inhibits 6‐OHDA‐Induced Apoptosis Through Activation of the Nrf2/HO‐1 Axis in SH‐SY5Y Cells.” European Journal of Pharmacology 834: 246–256. 30053409 10.1016/j.ejphar.2018.07.043

[jnc70448-bib-0057] Giannakopoulou, A. , G. A. Lyras , and N. Grigoriadis . 2017. “Long‐Term Effects of Autoimmune CNS Inflammation on Adult Hippocampal Neurogenesis.” Journal of Neuroscience Research 95, no. 7: 1446–1458. 27781303 10.1002/jnr.23982

[jnc70448-bib-0058] Gomes‐Leal, W. 2021. “Adult Hippocampal Neurogenesis and Affective Disorders: New Neurons for Psychic Well‐Being.” Frontiers in Neuroscience 15: 594448. 34220412 10.3389/fnins.2021.594448 PMC8242208

[jnc70448-bib-0059] Gould, E. , A. Beylin , P. Tanapat , A. Reeves , and T. J. Shors . 1999. “Learning Enhances Adult Neurogenesis in the Hippocampal Formation.” Nature Neuroscience 2, no. 3: 260–265. 10195219 10.1038/6365

[jnc70448-bib-0060] Green, C. R. , S. Corsi‐Travali , and A. Neumeister . 2013. “The Role of BDNF‐TrkB Signaling in the Pathogenesis of PTSD.” Journal of Depression and Anxiety 2013, no. S4: 006. 25226879 10.4172/2167-1044.S4-006 PMC4161201

[jnc70448-bib-0061] Guo, Y. , X. Zhu , M. Zeng , et al. 2021. “A Diet High in Sugar and Fat Influences Neurotransmitter Metabolism and Subsequently Affects Brain Function by Altering the Gut Microbiota.” Translational Psychiatry 11: 328. 34045460 10.1038/s41398-021-01443-2 PMC8160265

[jnc70448-bib-0062] Han, H. , R. Zhong , S. Zhang , et al. 2023. “Hydroxytyrosol Attenuates Diquat‐Induced Oxidative Stress by Activating Nrf2 Pathway and Modulating Colonic Microbiota in Mice.” Journal of Nutritional Biochemistry 113: 109256. 36572071 10.1016/j.jnutbio.2022.109256

[jnc70448-bib-0063] Hawley, D. F. , K. Morch , B. R. Christie , and J. L. Leasure . 2012. “Differential Response of Hippocampal Subregions to Stress and Learning.” PLoS One 7, no. 12: e53126. 23285257 10.1371/journal.pone.0053126 PMC3532167

[jnc70448-bib-0064] He, H. , H. He , L. Mo , Z. You , and J. Zhang . 2024. “Priming of Microglia With Dysfunctional Gut Microbiota Impairs Hippocampal Neurogenesis and Fosters Stress Vulnerability of Mice.” Brain, Behavior, and Immunity 115: 280–294. 37914097 10.1016/j.bbi.2023.10.031

[jnc70448-bib-0065] Herlemann, D. P. R. , M. Labrenz , K. Jürgens , S. Bertilsson , J. J. Waniek , and A. F. Andersson . 2011. “Transitions in Bacterial Communities Along the 2000 Km Salinity Gradient of the Baltic Sea.” ISME Journal 5, no. 10: 1571–1579. 21472016 10.1038/ismej.2011.41 PMC3176514

[jnc70448-bib-0066] Hong, I. , and B. K. Kaang . 2022. “The Complexity of Ventral CA1 and Its Multiple Functionalities.” Genes, Brain, and Behavior 21, no. 2: e12826. 35815710 10.1111/gbb.12826 PMC9744572

[jnc70448-bib-0067] Hori, H. , and Y. Kim . 2019. “Inflammation and Post‐Traumatic Stress Disorder.” Psychiatry and Clinical Neurosciences 73, no. 3: 143–153. 30653780 10.1111/pcn.12820

[jnc70448-bib-0068] Huehnchen, P. , T. Prozorovski , P. Klaissle , et al. 2011. “Modulation of Adult Hippocampal Neurogenesis During Myelin‐Directed Autoimmune Neuroinflammation.” Glia 59, no. 1: 132–142. 20967885 10.1002/glia.21082

[jnc70448-bib-0069] Jessberger, S. , B. Römer , H. Babu , and G. Kempermann . 2005. “Seizures Induce Proliferation and Dispersion of Doublecortin‐Positive Hippocampal Progenitor Cells.” Experimental Neurology 196, no. 2: 342–351. 16168988 10.1016/j.expneurol.2005.08.010

[jnc70448-bib-0070] Kaelberer, M. M. , K. L. Buchanan , M. E. Klein , et al. 2018. “A Gut–Brain Neural Circuit for Nutrient Sensory Transduction.” Science 361, no. 6408: eaat5236. 30237325 10.1126/science.aat5236 PMC6417812

[jnc70448-bib-0071] Kamprath, K. , and C. T. Wotjak . 2004. “Nonassociative Learning Processes Determine Expression and Extinction of Conditioned Fear in Mice.” Learning & Memory 11, no. 6: 770–774. 15537742 10.1101/lm.86104 PMC534706

[jnc70448-bib-0072] Kee, N. , C. M. Teixeira , A. H. Wang , and P. W. Frankland . 2007. “Preferential Incorporation of Adult‐Generated Granule Cells Into Spatial Memory Networks in the Dentate Gyrus.” Nature Neuroscience 10, no. 3: 355–362. 17277773 10.1038/nn1847

[jnc70448-bib-0073] Kempermann, G. , H. G. Kuhn , and F. H. Gage . 1998. “Experience‐Induced Neurogenesis in the Senescent Dentate Gyrus.” Journal of Neuroscience 18, no. 9: 3206–3212. 9547229 10.1523/JNEUROSCI.18-09-03206.1998 PMC6792643

[jnc70448-bib-0074] Kheirbek, M. A. , L. J. Drew , N. S. Burghardt , et al. 2013. “Differential Control of Learning and Anxiety Along the Dorsoventral Axis of the Dentate Gyrus.” Neuron 77, no. 5: 955–968. 23473324 10.1016/j.neuron.2012.12.038 PMC3595120

[jnc70448-bib-0075] Kimura, H. 2010. “Hydrogen Sulfide: From Brain to Gut.” Antioxidants & Redox Signaling 12, no. 9: 1111–1123. 19803743 10.1089/ars.2009.2919

[jnc70448-bib-0076] Kjelstrup, K. G. , F. A. Tuvnes , H. A. Steffenach , R. Murison , E. I. Moser , and M. B. Moser . 2002. “Reduced Fear Expression After Lesions of the Ventral Hippocampus.” Proceedings of the National Academy of Sciences of the United States of America 99, no. 16: 10825–10830. 12149439 10.1073/pnas.152112399 PMC125057

[jnc70448-bib-0077] Knuesel, T. , and M. H. Mohajeri . 2021. “The Role of the Gut Microbiota in the Development and Progression of Major Depressive and Bipolar Disorder.” Nutrients 14: 37. 35010912 10.3390/nu14010037 PMC8746924

[jnc70448-bib-0078] Komitova, M. , and P. S. Eriksson . 2004. “Sox‐2 Is Expressed by Neural Progenitors and Astroglia in the Adult Rat Brain.” Neuroscience Letters 369, no. 1: 24–27. 15380301 10.1016/j.neulet.2004.07.035

[jnc70448-bib-0079] Konstantinidou, V. , M. I. Covas , D. Muñoz‐Aguayo , et al. 2010. “In Vivo Nutrigenomic Effects of Virgin Olive Oil Polyphenols Within the Frame of the Mediterranean Diet: A Randomized Controlled Trial.” FASEB Journal 24, no. 7: 2546–2557. 20179144 10.1096/fj.09-148452

[jnc70448-bib-0080] Kronenberg, G. , K. Reuter , B. Steiner , et al. 2003. “Subpopulations of Proliferating Cells of the Adult Hippocampus Respond Differently to Physiologic Neurogenic Stimuli.” Journal of Comparative Neurology 467, no. 4: 455–463. 14624480 10.1002/cne.10945

[jnc70448-bib-0081] Kundu, P. , H. U. Lee , I. Garcia‐Perez , et al. 2019. “Neurogenesis and Prolongevity Signaling in Young Germ‐Free Mice Transplanted With the Gut Microbiota of Old Mice.” Science Translational Medicine 11, no. 518: eaau4760. 31723038 10.1126/scitranslmed.aau4760

[jnc70448-bib-0082] Laghezza Masci, V. , R. Bernini , N. Villanova , et al. 2022. “In Vitro Anti‐Proliferative and Apoptotic Effects of Hydroxytyrosyl Oleate on SH‐SY5Y Human Neuroblastoma Cells.” International Journal of Molecular Sciences 23, no. 20: 12348. 36293207 10.3390/ijms232012348 PMC9604296

[jnc70448-bib-0083] Li, J. , C. Fan , J. Wang , et al. 2024. “Association Between Gut Microbiota and Anxiety Disorders: A Bidirectional Two‐Sample Mendelian Randomization Study.” BMC Psychiatry 24: 398. 38802804 10.1186/s12888-024-05824-x PMC11131207

[jnc70448-bib-0084] Liu, C. , S. Y. Yang , L. Wang , and F. Zhou . 2022. “The Gut Microbiome: Implications for Neurogenesis and Neurological Diseases.” Neural Regeneration Research 17, no. 1: 53–58. 34100427 10.4103/1673-5374.315227 PMC8451566

[jnc70448-bib-0085] Liu, H. , Z. Yang , C. Yu , et al. 2023. “Tau Aggravates Stress‐Induced Anxiety by Inhibiting Adult Ventral Hippocampal Neurogenesis in Mice.” Cerebral Cortex 33, no. 8: 3853–3865. 36047921 10.1093/cercor/bhac312

[jnc70448-bib-0086] Liu, R. T. , A. D. Rowan‐Nash , A. E. Sheehan , et al. 2020. “Reductions in Anti‐Inflammatory Gut Bacteria Are Associated With Depression in a Sample of Young Adults.” Brain, Behavior, and Immunity 88: 308–324. 32229219 10.1016/j.bbi.2020.03.026 PMC7415740

[jnc70448-bib-0087] Liu, X. , B. Mao , J. Gu , et al. 2021. “Blautia—A New Functional Genus With Potential Probiotic Properties?” Gut Microbes 13, no. 1: 1–21. 10.1080/19490976.2021.1875796 PMC7872077 33525961

[jnc70448-bib-0088] Ma, X. , Y. J. Shin , H. S. Park , et al. 2023. “ *Lactobacillus casei* and Its Supplement Alleviate Stress‐Induced Depression and Anxiety in Mice by the Regulation of BDNF Expression and NF‐κB Activation.” Nutrients 15, no. 11: 2488. 37299451 10.3390/nu15112488 PMC10255528

[jnc70448-bib-0089] Maeng, L. Y. , and M. R. Milad . 2017. “Post‐Traumatic Stress Disorder: The Relationship Between the Fear Response and Chronic Stress.” Chronic Stress 1: 247054701668970. 10.1177/2470547017713297 PMC7219872 32440579

[jnc70448-bib-0090] Malberg, J. E. , A. J. Eisch , E. J. Nestler , and R. S. Duman . 2000. “Chronic Antidepressant Treatment Increases Neurogenesis in Adult Rat Hippocampus.” Journal of Neuroscience 20, no. 24: 9104–9110. 11124987 10.1523/JNEUROSCI.20-24-09104.2000 PMC6773038

[jnc70448-bib-0091] Martinez‐Lapiscina, E. H. , P. Clavero , E. Toledo , et al. 2013. “Virgin Olive Oil Supplementation and Long‐Term Cognition: The PREDIMED‐Navarra Randomized Trial.” Journal of Nutrition, Health & Aging 17, no. 6: 544–552. 10.1007/s12603-013-0027-6 PMC12878917 23732551

[jnc70448-bib-0092] McAvoy, K. M. , and A. Sahay . 2017. “Targeting Adult Neurogenesis to Optimize Hippocampal Circuits in Aging.” Neurotherapeutics 14, no. 3: 630–645. 28536851 10.1007/s13311-017-0539-6 PMC5509633

[jnc70448-bib-0093] McGill, B. E. , R. A. Barve , S. E. Maloney , et al. 2018. “Abnormal Microglia and Enhanced Inflammation‐Related Gene Transcription in Mice With Conditional Deletion of Ctcf in Camk2a‐Cre‐Expressing Neurons.” Journal of Neuroscience 38, no. 21: 200–219. 29133437 10.1523/JNEUROSCI.0936-17.2017 PMC5761433

[jnc70448-bib-0094] McHugh, T. J. , M. W. Jones , J. J. Quinn , et al. 2007. “Dentate Gyrus NMDA Receptors Mediate Rapid Pattern Separation in the Hippocampal Network.” Science 317, no. 5834: 94–99. 17556551 10.1126/science.1140263

[jnc70448-bib-0095] Miao, F. 2022. “Hydroxytyrosol Alleviates Dextran Sodium Sulfate–Induced Colitis by Inhibiting NLRP3 Inflammasome Activation and Modulating Gut Microbiota in Vivo.” Nutrition 97: 111579. 35248848 10.1016/j.nut.2021.111579

[jnc70448-bib-0096] Micheli, L. , L. Bertini , A. Bonato , et al. 2023. “Role of Hydroxytyrosol and Oleuropein in the Prevention of Aging and Related Disorders: Focus on Neurodegeneration, Skeletal Muscle Dysfunction and Gut Microbiota.” Nutrients 15, no. 8: 1767. 37049607 10.3390/nu15071767 PMC10096778

[jnc70448-bib-0097] Micheli, L. , M. Ceccarelli , G. D'Andrea , et al. 2018. “Fluoxetine or Sox2 Reactivate Proliferation‐Defective Stem and Progenitor Cells of the Adult and Aged Dentate Gyrus.” Neuropharmacology 141: 316–330. 30142401 10.1016/j.neuropharm.2018.08.023

[jnc70448-bib-0098] Nagahara, N. , M. Nagano , T. Ito , K. Shimamura , T. Akimoto , and H. Suzuki . 2013. “Antioxidant Enzyme, 3‐Mercaptopyruvate Sulfurtransferase‐Knockout Mice Exhibit Increased Anxiety‐Like Behaviors: A Model for Human Mercaptolactate‐Cysteine Disulfiduria.” Scientific Reports 3: 1986. 23759691 10.1038/srep01986 PMC3680806

[jnc70448-bib-0099] Nagpure, B. V. , and J. S. Bian . 2015. “Brain, Learning, and Memory: Role of H_2_S in Neurodegenerative Diseases.” In Handbook of Experimental Pharmacology, vol. 230, 193–215. Springer. 26162836 10.1007/978-3-319-18144-8_10

[jnc70448-bib-0100] Nair, A. B. , and S. Jacob . 2016. “A Simple Practice Guide for Dose Conversion Between Animals and Human.” Journal of Basic and Clinical Pharmacy 7, no. 2: 27–31. 27057123 10.4103/0976-0105.177703 PMC4804402

[jnc70448-bib-0101] Nardiello, P. , D. Pantano , A. Lapucci , M. Stefani , and F. Casamenti . 2018. “Diet Supplementation With Hydroxytyrosol Ameliorates Brain Pathology and Restores Cognitive Functions in a Mouse Model of Amyloid‐β Deposition.” Journal of Alzheimer's Disease 63, no. 4: 1161–1172. 10.3233/JAD-171124 29710709

[jnc70448-bib-0102] Norden, D. M. , and J. P. Godbout . 2013. “Review: Microglia of the Aged Brain: Primed To Be Activated and Resistant to Regulation.” Neuropathology and Applied Neurobiology 39, no. 1: 19–34. 23039106 10.1111/j.1365-2990.2012.01306.x PMC3553257

[jnc70448-bib-0103] O'Leary, O. F. , and J. F. Cryan . 2014. “A Ventral View on Antidepressant Action: Roles for Adult Hippocampal Neurogenesis Along the Dorsoventral Axis.” Trends in Pharmacological Sciences 35, no. 8: 675–687. 25455365 10.1016/j.tips.2014.09.011

[jnc70448-bib-0104] Omar, S. H. , C. J. Scott , A. S. Hamlin , and H. K. Obied . 2018. “Biophenols: Enzymes (β‐Secretase, Cholinesterases, Histone Deacetylase and Tyrosinase) Inhibitors From Olive ( *Olea europaea* L.).” Fitoterapia 128: 118–129. 29772299 10.1016/j.fitote.2018.05.011

[jnc70448-bib-0105] Palmer, J. M. , M. A. Jusino , M. T. Banik , and D. L. Lindner . 2018. “Non‐Biological Synthetic Spike‐In Controls and the AMPtk Software Pipeline Improve Mycobiome Data.” PeerJ 6: e4925. 29868296 10.7717/peerj.4925 PMC5978393

[jnc70448-bib-0106] Peng, S. , B. Zhang , J. Yao , D. Duan , and J. Fang . 2015. “Dual Protection of Hydroxytyrosol, an Olive Oil Polyphenol, Against Oxidative Damage in PC12 Cells.” Food & Function 6, no. 6: 2091–2100. 26037629 10.1039/c5fo00097a

[jnc70448-bib-0141] Pennington, Z. T., Z. , Y. Dong , L. M. Feng , et al. 2019. “ezTrack: An Open‐Source Video Analysis Pipeline for the Investigation of Animal Behavior.” Scientific Reports 9, no. 1: 19979. 31882950 10.1038/s41598-019-56408-9 PMC6934800

[jnc70448-bib-0107] Platel, J. C. , K. A. Dave , V. Gordon , B. Lacar , M. E. Rubio , and A. Bordey . 2010. “NMDA Receptors Activated by Subventricular Zone Astrocytic Glutamate Are Critical for Neuroblast Survival Prior to Entering a Synaptic Network.” Neuron 65, no. 6: 859–872. 20346761 10.1016/j.neuron.2010.03.009 PMC2861893

[jnc70448-bib-0108] Porter, G. A. , and J. C. O'Connor . 2022. “Brain‐Derived Neurotrophic Factor and Inflammation in Depression: Pathogenic Partners in Crime?” World Journal of Psychiatry 12, no. 1: 77–97. 35111580 10.5498/wjp.v12.i1.77 PMC8783167

[jnc70448-bib-0109] Quesseveur, G. , D. J. David , M. C. Gaillard , et al. 2013. “BDNF Overexpression in Mouse Hippocampal Astrocytes Promotes Local Neurogenesis and Elicits Anxiolytic‐Like Activities.” Translational Psychiatry 3, no. 4: e253. 23632457 10.1038/tp.2013.30 PMC3641417

[jnc70448-bib-0110] Radjabzadeh, D. , J. A. Bosch , A. G. Uitterlinden , et al. 2022. “Gut Microbiome‐Wide Association Study of Depressive Symptoms.” Nature Communications 13, no. 1: 7128. 10.1038/s41467-022-34502-3 PMC9726982 36473852

[jnc70448-bib-0111] Raederstorff, D. 2009. “Antioxidant Activity of Olive Polyphenols in Humans: A Review.” International Journal for Vitamin and Nutrition Research 79, no. 3: 152–165. 20209466 10.1024/0300-9831.79.3.152

[jnc70448-bib-0112] Rolls, E. T. , and R. P. Kesner . 2006. “A Computational Theory of Hippocampal Function, and Empirical Tests of the Theory.” Progress in Neurobiology 79, no. 1: 1–48. 16781044 10.1016/j.pneurobio.2006.04.005

[jnc70448-bib-0113] Romani, A. , F. Ieri , S. Urciuoli , et al. 2019. “Health Effects of Phenolic Compounds Found in Extra‐Virgin Olive Oil, By‐Products, and Leaf of *Olea europaea* L.” Nutrients 11, no. 8: 1776. 31374907 10.3390/nu11081776 PMC6724211

[jnc70448-bib-0114] Santarelli, L. , M. Saxe , C. Gross , et al. 2003. “Requirement of Hippocampal Neurogenesis for the Behavioral Effects of Antidepressants.” Science 301, no. 5634: 805–809. 12907793 10.1126/science.1083328

[jnc70448-bib-0115] Segata, N. , J. Izard , L. Waldron , et al. 2011. “Metagenomic Biomarker Discovery and Explanation.” Genome Biology 12, no. 6: R60. 21702898 10.1186/gb-2011-12-6-r60 PMC3218848

[jnc70448-bib-0116] Sen, P. , E. Sherwin , K. Sandhu , et al. 2022. “The Live Biotherapeutic *Blautia stercoris* MRx0006 Attenuates Social Deficits, Repetitive Behaviour, and Anxiety‐Like Behaviour in a Mouse Model Relevant to Autism.” Brain, Behavior, and Immunity 106: 115–126. 35995237 10.1016/j.bbi.2022.08.007

[jnc70448-bib-0117] Seo, D. O. , M. A. Carillo , S. C. H. Lim , K. F. Tanaka , and M. R. Drew . 2015. “Adult Hippocampal Neurogenesis Modulates Fear Learning Through Associative and Nonassociative Mechanisms.” Journal of Neuroscience 35, no. 28: 11330–11345. 26269640 10.1523/JNEUROSCI.0483-15.2015 PMC4532761

[jnc70448-bib-0118] Seri, B. , J. M. García‐Verdugo , B. S. McEwen , and A. Alvarez‐Buylla . 2001. “Astrocytes Give Rise to New Neurons in the Adult Mammalian Hippocampus.” Journal of Neuroscience 21, no. 18: 7153–7160. 11549726 10.1523/JNEUROSCI.21-18-07153.2001 PMC6762987

[jnc70448-bib-0119] Shen, X. , M. Carlström , S. Borniquel , C. Jädert , C. G. Kevil , and J. O. Lundberg . 2013. “Microbial Regulation of Host Hydrogen Sulfide Bioavailability and Metabolism.” Free Radical Biology and Medicine 60: 195–200. 23466556 10.1016/j.freeradbiomed.2013.02.024 PMC4077044

[jnc70448-bib-0120] Shohayeb, B. , M. Diab , M. Ahmed , and D. C. H. Ng . 2018. “Factors That Influence Adult Neurogenesis as Potential Therapy.” Translational Neurodegeneration 7: 4. 29484176 10.1186/s40035-018-0109-9 PMC5822640

[jnc70448-bib-0121] Siddiqui, R. , M. R. Mungroo , A. M. Alharbi , H. Alfahemi , and N. A. Khan . 2022. “The Use of Gut Microbial Modulation Strategies as Interventional Strategies for Ageing.” Microorganisms 10, no. 9: 1869. 36144471 10.3390/microorganisms10091869 PMC9506335

[jnc70448-bib-0122] Siegmund, A. , and C. T. Wotjak . 2006. “Toward an Animal Model of Posttraumatic Stress Disorder.” Annals of the New York Academy of Sciences 1071: 324–334. 16891581 10.1196/annals.1364.025

[jnc70448-bib-0123] Siegmund, A. , and C. T. Wotjak . 2007a. “Hyperarousal Does Not Depend on Trauma‐Related Contextual Memory in an Animal Model of Posttraumatic Stress Disorder.” Physiology & Behavior 90, no. 1: 103–107. 17049568 10.1016/j.physbeh.2006.08.032

[jnc70448-bib-0124] Siegmund, A. , and C. T. Wotjak . 2007b. “A Mouse Model of Posttraumatic Stress Disorder That Distinguishes Between Conditioned and Sensitized Fear.” Journal of Psychiatric Research 41, no. 9: 848–860. 17027033 10.1016/j.jpsychires.2006.07.017

[jnc70448-bib-0125] Silva, Y. P. , A. Bernardi , and R. L. Frozza . 2020. “The Role of Short‐Chain Fatty Acids From Gut Microbiota in Gut‐Brain Communication.” Frontiers in Endocrinology 11: 25. 32082260 10.3389/fendo.2020.00025 PMC7005631

[jnc70448-bib-0126] Stangl, D. , and S. Thuret . 2009. “Impact of Diet on Adult Hippocampal Neurogenesis.” Genes & Nutrition 4, no. 4: 271–282. 19685256 10.1007/s12263-009-0134-5 PMC2775886

[jnc70448-bib-0127] Steiner, B. , F. Klempin , L. Wang , M. Kott , H. Kettenmann , and G. Kempermann . 2006. “Type‐2 Cells as Link Between Glial and Neuronal Lineage in Adult Hippocampal Neurogenesis.” Glia 54, no. 8: 805–814. 16958090 10.1002/glia.20407

[jnc70448-bib-0128] Strandwitz, P. 2018. “Neurotransmitter Modulation by the Gut Microbiota.” Brain Research 1693: 128–133. 29903615 10.1016/j.brainres.2018.03.015 PMC6005194

[jnc70448-bib-0129] Sun, T. , Q. Chen , S. Y. Zhu , et al. 2019. “Hydroxytyrosol Promotes Autophagy by Regulating SIRT1 Against Advanced Oxidation Protein Product‐Induced NADPH Oxidase and Inflammatory Response.” International Journal of Molecular Medicine 44, no. 5: 1531–1540. 31432093 10.3892/ijmm.2019.4300

[jnc70448-bib-0130] Tirone, F. , S. Farioli‐Vecchioli , L. Micheli , M. Ceccarelli , and L. Leonardi . 2013. “Genetic Control of Adult Neurogenesis: Interplay of Differentiation, Proliferation and Survival Modulates New Neurons' Function and Memory Circuits.” Frontiers in Cellular Neuroscience 7: 59. 23734097 10.3389/fncel.2013.00059 PMC3653098

[jnc70448-bib-0131] van Praag, H. , B. R. Christie , T. J. Sejnowski , and F. H. Gage . 1999. “Running Enhances Neurogenesis, Learning, and Long‐Term Potentiation in Mice.” Proceedings of the National Academy of Sciences of the United States of America 96, no. 23: 13427–13431. 10557337 10.1073/pnas.96.23.13427 PMC23964

[jnc70448-bib-0132] Velotti, F. , and R. Bernini . 2023. “Hydroxytyrosol Interference With Inflammaging via Modulation of Inflammation and Autophagy.” Nutrients 15, no. 1: 1774. 37049611 10.3390/nu15071774 PMC10096543

[jnc70448-bib-0133] Vivar, C. 2015. “Adult Hippocampal Neurogenesis, Aging and Neurodegenerative Diseases: Possible Strategies to Prevent Cognitive Impairment.” Current Topics in Medicinal Chemistry 15, no. 21: 2175–2192. 26059358 10.2174/1568026615666150610141524

[jnc70448-bib-0134] Wang, M. , J.‐J. Tang , L.‐X. Wang , J. Yu , L. Zhang , and C. Qiao . 2021. “Hydrogen Sulfide Enhances Adult Neurogenesis in a Mouse Model of Parkinson's Disease.” Neural Regeneration Research 16, no. 7: 1353–1358. 33318417 10.4103/1673-5374.301026 PMC8284305

[jnc70448-bib-0135] Wu, M. V. , and R. Hen . 2014. “Functional Dissociation of Adult‐Born Neurons Along the Dorsoventral Axis of the Dentate Gyrus.” Hippocampus 24, no. 7: 751–761. 24550158 10.1002/hipo.22265 PMC4222246

[jnc70448-bib-0136] Wu, M. V. , V. M. Luna , and R. Hen . 2015. “Running Rescues a Fear‐Based Contextual Discrimination Deficit in Aged Mice.” Frontiers in Systems Neuroscience 9: 114. 26321926 10.3389/fnsys.2015.00114 PMC4531235

[jnc70448-bib-0137] Yu, S. , W. Zhang , X. Wang , et al. 2024. “H_2_S Improves Hippocampal Synaptic Plasticity in SPS Rats via PI3K/AKT Signaling Pathway.” Brain Research 1845: 149286. 39433117 10.1016/j.brainres.2024.149286

[jnc70448-bib-0138] Zhang, Y. , K. Li , Y. Zhan , et al. 2023. “Bacteremia Caused by *Comamonas kerstersii* in a Patient With Acute Perforated Appendicitis and Localized Peritonitis: Case Report and Literature Review.” Frontiers in Medicine 10: 1283769. 38131046 10.3389/fmed.2023.1283769 PMC10733445

[jnc70448-bib-0139] Zhang, Y. J. , X. Chen , L. Zhang , et al. 2019. “Protective Effects of 3,4‐Dihydroxyphenylethanol on Spinal Cord Injury‐Induced Oxidative Stress and Inflammation.” Neuroreport 30, no. 12: 1016–1024. 31503208 10.1097/WNR.0000000000001318

[jnc70448-bib-0140] Zheng, P. , J. Yang , Y. Li , et al. 2020. “Gut Microbial Signatures can Discriminate Unipolar From Bipolar Depression.” Advanced Science 7, no. 7: 1902862. 32274300 10.1002/advs.201902862 PMC7140990

