# Supplementary material for: Hydroxytyrosol Mitigates Anxiety‐Like Behaviors After a Traumatic Experience in Aged Mice in Parallel With Increased Neurogenesis in the Ventral and Dorsal Dentate Gyrus, and Preservation of Gut Microbiota Composition
Source: J Neurochem. 2026 Apr 30;170:e70448. doi: 10.1111/jnc.70448 (PMC13131041; doi:10.1111/jnc.70448)
Supplement: Supplementary file 1 — Figure S1: Morphological analysis of DCX‐positive cells of mice treated as described in Figure 2A. Skeleton and Sholl analyses revealed a significant increase in the number (#) of branches and junctions in mice treated with HTyr, particularly in the ventral region of the dentate gyrus. Continuous outcomes (dendritic length) were analyzed using a LMM statistical analysis with a random intercept for Mouse; fixed‐effect significance was assessed using Wald F‐tests. Count outcomes were analyzed using GLMM‐NB statistical analysis and a random intercept for Mouse; significance was assessed using Wald χ 2 test. Planned simple‐effects analyses were performed to evaluate the effect of treatment within each region. Data are means ± SEM. n = 82 DCX+ cells. At least, 9 DCX+ cells/mouse were analyzed. Each group consisted of 4 mice. *p < 0.05, **p < 0.01, NS p > 0.05. LMM, linear mixed models; GLMM‐NB, generalized linear mixed models with a negative binomial distribution. Figure S2: (A) Schematic representation of the experimental plan. Percentage of freezing showed by HTyr (n = 9, red) and H2O (n = 9, blue) treated mice in (B) the contextual fear conditioning tests (1 and 16 days after training) and in (C) the context generalization tests in the two decidedly different contexts C and D (see text for details in the procedure). All the data are shown as mean ± SEM. Statistical analyses were carried out by two‐way ANOVA for contextual memory tests, and Two‐tailed Student's t‐test for context generalization tests. NS, p > 0.05. OF, Open Field; PM, plus maze; squares A and B: context A and B; circles A and B: context C and D. Figure S3: (A) Boxplots of Richness, Simpson, Shannon, and Evenness indexes in BT− mice for H2O and HTyr treatment at the initial (Ti) and final (Tf) time points. No statistically significant differences between experimental groups were detected based on the Kruskal–Wallis test. Richness: H = 6.5146, df = 3, p = 0.08909; Simpson index: H = 7.7068, df = 3, p = 0 [file JNC-170-e70448-s001.pdf]

Title: Hydroxytyrosol mitigates anxiety-like behaviors after a traumatic experience in aged mice in parallel with increased neurogenesis in the ventral and dorsal dentate gyrus, and preservation of gut microbiota composition

Authors: Giorgio D'Andrea[1,†], Laura Bertini[2,†], Marco Costanzi[3,†], Fabiana Canini[2], Roberta Bernini[4], Andrea Fochetti[4], Mariangela Clemente[4], Silvia Proietti[2], Manuela Ceccarelli[1,5], Carla Caruso[2,6], Maurizia Caruso[1], Ferdinando Scavizzi[7], Marcello Raspa[7], Felice Tirone[1,*], and Laura Micheli[1,*]

Affiliations:

[1] Institute of Biochemistry and Cell Biology (IBBC), National Research Council of Italy (CNR), c/o International Campus "A. Buzzati-Traverso", Via E. Ramarini, 32, 00015, Monterotondo Scalo (Rome), Italy.

[2] Department of Ecological and Biological Sciences, University of Tuscia, Largo Dell'Università, 01100, Viterbo, Italy

[3] Department of Human Sciences, LUMSA University, Piazza delle Vaschette 101, 00193 Rome, Italy

[4] Department of Agriculture and Forest Sciences (DAFNE), University of Tuscia, Via San Camillo de Lellis, 01100 Viterbo, Italy.

[5] Onco-Hematology, Cell Therapy, Gene Therapies and Hemopoietic Transplant, Bambino Gesù Children's Hospital IRCCS, Piazza Sant'Onofrio, 4, 00165, Rome, Italy.

[6] Institute for Sustainable Plant Protection, National Research Council of Italy, Strada delle Cacce, 73, Torino, 10135, Italy

[7] Institute of Biochemistry and Cell Biology, National Research Council of Italy (IBBC-CNR/EMMA/INFRAFRONTIER/IMPC), c/o International Campus "A. Buzzati-Traverso", Via E. Ramarini, 32, 00015, Monterotondo Scalo (Rome), Italy.

† These authors contributed equally to this work and share first authorship.

* Co-Corresponding authors:

Dr. Laura Micheli: E-mail: laura.micheli@cnr.it

Dr. Felice Tirone: E-mail: felice.tirone@cnr.it

	Whole DG			
**Measure**	**H_2O**	**HTyr**	**Model/ statistic**	**P value**
Total length (μm)	137.23 ± 12.91	156.24 ± 17.82	LMM F(1,5.9) = 0.29	NS
#Branches	3.80 ± 0.47	6.05 ± 0.66	GLMM χ^2 (1) = 5.91	*0.015
#Junctions	1.37 ± 0.21	2.39 ± 0.32	GLMM χ^2 (1) = 7.22	**0.007
Branching Index	15.44 ± 4.40	15.71 ± 3.70	GLMM χ^2 (1) = 0.049	NS

	Dorsal DG			
**Measure**	**H_2O**	**HTyr**	***Model/Statistic***	**P value**
Total length (μm)	156.27 ± 19.08	167.42 ± 26.88	LMM F(1,12.9)= 0.102	NS
#Branches	4.68 ± 0.73	6.44 ± 0.94	GLMM χ^2 (1) = 1.71	NS
#Junctions	1.74 ± 0.34	2.52 ± 0.43	GLMM χ^2 (1) = 1.73	NS
Branching Index	17.53 ± 5.72	19.65 ± 5.86	GLMM χ^2 (1) = 0.002	NS

	Ventral DG			
**Measure**	**H_2O**	**HTyr**	***P* Value**	***P* Value**
Total length (μm)	120.78 ± 26.88	141.96 ± 22.02	LMM F(1,13.8)= 0.31	NS
#Branches	3.05 ± 0.56	5.33 ± 0.93	GLMM χ^2 (1) = 5.87	*0.015
#Junctions	0.96 ± 0.24	2.00 ± 0.48	GLMM χ^2 (1) = 6.80	**0.009
Branching Index	13.64 ± 6.64	14.42 ± 3.74	GLMM χ^2 (1) = 0.075	NS

Figure S1. Morphological analysis of DCX-positive cells of mice treated as described in Figure 2A. Skeleton and Sholl analyses revealed a significant increase in the number (#) of branches and junctions in mice treated with HTyr, particularly in the ventral region of the dentate gyrus. Continuous outcomes (dendritic length) were analyzed using a LMM statistical analysis with a random intercept for Mouse; fixed-effect significance was assessed using Wald F-tests. Count outcomes were analyzed using GLMM-NB statistical analysis and a random intercept for Mouse; significance was assessed using Wald χ^2 test. Planned simple-effects analyses were performed to evaluate the effect of treatment within each region. Data are means ± SEM. n= 82 DCX^+ cells. At least, 9 DCX^+ cells/mouse were analyzed. Each group consisted of 4 mice. * $P < 0.05$, **$P < 0.01$, NS $P > 0.05$. LMM: Linear Mixed Models; GLMM-NB: Generalized Linear Mixed Models with a Negative Binomial distribution.

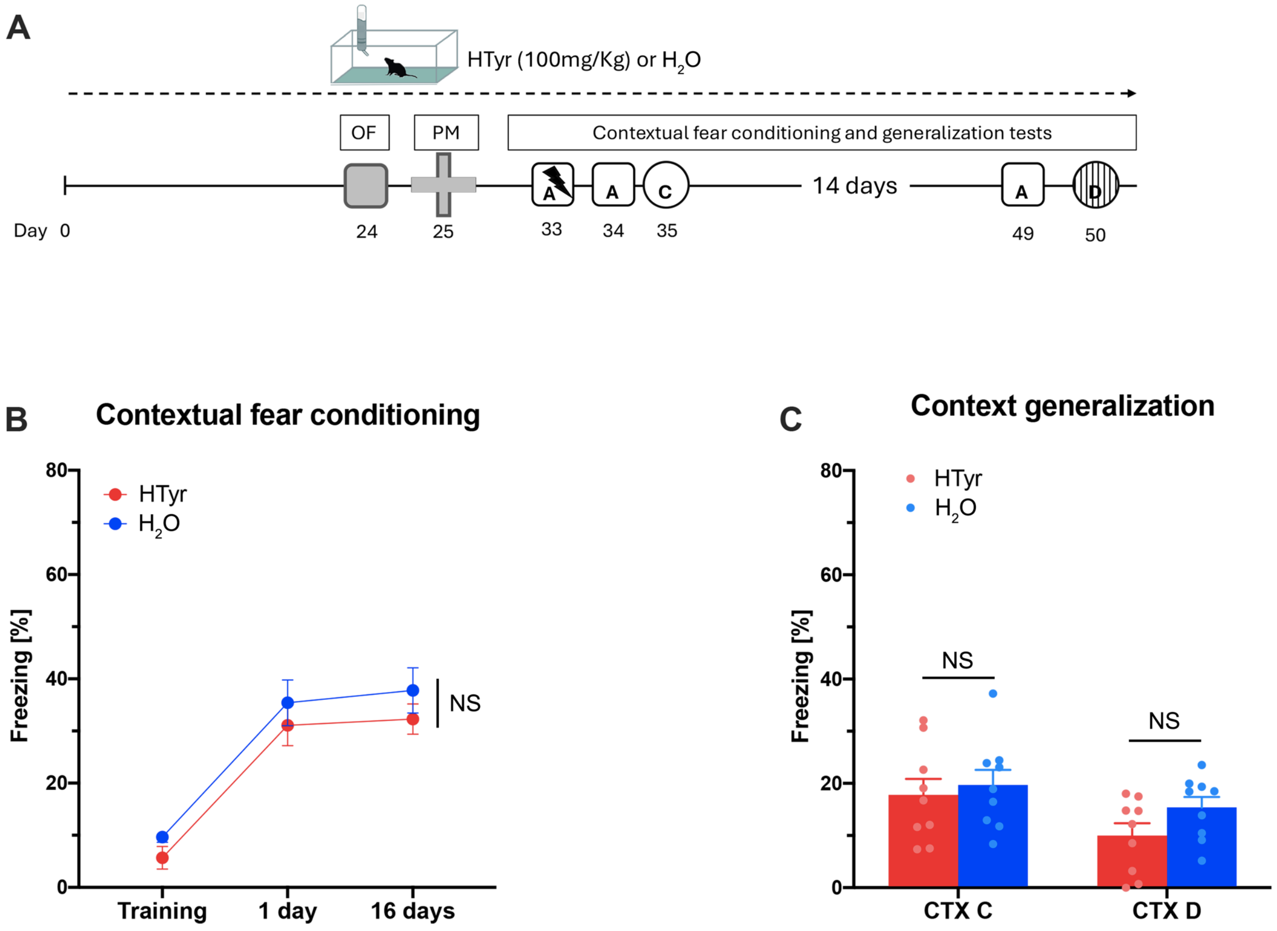

Figure S2. (**A**) Schematic representation of the experimental plan. Percentage of freezing showed by HTyr (n=9, red) and H_2O (n=9, blue) treated mice in (**B**) the contextual fear conditioning tests (1 and 16 days after training) and in (**C**) the context generalization tests in the two decidedly different contexts C and D (see text for details in the procedure). All the data are shown as mean ± SEM. Statistical analyses were carried out by two-way ANOVA for contextual memory tests, and Two-tailed Student's *t*-test for context generalization tests. NS, $P > 0.05$. OF: Open field; PM: Plus maze; squares A and B: context A and B; circles A and B: context C and D.

A

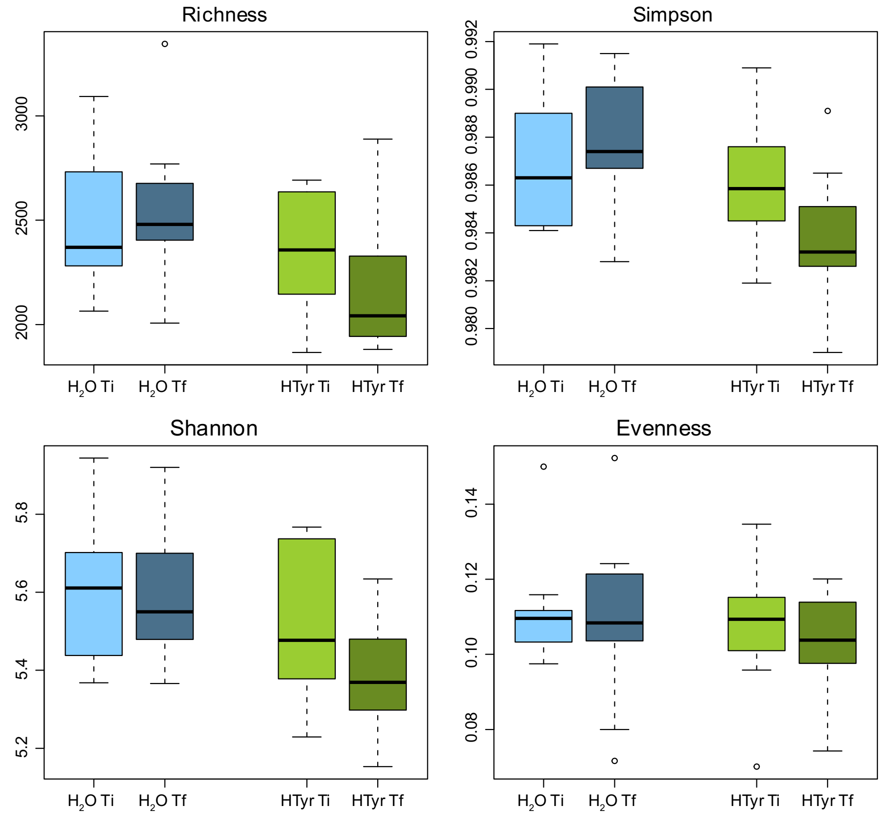

B

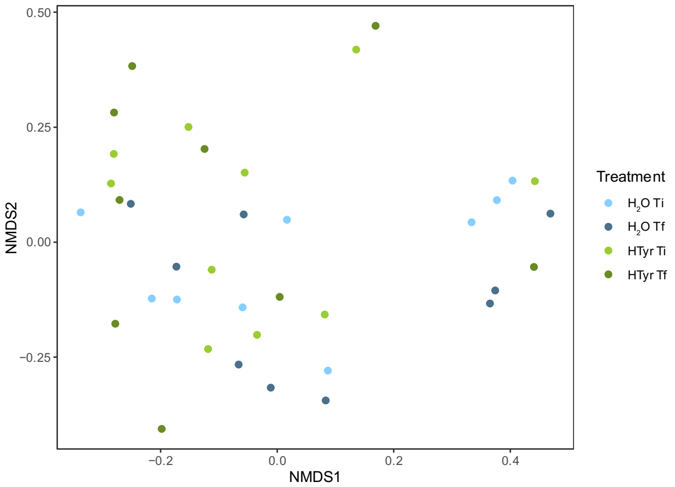

Figure S3. (A) Boxplots of Richness, Simpson, Shannon and Evenness indexes in BT- mice for H_2O and HTyr treatment at the initial (Ti) and final (Tf) time points. No statistically significant differences between experimental groups were detected based on the Kruskal-Wallis test. Richness: H= 6.5146, *df* = 3, *P*= 0.08909; Simpson index: H= 7.7068, *df* = 3, *P*= 0.05248; Shannon index: H= 6.505, *df* = 3, *P*= 0.08947; Evenness: H= 0.7315, *df* = 3, *P*= 0.8658. **(B)** NMDS ordinations of communities' composition in BT- mice using the Bray-Curtis distance metric of Hellinger transformed OUT abundances. Clustering significance was assessed by PERMANOVA. For all comparisons P>0.05. In the box plots, the line shows the median; the box, the interquartile range; the whiskers, the highest and lowest values; spare dots represent outlier.

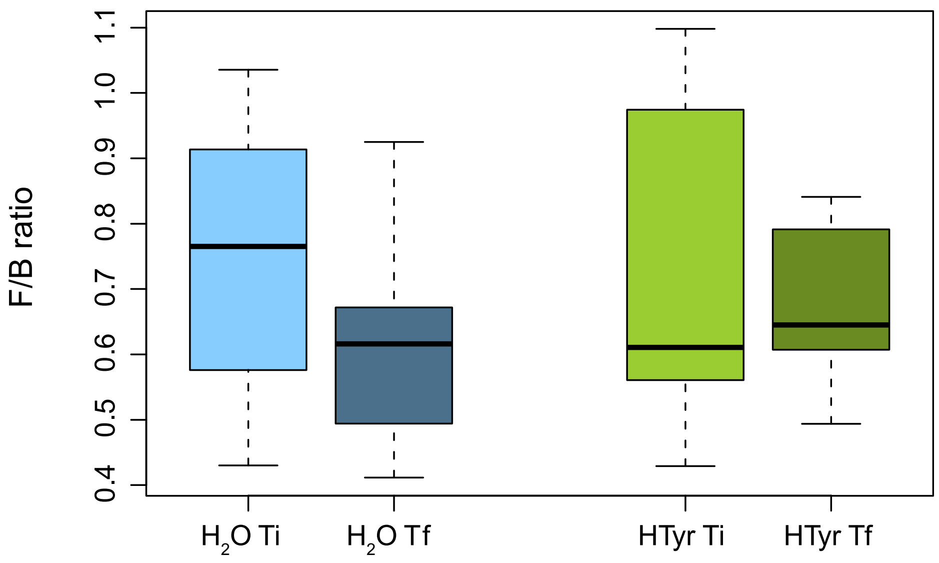

Figure S4. Boxplots of Firmicutes/Bacteroidetes ratio in BT- mice for H_2O and HTyr treatment at the initial (Ti) and final (Tf) time points. No statistically significant differences between samples were detected based on the Kruskal-Wallis test (H= 1.1163, *df* = 3, *P* = 0.7731). In the box plots, the line shows the median; the box, the interquartile range; the whiskers, the highest and lowest values; spare dots represent outliers.

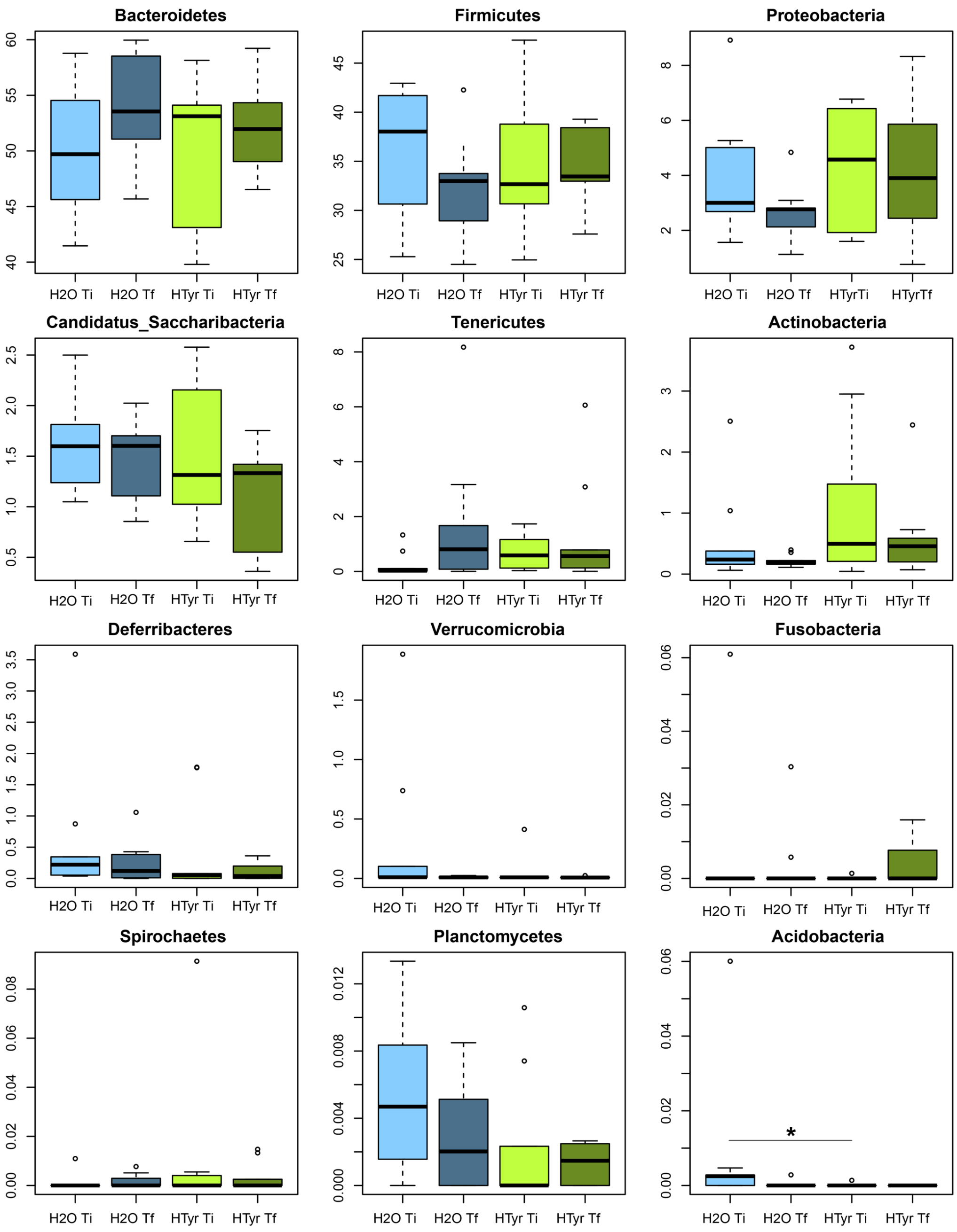

Figure S5. Boxplots of relative abundance of bacterial phyla in BT- mice for H_2O and HTyr treatment at the initial (Ti) and final (Tf) time points. Kruskal-Wallis test was performed to assess significant differences across treatments. The comparisons between groups were based on two-tailed Mann-Whitney *U* test. Acidobacteriaceae: H= 11.058, *df* = 3, *P* = 0.01142; U= 23, P= 0.02597. No significant phyla: *df* = 3, H $\leq$ 6.062, *P*> 0.05. Statistically significant differences are marked with asterisks (*, *P* <0.05, Mann-Whitney *U* test). In the box plots, the line shows the median; the box, the interquartile range; the whiskers, the highest and lowest values; spare dots represent outliers.

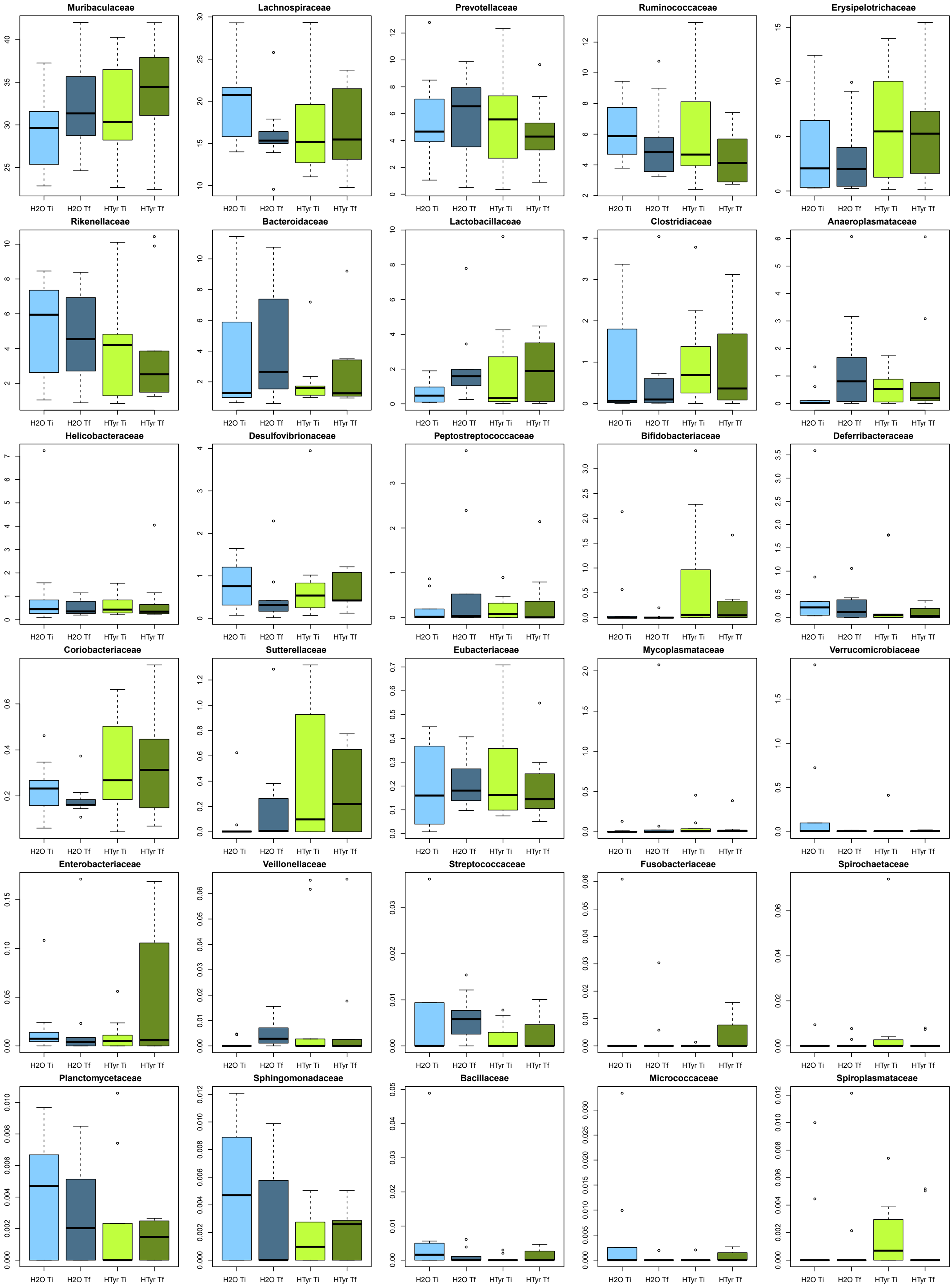

Figure S6. Boxplots of relative abundance of bacterial families in BT- mice for H_2O and HTyr treatment at the initial (Ti) and final (Tf) time points. No statistically significant differences between groups were detected based on Kruskal-Wallis test ($df = 3$, H ≤ 5.534, $P > 0.05$). In the box plots, the line shows the median; the box, the interquartile range; the whiskers, the highest and lowest values; spare dots represent outliers.

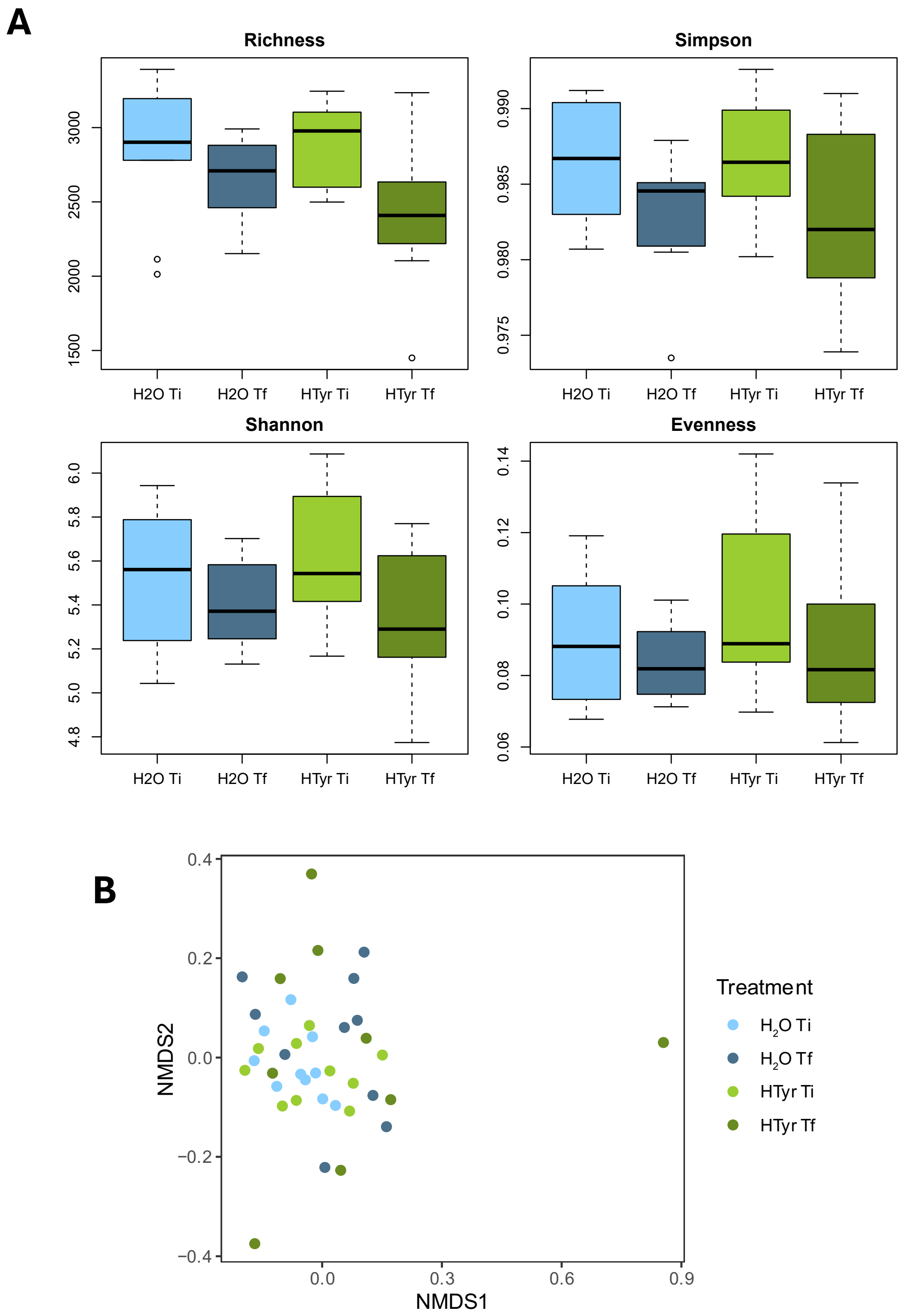

Figure S7. (A) Boxplots of Richness, Simpson, Shannon and Evenness indexes in BT+ mice for H_2O and HTyr treatment at the initial (Ti) and final (Tf) time points. No statistically significant differences between samples were detected based on Kruskal-Wallis test (Richness: H=5.8505, *df* = 3, *P*=0.119; Simpson index: H=6.075, *df* = 3, *P*= 0.108; Shannon index: H= 4.650, *df* = 3, *P*=0.199; Evenness: H= 2.739, *df* = 3, *P*=0.433). (B) NMDS ordinations of communities' composition in BT+ mice using the Bray-Curtis distance metric of Hellinger transformed OUT abundances. Clustering significance was assessed by PERMANOVA. For all comparisons P>0.05. In the box plots, the line shows the median; the box, the interquartile range; the whiskers, the highest and lowest values; spare dots represent outliers.

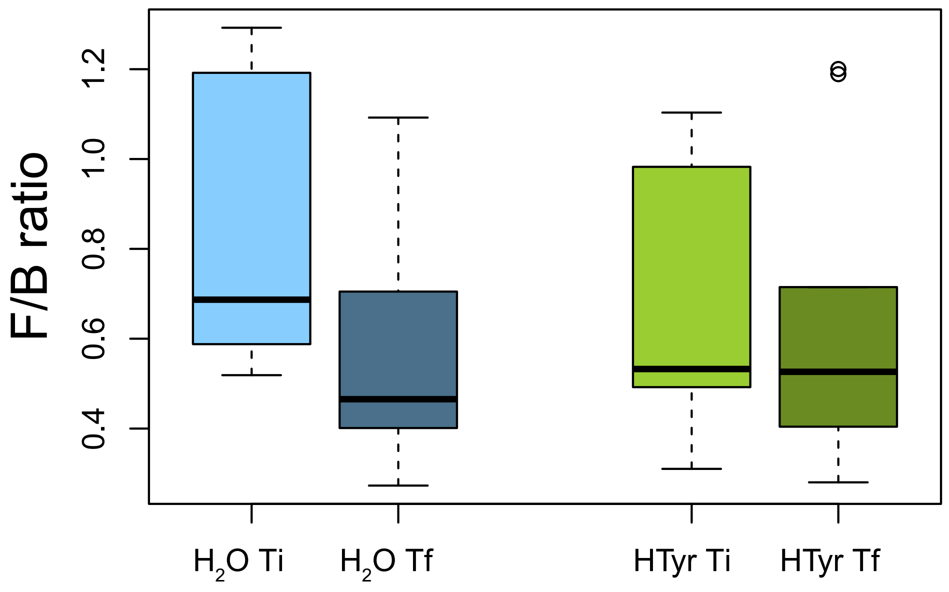

Figure S8. Boxplots of Firmicutes/Bacteroidetes ratio in BT+ mice for H_2O and HTyr treatment at the initial (Ti) and final (Tf) time points. No statistically significant differences between samples were detected based on Kruskal-Wallis test (H= 5.8416, *df* = 3, *P* = 0.1196). In the box plots, the line shows the median; the box, the interquartile range; the whiskers, the highest and lowest values; spare dots represent outliers.

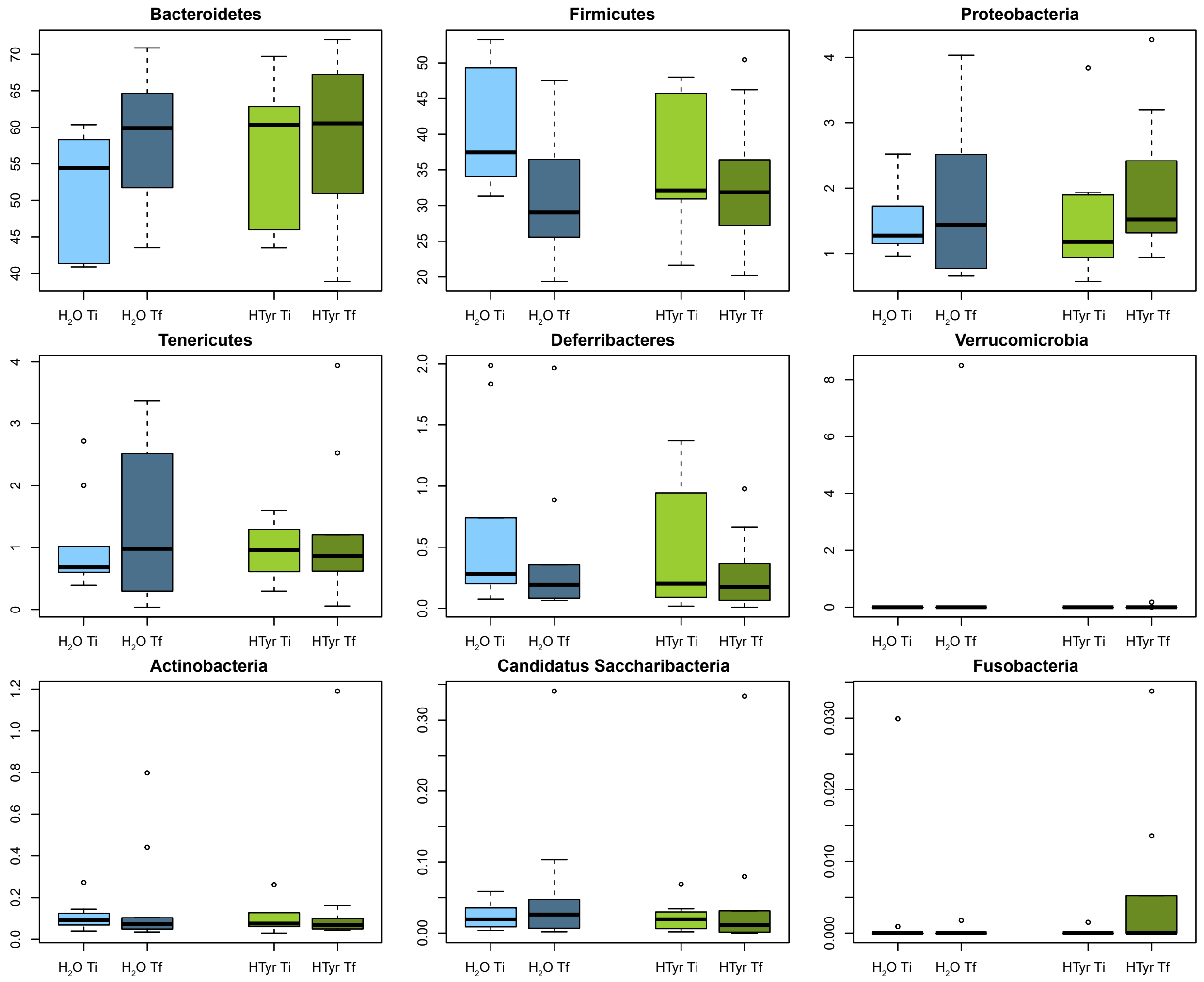

Figure S9. Boxplots of relative abundance of bacterial phyla in BT+ mice for H_2O and HTyr treatment at the initial (Ti) and final (Tf) time points. No statistically significant differences between samples were detected based on Kruskal-Wallis test (*df* = 3, H≤ 6.466, *P*> 0.05). In the box plots, the line shows the median; the box, the interquartile range; the whiskers, the highest and lowest values; spare dots represent outliers.

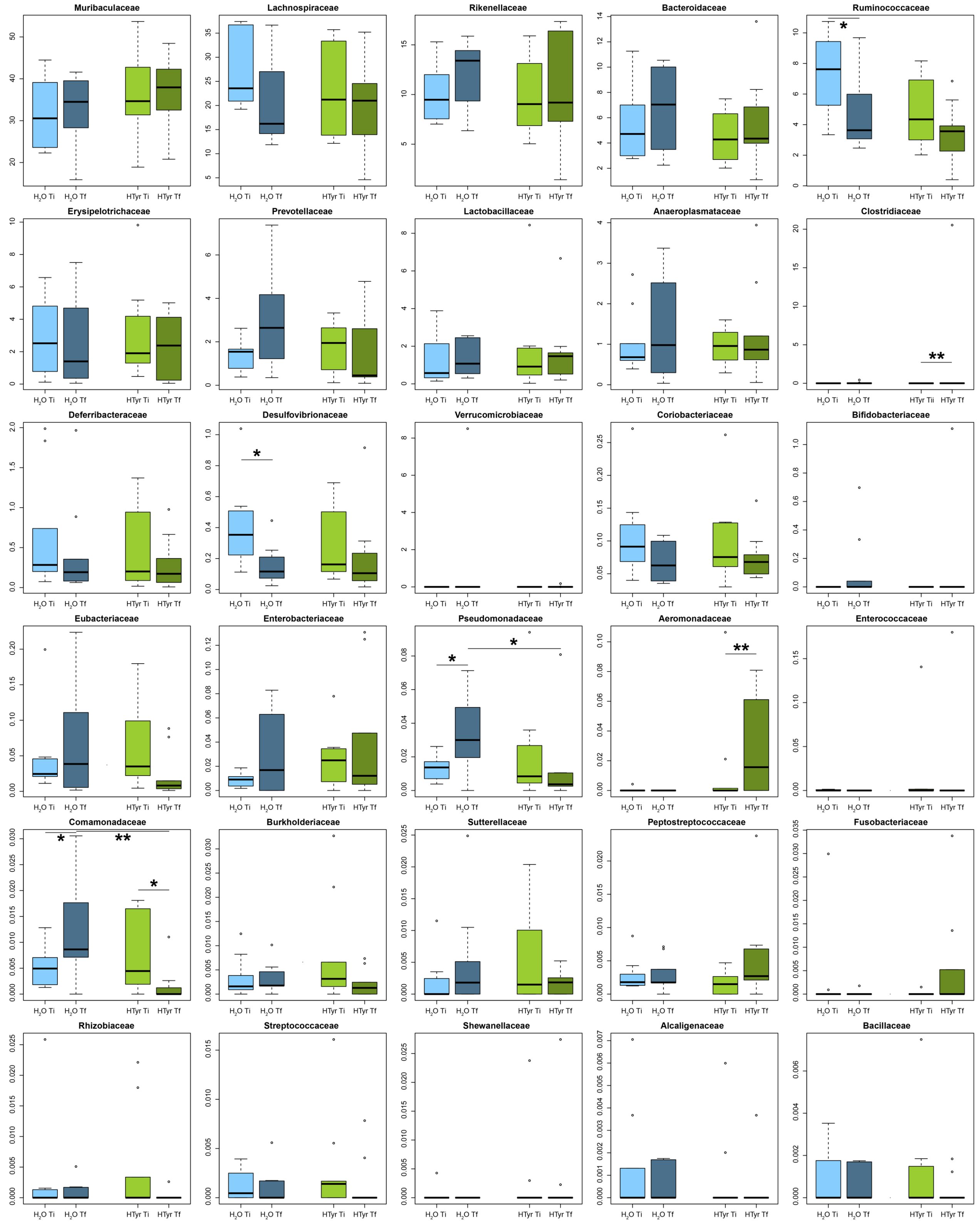

Figure S10. Boxplots of relative abundance of bacterial Families in BT+ mice for H2O and HTyr treatment at the initial (Ti) and final (Tf) time points. Kruskal-Wallis test was used to evaluate overall differences across treatments. For significant families, pairwise comparisons were performed using two-tailed Mann-Whitney *U* test. Ruminococcaceae: Kruskal-Wallis [*df*= 3; H=10.693] *P*= 0.01351; Mann-Whitney *U* test H2O Ti vs H2O Tf : *U*=27, *P*= 0.01726. Desulfovibrionaceae: Kruskal-Wallis [*df*= 3; H= 8.1937] *P*= 0.04217; Mann-Whitney *U* test H2O Ti vs H2O Tf: *U*= 29, *P*= 0.01133. Pseudomonadaceae: Kruskal-Wallis [*df*= 3; H= 8.1517] *P*= 0.04298; Mann-Whitney *U* test H2O Ti vs

H2O Tf: *U*= -34, *P*= 0.03121; H2O Tf vs HTyr Tf: *U*=17, *P*= 0.03019. Comamonadaceae: Kruskal-Wallis [*df*= 3; H= 12.894] *P*= 0.00487; Mann-Whitney *U* test H2O Ti vs H2O Tf: *U*= -34, *P*= 0.03121; HTyr Ti vs HTyr Tf: *U*= 18, *P*= 0.01965; H2O Tf vs HTyr Tf: *U*= 24, *P*= 0.00503. Aeromonadaceae: Kruskal-Wallis [*df*= 3; H= 13.234] *P*= 0.0042; Mann-Whitney *U* test HTyr Ti vs H2O Tf: *U*= -40, *P*= 0.00350. For families showing no significant differences: *df* = 3, H $\leq$ 6.044, *P*> 0.05. Statistically significant differences are marked with asterisks (*, P <0.05; **, P< 0.01, Mann-Whitney *U* test). In the box plots, the line shows the median; the box, the interquartile range; the whiskers, the highest and lowest values; spare dots represent outliers.
